# Comparative risk of thrombosis with thrombocytopenia syndrome or thromboembolic events associated with different covid-19 vaccines: international network cohort study from five European countries and the US

**DOI:** 10.1136/bmj-2022-071594

**Published:** 2022-10-26

**Authors:** Xintong Li, Edward Burn, Talita Duarte-Salles, Can Yin, Christian Reich, Antonella Delmestri, Katia Verhamme, Peter Rijnbeek, Marc A Suchard, Kelly Li, Mees Mosseveld, Luis H John, Miguel-Angel Mayer, Juan-Manuel Ramirez-Anguita, Catherine Cohet, Victoria Strauss, Daniel Prieto-Alhambra

**Affiliations:** 1 Centre for Statistics in Medicine, Nuffield Department of Orthopaedics, Rheumatology and Musculoskeletal Sciences, University of Oxford, Oxford, UK; 2 Fundació Institut Universitari per a la recerca a l’Atenció Primària de Salut Jordi Gol i Gurina (IDIAPJGol), Barcelona, Spain; 3 Real World Solutions, IQVIA, Durham, NC, USA; 4 Department of Medical Informatics, Erasmus University Medical Center, Rotterdam, Netherlands; 5 Department of Biostatistics, Fielding School of Public Health, University of California, Los Angeles, Los Angeles, CA, USA; 6 Department of Human Genetics, David Geffen School of Medicine at UCLA, University of California, Los Angeles, Los Angeles, CA, USA; 7 Research Programme on Biomedical Informatics, Hospital del Mar Medical Research Institute, Faculty of Health and Life Sciences, University of Pompeu Fabra, Barcelona, Spain; 8 Data Analytics and Methods Task Force, European Medicines Agency, Amsterdam, Netherlands

## Abstract

**Objective:**

To quantify the comparative risk of thrombosis with thrombocytopenia syndrome or thromboembolic events associated with use of adenovirus based covid-19 vaccines versus mRNA based covid-19 vaccines.

**Design:**

International network cohort study.

**Setting:**

Routinely collected health data from contributing datasets in France, Germany, the Netherlands, Spain, the UK, and the US.

**Participants:**

Adults (age ≥18 years) registered at any contributing database and who received at least one dose of a covid-19 vaccine (ChAdOx1-S (Oxford-AstraZeneca), BNT162b2 (Pfizer-BioNTech), mRNA-1273 (Moderna), or Ad26.COV2.S (Janssen/Johnson & Johnson)), from December 2020 to mid-2021.

**Main outcome measures:**

Thrombosis with thrombocytopenia syndrome or venous or arterial thromboembolic events within the 28 days after covid-19 vaccination. Incidence rate ratios were estimated after propensity scores matching and were calibrated using negative control outcomes. Estimates specific to the database were pooled by use of random effects meta-analyses.

**Results:**

Overall, 1 332 719 of 3 829 822 first dose ChAdOx1-S recipients were matched to 2 124 339 of 2 149 679 BNT162b2 recipients from Germany and the UK. Additionally, 762 517 of 772 678 people receiving Ad26.COV2.S were matched to 2 851 976 of 7 606 693 receiving BNT162b2 in Germany, Spain, and the US. All 628 164 Ad26.COV2.S recipients from the US were matched to 2 230 157 of 3 923 371 mRNA-1273 recipients. A total of 862 thrombocytopenia events were observed in the matched first dose ChAdOx1-S recipients from Germany and the UK, and 520 events after a first dose of BNT162b2. Comparing ChAdOx1-S with a first dose of BNT162b2 revealed an increased risk of thrombocytopenia (pooled calibrated incidence rate ratio 1.33 (95% confidence interval 1.18 to 1.50) and calibrated incidence rate difference of 1.18 (0.57 to 1.8) per 1000 person years). Additionally, a pooled calibrated incidence rate ratio of 2.26 (0.93 to 5.52) for venous thrombosis with thrombocytopenia syndrome was seen with Ad26.COV2.S compared with BNT162b2.

**Conclusions:**

In this multinational study, a pooled 30% increased risk of thrombocytopenia after a first dose of the ChAdOx1-S vaccine was observed, as was a trend towards an increased risk of venous thrombosis with thrombocytopenia syndrome after Ad26.COV2.S compared with BNT162b2. Although rare, the observed risks after adenovirus based vaccines should be considered when planning further immunisation campaigns and future vaccine development.

## Introduction

By May 2021, four covid-19 vaccines had been granted conditional marketing authorisation by the European Medicines Agency after showing high efficacy and safety in phase 3 clinical trials.^1^ ^2^ ^3^ ChAdOx1-S (Oxford-AstraZeneca) and Ad26.COV2.S (Janssen/Johnson & Johnson) are both adenovirus based vaccines. BNT162b2 (Pfizer-BioNTech) and mRNA-1273 (Moderna) are both mRNA based vaccines. After millions of vaccine doses were given in large scale immunisation campaigns, rare cases of thrombosis with thrombocytopenia syndrome were reported, often after the first dose of adenovirus vaccines.^4^ ^5^ ^6^ Although fewer concerns have been raised about the safety of mRNA vaccines, instances of immune thrombocytopenia have also been observed in recipients of BNT162b2.^7^

A causal relation between these vaccines and thrombosis with thrombocytopenia syndrome was considered by the EMA’s pharmacovigilance risk assessment committee, leading to an update of the product information for ChAdOx1-S to include thrombosis with thrombocytopenia syndrome as a very rare side effect.^8^ Because these unusual blood clots in combination with thrombocytopenia were reported predominantly in women aged under 60 years, several European countries restricted the use of adenovirus vaccines in younger age groups as a precautionary measure. While the pathogenesis is not yet fully understood, an immune response leading to the development of pathological platelet activating antibodies has been suggested and named as vaccine induced immune thrombotic thrombocytopenia.^6^ ^9^ Although these events are very rare, absolute numbers of affected patients could become substantial owing to the large numbers of vaccine doses administered worldwide.

Although some observational studies have examined the risk of thrombosis with thrombocytopenia syndrome after covid-19 vaccination in some European countries,^10^ ^11^ ^12^ ^13^ no clear evidence exists on the comparative safety profile of different vaccines. Given the high number of SARS-CoV-2 infections and reinfections seen worldwide, and the known effectiveness of covid-19 vaccines in minimising severe infection and complications, understanding the risks of the available vaccines compared with each other is essential, rather than comparing them with no vaccination. We therefore aimed to quantify the comparative risk of thrombosis with thrombocytopenia syndrome or thromboembolic events associated with the use of adenovirus based covid-19 vaccines versus mRNA based covid-19 vaccines.

## Methods

### Study design

We conducted an international network cohort study using routinely collected healthcare data mapped to the OMOP CDM (observational medical outcomes partnership common data model). The OMOP CDM allowed the study to be run by each site with common analytical code. Results were aggregated without sharing patient level data.^14^ ^15^ ^16^

### Data sources

Datasets from five European countries (France, Germany, the Netherlands, Spain, and the UK) and two datasets from the US informed the analyses. IQVIA Longitudinal Patient Data (LPD) France is a centralised anonymised patient electronic medical records database contributed by general practices.^17^ IQVIA Disease Analyser (DA) Germany is collected from extracts of patient management software used by general medicine and specialists practising in ambulatory care settings. The Integrated Primary Care Information (IPCI) database contains electronic healthcare records collected from patients registered with general practices in the Netherlands.^18^ The Information System for Research in Primary Care (SIDIAP) is a primary care records database that covers about 80% of the population of Catalonia, Spain. SIDIAP was linked to the regional vaccination registry and to hospital discharge data (CMBD-HA) for this study.^19^ The Clinical Practice Research Datalink (CPRD) Aurum database collects anonymised primary care electronic health records from general practices across the UK, which are linked at origin to national vaccination records.^20^ The IQVIA hospital charge data master (US Hospital CDM) dataset comprises records from hospital charge data master files from the US and records both inpatient and outpatient encounters.^21^ The US Open Claims dataset includes medical claims covering about 191 million people across the US, with patient level office visit, outpatient, and inpatient information (table 1).

**Table 1:** Descriptions of medical records databases used in study

Database full (short) names	Country	Active size of database (by mid-2021; No of people)	Latest data available time	Key data available				
				Covid-19 vaccines	Hospital treatments	Hospital outcomes	Outpatient treatments	Platelet counts
Clinical Practice Research Datalink Aurum (UK CPRD)	UK	13m	May 2021	Complete	No	Incomplete	Yes	Yes
Information System for Research in Primary Care with minimum basic set of hospital discharge data (CMBD-HA; Spain SIDIAP)	Spain	6m	June 2021	Complete	No	Linked	Yes	Yes
Integrated Primary Care Information (Netherlands IPCI)	The Netherlands	2m	June 2021	Incomplete	No	Incomplete	Yes	Yes
IQVIA Longitudinal Patient Data France (France LPD)	France	2.3m	September 2021	Incomplete	No	Incomplete	Yes	Yes
IQVIA Disease Analyser Germany (Germany DA)	Germany	8.5m	August 2021	Incomplete	No	Incomplete	Yes	Yes
Medical and Institutional Claims (US Open Claims)	US	187m	September 2021	Incomplete	Incomplete	Incomplete	Yes	Yes
Charge Data Master (US Hospital CDM)	US	30m	July 2021	Incomplete	Yes	Yes	Incomplete	Incomplete

The study period to identify vaccinations and outcomes started from December 2020 (first vaccines administered) to the latest data release available in each of the contributing databases (ie, mid-2021).

### Study participants

The study population were adults (aged 18 years or over at date of the first dose vaccination)registered in any of the contributing databases and exposed to at least one dose of a covid-19 vaccine during the study period. We required a minimum of one year of history available in the database before the index vaccination date. We excluded individuals who did not have a vaccine brand specified (unspecific vaccine codes) during the study period. We also excluded people who received their second dose within 14 days of the first dose, as these were likely errors in vaccination records. We included only people with complete records for age and sex.

Four covid-19 vaccines were included: ChAdOx1-S, BNT162b2, mRNA-1273, and Ad26.COV2.S. Vaccines were identified by procedure, drug, or observation codes in each database (supplementary B). We built first and second dose cohorts for each brand. In the second dose cohorts, we did not include individuals whose second dose vaccine brand was different from their first dose. A single dose cohort was built for Ad26.COV2.S as it was approved for a single dose schedule at the time of protocol approval. Comparisons were made between the adenovirus based vaccines (ChAdOx1-S or Ad26.COV2.S; ie, the target) and mRNA vaccines (BNT162b2 or mRNA-1273; ie, the comparator).

The index dates for the first and second dose vaccination cohorts were defined as the dates of the first and second covid-19 vaccinations for a specific brand, respectively. We followed individuals from their index date to 28 days after vaccination, death, or loss of visibility in the database (eg, person leaving the practice in electronic health records data, or end of continuous enrolment in claims data), whichever came first. The risk window of 28 days is based on the World Health Organization’s definition and the UK Medicines and Healthcare Products Regulatory Agency (MHRA) guidelines.^22^ ^23^ Owing to the expense of computing, we used a random sample of 20% of each cohort when using US Open Claims data.

### Primary outcomes

The primary outcomes were thromboembolic events and thrombosis with thrombocytopenia syndrome. Thromboembolic events of interest included deep vein thrombosis, pulmonary embolism, venous thromboembolism as a composite of deep vein thrombosis or pulmonary embolism, cerebral venous sinus thrombosis, splanchnic and visceral vein thrombosis ischaemic stroke, myocardial infarction, arterial thromboembolism as a composite of ischaemic stroke, and other rare arterial thromboembolisms such as intestinal infarction (supplementary B).

The definition of thrombosis with thrombocytopenia syndrome (supplementary B) was based on that proposed by the Brighton Collaboration and encompassed the occurrence of any thromboembolic event of interest with concurrent thrombocytopenia within 10 days before or after a thromboembolic event occurring within 28 days after vaccination. Thrombocytopenia was identified by a diagnostic code or measurement of <150 000 platelets per μL of blood, as proposed by the Brighton Collaboration.^24^ This definition has been used in previous OMOP CDM based studies.^25^

We used two alternative definitions for thrombosis with thrombocytopenia syndrome in sensitivity analyses. The first analysis required concurrent thrombocytopenia to have happened within five days before or after the thromboembolic event after vaccination. The second analysis reduced the threshold to <100 000 platelets/µL for the definition of thrombocytopenia, based on laboratory data.

### Negative control outcomes

Negative control outcomes are outcome events that are not expected to be causally associated with the vaccination. We used 92 negative control outcomes previously used for vaccine safety.^26^ They were prespecified on the basis of clinical knowledge and previous literature, validated by two clinicians, and tested in previous work on other vaccine safety projects.^27^ Supplementary A table 1 shows the codes for these negative control outcomes.

### Covariates

We defined baseline patient characteristics as potential confounders based on information recorded before index date, including personal data (age, sex, index year, and index month), clinical condition at any time before cohort index, composite comorbidity (Romano’s adapted Charlson Comorbidity Index^28^), and thrombosis score (CHA_2_DS_2_-VASc, congestive heart failure, hypertension, vascular disease^29^), and total number of medicines, procedures, and measurement records in the six months before the cohort index date.

### Statistical analysis

Descriptive statistics were used to report the baseline characteristics for each cohort. We reported the database specific incidence rate at 28 days and corresponding 95% confidence intervals for each event.

We matched on propensity scores to minimise observed confounding. We calculated propensity scores for each pair of vaccines being compared (target and comparator) using large scale L1 regularised logistic regression,^30^ which included all available baseline patient characteristics in the databases. The derived propensity score was used to match patients using greedy matching with a caliper width of 0.2 standard deviations of the logit at a ratio of up to 1:4. If the target cohort was larger than the comparator cohort, reverse matching was allowed, and a ratio of 4:1 was used.

We used three diagnostic tools to evaluate measured confounding, statistical power, and unmeasured confounding. We did not complete any database specific analysis that failed the measured confounding or statistical power diagnostics to avoid bias. Firstly, regarding measured confounding, only vaccine pairs of target and comparator with all covariates showing a standardised mean difference below 0.1 after the matching of propensity scores were considered satisfactory. Secondly, for statistical power, we calculated the minimum detectable rate ratio using α of 0.05 and power of 80% for each outcome of interest in both the crude cohorts and those matched with propensity scores.^31^

No estimates of incidence rate ratios specific to outcome were reported where the minimum detectable rate ratio was >5 for an outcome combination of database, target, and comparator, because a minimum detectable rate ratio >5 was deemed too underpowered making any such comparison unreliable. Thirdly, regarding unmeasured confounding, we studied associations with negative control outcomes to assess residual bias after matching propensity scores. We prespecified that <20% of negative control outcomes should be associated with vaccination to deem an analysis reliable in terms of residual confounding. Results for those that failed the unmeasured confounding diagnostic are reported, but only empirically calibrated estimates should be relied on (see below).

We used Poisson regression to calculate the incidence rate ratio and 95% confidence intervals of outcomes according to the target and comparator vaccinations. Following reviewers’ suggestions, we also estimated incidence rate difference and 28 day absolute risk differences for associations with a significant calibrated incidence rate ratio.

We used empirical calibration to account for residual systematic error due to potential unobserved confounding.^32^ ^33^ To perform calibration, we first derived an empirical null distribution from the actual effect estimates for the negative control outcomes. We then used the null distribution to compute the calibrated P value and confidence intervals. This approach has been used in many previous studies in different clinical areas, including covid-19 repurposed treatments,^34^ ^35^ ^36^ and was acknowledged in the latest version of the ENCePP guide on methodological standards in pharmacoepidemiology.^37^ We only presented estimates specific to databases where empirical calibrations were conducted.

Finally, we conducted random effect meta-analysis to pool results across databases. Estimates from combinations of database, target, and comparator that passed the covariate balance diagnostic were included, regardless of the diagnostics on power or systematic error. Empirical calibration was conducted for meta-analysis as well. 

We stratified all analyses by age (10 year bands) and sex as prespecified in the study protocol. Only groups with sufficient power (minimum detectable rate ratio <5) were reported. All analyses were prespecified in a registered study protocol (https://www.encepp.eu/standards_and_guidances/methodologicalGuide.shtml), and conducted in R 3.6.0 using the open source OHDSI (observational health data science and informatics) tool stack. The Cyclops and EvidenceSynthesis packages are available via CRAN. All our analytical code is available for review in a dedicated Github repository (https://github.com/oxford-pharmacoepi/ROC22_CovVaxComparativeSafety/tree/main/CovVaxComparativeSafety).

### Patient and public involvement

Owing to the nature of this study and data privacy constraints, no patients or members of the public were involved in the study design, analysis, interpretation of data, or revision of the manuscript.

## Results

We identified 4.6 million people vaccinated with a first dose of ChAdOx1-S (3 789 631 UK CPRD, 606 399 Spain SIDIAP, 98 562 Germany DA, 27 698 France LPD, and 71 083 the Netherlands IPCI) and 1.6 million people vaccinated with a second dose of ChAdOx1-S (1 195 626 UK CPRD, 307 344 Spain SIDIAP, 31 200 Germany DA, 15 067 France LPD, and 38 884 the Netherlands IPCI) from all participating databases. We identified 1.1 million people vaccinated with single dose Ad26.COV2.S in three databases (37 723 Germany DA, 138 351 Spain SIDIAP, and 939 748 US Open Claims). We identified 10.6 million people vaccinated with a first dose of BNT162b2 (1 840 240 UK CPRD, 391 063 Germany DA, 6 055 754 US Open Claims, and 2 027 950 Spain SIDIAP), and 7.7 million people vaccinated with a second dose (1 369 238 UK CPRD, 321 099 Germany DA, 4 450 735 US Open Claims, and 1 357 509 Spain SIDIAP). We identified 4 261 016 people vaccinated with a first dose of mRNA-1273 in US Open Claims, and 2 938 023 people vaccinated with a second dose in US Open Claims. Cohort characteristics are summarised in supplementary A tables 2-7.

Noticeable differences existed in baseline patient characteristics before matching when comparing first dose ChAdOx1-S with first dose BNT162b2 recipients in UK CPRD data (supplementary A table 2). BNT162b2 recipients were more likely to be female (1 050 372 (58.2%) *v* 1 926 800 (51.5%)) and older and had a higher prevalence of comorbidities of interest. They were also more likely to use common medications such as treatments for hypertension and diabetes.

**Fig 1:**
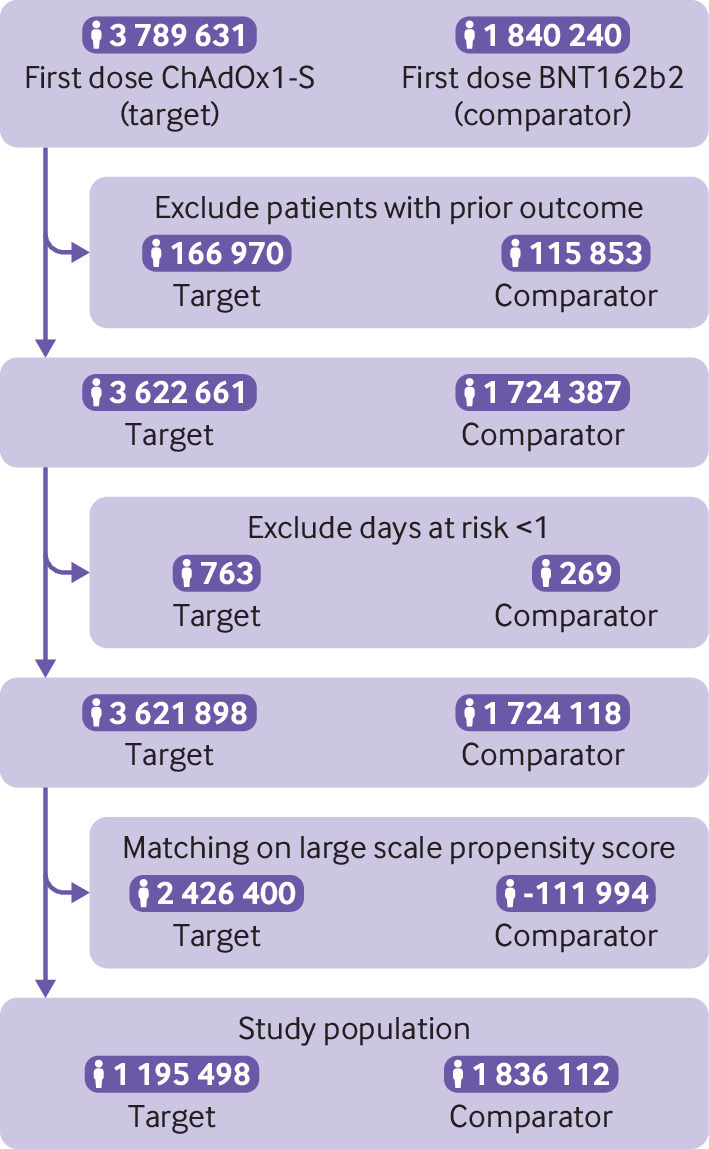
Study cohort selection. Example shows data from the UK database Clinical Practice Research Datalink Aurum used to compare the risk of thrombocytopenia after a first dose of ChAdOx1-S vaccine (target) compared with a first dose of BNT162b2 vaccine (comparator)

To reduce confounding, we estimated propensity scores for each vaccine pair and database. Supplementary A table 15 summarises the top 10 variables with stronger association with vaccine type in each of the databases. Propensity score matching led to a final cohort of 1.2 million ChAdOx1-S and 1.8 million BNT162b2 recipients (fig 1, supplementary A table 2). Patient characteristics after matching were comparable for most vaccination cohort pairs and databases, and are described in detail in supplementary A tables 2-7. The cohort selection process of all included cohorts is detailed in supplementary A table 14.

### Study diagnostics: confounding and statistical power

We applied three diagnostic tests to evaluate the robustness of our analyses, based on measured confounding, statistical power, and unmeasured confounding. Supplementary A table 8 summarises the diagnostics. Firstly, to avoid bias due to confounding, we did not analyse cohorts with substantial differences after matching: 14 analyses passed this diagnostic, where no patient characteristic had a standardised mean difference of ≥0.1 after propensity score matching. All available comparisons in UK CPRD, the Netherlands IPCI, and US Open Claims met the measured confounding requirements. In Spain SIDIAP, only Ad26.COV2.S compared with BNT162b2 showed covariate balance after matching. Other combinations of database, target, and comparator that passed the covariate balance test included: first and second dose Germany DA ChAdOx1-S compared with BNT162b2, Germany DA Ad26.COV2.S compared with BNT162b2, France LPD ChAdOx1-S compared with first dose BNT162b2, and France LPD ChAdOx1-S compared with first dose mRNA-1273. Conversely, no analysis was conducted in the US Hospital CDM, because residual confounding was noted (standardised mean difference >0.1 for ≥1 variables).

Secondly, eight analyses had sufficient statistical power for at least one outcome, as noted by a minimum detectable rate ratio <5. However, France LPD failed the power diagnostics for all study outcomes. Therefore, no database specific estimates were reported for France LPD, although this database contributed to meta-analyses (see below).

**Fig 2:**
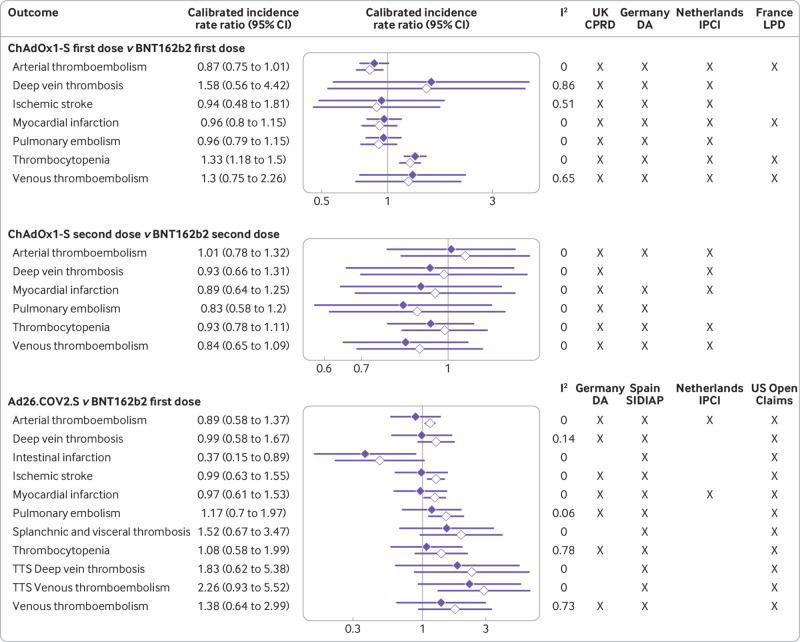
Meta-analytical estimates of incidence rate ratios of developing thrombosis with thrombocytopenia syndrome or venous or arterial thromboembolic events in the 28 days after covid-19 vaccination, according to information from routinely collected health databases. Lines with solid diamonds=calibrated estimates; lines with clear diamonds=uncalibrated estimates; TTS=thrombosis with thrombocytopenia syndrome; UK CPRD=Clinical Practice Research Datalink Aurum; Germany DA=IQVIA Disease Analyser Germany; Netherlands IPCI=Integrated Primary Care Information; France LPD=IQVIA Longitudinal Patient Data

Thirdly, negative control outcomes were used to identify residual confounding. Of seven combinations of database, target, and comparator with sufficient negative control outcomes, three had >20% associated with vaccine use (Germany DA Ad26.COV2.S *v* BNT162b2, US Open Claims Ad26.COV2.S *v* BNT162b2, and US Open Claims Ad26.COV2.S *v* mRNA-1273), suggesting the presence of substantial systematic error (supplementary A figs 1 and 2). Most of the estimates for these negative control outcomes had an incidence rate ratio >1, suggesting that our uncalibrated results overestimated risks, and that only calibrated results should be considered adequate.

For Germany DA ChAdOx1-S compared with second dose BNT162b2, France DA ChAdOx1-S compared with first dose mRNA-1273, and all comparisons within the Netherlands IPCI, too few negative control outcomes were observed, which precluded the use of empirical calibration.

### Comparative safety

Crude incidence rates before matching are available in supplementary A tables 9 and 10. Database specific results from the seven combinations that passed all three diagnostics after matching are reported in table 2 and table 3. Figure 2 depicts meta-analytical incidence rate ratios for all analyses where two or more databases contributed after diagnostics for three comparisons: first and second dose ChAdOx1-S compared with BNT162b2, and Ad26.COV2.S compared with BNT162b2.

**Table 2:** Incidence rates per 1000 person years and incidence rate ratios of developing thrombosis with thrombocytopenia syndrome or venous or arterial thromboembolic events in the 28 days after use of ChAdOx1-S versus mRNA based covid-19 vaccines in analyses passing diagnostic tests among matched cohorts

Vaccination and outcome	Database	No of participants after propensity score matching*	No of person years	No of events	Incidence rates (95% CI)/1000 person years	Calibrated incidence rate ratio (95% CI)
**First dose ChAdOx1-S *v* BNT162b2 **						
Arterial thromboembolism	UK CPRD	1 227 495	92 807	331	3.57 (3.19 to 3.97)	Reference
		1 886 308	140 256	416	2.97 (2.69 to 3.27)	0.85 (0.73 to 0.99)
	Germany DA	204 702	15 530	44	2.83 (2.06 to 3.8)	Reference
		82 643	6261	19	3.03 (1.83 to 4.74)	0.76 (0.41 to 1.39)
Deep vein thrombosis	UK CPRD	1 247 556	94 341	150	1.59 (1.35 to 1.87)	Reference
		1 912 752	142 268	193	1.36 (1.17 to 1.56)	0.89 (0.71 to 1.11)
	Germany DA	211 587	16 056	21	1.31 (0.81 to 2)	Reference
		85 163	6,454	21	3.25 (2.01 to 4.97)	2.62 (1.34 to 5.13)
Intestinal infarction	UK CPRD	1 270 917	96 126	14	0.15 (0.08 to 0.24)	Reference
		1 945 248	144 743	22	0.15 (0.1 to 0.23)	1.06 (0.53 to 2.13)
Ischaemic stroke	UK CPRD	1 264 894	95 666	76	0.79 (0.63 to 0.99)	Reference
		1 936 816	144 104	75	0.52 (0.41 to 0.65)	0.66 (0.48 to 0.92)
	Germany DA	210 616	15 982	15	0.94 (0.53 to 1.55)	Reference
		84 835	6429	11	1.71 (0.85 to 3.06)	1.34 (0.58 to 3.09)
Myocardial infarction	UK CPRD	1 233 874	93 294	201	2.15 (1.87 to 2.47)	Reference
		1 895 358	140 942	283	2.01 (1.78 to 2.26)	0.94 (0.78 to 1.14)
	Germany DA	208 975	15 856	26	1.64 (1.07 to 2.4)	Reference
		84 048	6368	10	1.57 (0.75 to 2.89)	0.70 (0.31 to 1.57)
Pulmonary embolism	UK CPRD	1 254 781	94 894	197	2.08 (1.8 to 2.39)	Reference
		1 922 818	143 038	269	1.88 (1.66 to 2.12)	0.93 (0.77 to 1.12)
	Germany DA	212 362	16 115	20	1.24 (0.76 to 1.92)	Reference
		85 493	6479	6	0.93 (0.34 to 2.02)	0.69 (0.26 to 1.83)
Thrombocytopenia	UK CPRD	1 195 498	90 381	442	4.89 (4.45 to 5.37)	Reference
		1 836 112	136 523	827	6.06 (5.65 to 6.48)	1.31 (1.16 to 1.49)
	Germany DA	204 508	15 516	78	5.03 (3.97 to 6.27)	Reference
		82 281	6234	35	5.61 (3.91 to 7.81)	1.01 (0.63 to 1.62)
Any thrombosis (venous thromboembolism or arterial thromboembolism) with thrombocytopenia syndrome	UK CPRD	1 263 613	95 571	64	0.67 (0.52 to 0.86)	Reference
		1 934 651	143 950	121	0.84 (0.7 to 1)	1.29 (0.94 to 1.77)
Venous thromboembolism	UK CPRD	1 233 788	93 290	314	3.37 (3 to 3.76)	Reference
		1 893 469	140 803	420	2.98 (2.7 to 3.28)	0.91 (0.78 to 1.06)
	Germany DA	209 244	15 878	40	2.52 (1.8 to 3.43)	Reference
		84 436	6398	25	3.91 (2.53 to 5.77)	1.61 (0.92 to 2.83)
**Second dose ChAdOx1-S *v* BNT162b2**						
Thrombocytopenia	UK CPRD	1 012 563	60 302	347	5.75 (5.16 to 6.39)	Reference
		747 810	38 474	230	5.98 (5.23 to 6.8)	0.94 (0.76 to 1.16)
Any thrombosis (venous thromboembolism or arterial thromboembolism) with thrombocytopenia syndrome	UK CPRD	1 076 722	64 277	42	0.65 (0.47 to 0.88)	Reference
		795 629	41 080	38	0.93 (0.65 to 1.27)	1.16 (0.71 to 1.89)
Deep vein thrombosis	UK CPRD	1 063 064	63 456	96	1.51 (1.23 to 1.85)	Reference
		784 878	40 506	61	1.51 (1.15 to 1.93)	0.93 (0.65 to 1.34)
Pulmonary embolism	UK CPRD	1 069 375	63 835	92	1.44 (1.16 to 1.77)	Reference
		789 797	40 767	53	1.3 (0.97 to 1.7)	0.86 (0.58 to 1.26)
Venous thromboembolism	UK CPRD	1 050 916	62 715	179	2.85 (2.45 to 3.3)	Reference
		775 486	39 998	105	2.63 (2.15 to 3.18)	0.87 (0.66 to 1.16)
Ischaemic stroke	UK CPRD	1 078 360	64 368	28	0.43 (0.29 to 0.63)	Reference
		796 695	41 129	23	0.56 (0.35 to 0.84)	1.20 (0.66 to 2.18)
Myocardial infarction	UK CPRD	1 050 018	62 656	109	1.74 (1.43 to 2.1)	Reference
		774 713	39 952	61	1.53 (1.17 to 1.96)	0.91 (0.64 to 1.3)
Arterial thromboembolism	UK CPRD	1 044 491	62 307	153	2.46 (2.08 to 2.88)	Reference
		770 339	39 705	101	2.54 (2.07 to 3.09)	1.05 (0.78 to 1.4)

UK CPRD=Clinical Practice Research Datalink Aurum; Germany DA=IQVIA Disease Analyser Germany.

* Numbers of participants differ for each outcome, because patients with a previous history of a given outcome of interest were excluded before the propensity score matching for that analysis.

**Table 3:** Incidence rates per 1000 person years and incidence rate ratios of developing thrombosis with thrombocytopenia syndrome or venous or arterial thromboembolic events in the 28 days after use of Ad26.COV2.S versus mRNA based covid-19 vaccines in analyses passing diagnostic tests among matched cohorts

Vaccination and outcome	Database	No of participants after propensity score matching†	No of person years	No of events	Incidence rates (95% CI)/1000 person years	Calibrated incidence rate ratio (95% CI)
**Ad26.COV2.S *v* BNT162b2**						
Thrombocytopenia	Germany DA	65 217	4894	14	2.86 (1.56 to 4.8)	Reference
		17 933	1213	12	9.89 (5.11 to 17.28)	1.30 (0.57 to 2.93)*
	Spain SIDIAP	386 334	19 944	197	9.88 (8.55 to 11.36)	Reference
		106 217	5037	49	9.73 (7.2 to 12.86)	0.77 (0.55 to 1.08)
	US Open Claims	2 364 195	172 698	470	2.72 (2.48 to 2.98)	Reference
		628 293	46 997	170	3.62 (3.09 to 4.2)	1.03 (0.63 to 1.7)*
	US Open Claims	2 231 498	169 780	484	2.85 (2.6 to 3.12)	Reference
		628 459	47 007	170	3.62 (3.09 to 4.2)	0.88 (0.56 to 1.4)*
Venous thromboembolism with thrombocytopenia syndrome	US Open Claims	2 404 904	175 752	13	0.07 (0.04 to 0.13)	Reference
		639 269	47 828	11	0.23 (0.11 to 0.41)	2.45 (0.95 to 6.29)*
Any thrombosis (venous thromboembolism or arterial thromboembolism) with thrombocytopenia syndrome	US Open Claims	2 365 254	172 778	378	2.19 (1.97 to 2.42)	Reference
		628 571	47 019	146	3.11 (2.62 to 3.65)	1.11 (0.67 to 1.84)*
Deep vein thrombosis	Spain SIDIAP	421 532	22 028	33	1.5 (1.03 to 2.1)	Reference
		116 087	5582	10	1.79 (0.86 to 3.29)	0.94 (0.45 to 1.96)
	US Open Claims	2 363 428	172 627	347	2.01 (1.8 to 2.23)	Reference
		628 002	46 974	121	2.58 (2.14 to 3.08)	0.98 (0.59 to 1.63)*
Pulmonary embolism	Spain SIDIAP	422 330	22 072	14	0.63 (0.35 to 1.06)	Reference
		116 315	5593	5	0.89 (0.29 to 2.09)	1.06 (0.37 to 3.07)
	US Open Claims	2 380 869	173 941	250	1.44 (1.26 to 1.63)	Reference
		632 834	47 339	105	2.22 (1.81 to 2.69)	1.18 (0.7 to 1.98)*
Venous thromboembolism	Spain SIDIAP	420 502	21 960	42	1.91 (1.38 to 2.59)	Reference
		115 760	5562	14	2.52 (1.38 to 4.22)	1.03 (0.55 to 1.93)
	US Open Claims	2 348 419	171 499	506	2.95 (2.7 to 3.22)	Reference
		624 001	46 670	190	4.07 (3.51 to 4.69)	1.06 (0.64 to 1.74)*
Ischaemic stroke	Spain SIDIAP	417 793	21 749	61	2.8 (2.15 to 3.6)	Reference
		114 999	5,509	18	3.27 (1.94 to 5.16)	1.04 (0.59 to 1.81)
	US Open Claims	2 348 140	171 471	540	3.15 (2.89 to 3.43)	Reference
		623 396	46 622	193	4.14 (3.58 to 4.77)	1.02 (0.62 to 1.67)*
Myocardial infarction	Spain SIDIAP	418 734	21 822	38	1.74 (1.23 to 2.39)	Reference
		115 276	5528	10	1.81 (0.87 to 3.33)	0.81 (0.38 to 1.71)
	US Open Claims	2 356 142	172 074	472	2.74 (2.5 to 3)	Reference
		625 168	46 757	168	3.59 (3.07 to 4.18)	1.02 (0.62 to 1.68)*
Intestinal infarction	US Open Claims	2 401 293	175 480	53	0.3 (0.23 to 0.4)	Reference
		638 257	47 752	7	0.15 (0.06 to 0.3)	0.35 (0.14 to 0.87)
Arterial thromboembolism	Spain SIDIAP	413 039	21 426	119	5.55 (4.6 to 6.65)	Reference
		113 588	5421	34	6.27 (4.34 to 8.76)	0.93 (0.62 to 1.39)
	US Open Claims	2 304 844	168 208	2231	13.26 (12.72 to 13.83)	Reference
		610 895	45 673	720	15.76 (14.63 to 16.96)	0.92 (0.57 to 1.48)*
Splanchnic and visceral thrombosis	US Open Claims	2 404 366	175 711	19	0.11 (0.07 to 0.17)	Reference
		639 111	47 816	10	0.21 (0.1 to 0.38)	1.46 (0.59 to 3.61)*
**Ad26.COV2.S *v* mRNA-1273**						
Deep vein thrombosis with thrombocytopenia syndrome	US Open Claims	2 271 774	172 851	12	0.07 (0.04 to 0.12)	Reference
		639 496	47 843	6	0.13 (0.05 to 0.27)	1.35 (0.45 to 4.05)*
Venous thromboembolism with thrombocytopenia syndrome	US Open Claims	2 271 552	172 835	14	0.08 (0.04 to 0.14)	Reference
		639 432	47 838	11	0.23 (0.11 to 0.41)	1.92 (0.77 to 4.8)*
Any thrombosis (venous thromboembolism or arterial thromboembolism) with thrombocytopenia syndrome	US Open Claims	2 232 550	169 861	380	2.24 (2.02 to 2.47)	Reference
		628 737	47 028	146	3.1 (2.62 to 3.65)	0.97 (0.61 to 1.55)*
Deep vein thrombosis	US Open Claims	2 230 157	169 676	336	1.98 (1.77 to 2.2)	Reference
		628 164	46 983	121	2.58 (2.14 to 3.08)	0.92 (0.57 to 1.48)*
Pulmonary embolism	US Open Claims	2 247 746	171 017	227	1.33 (1.16 to 1.51)	Reference
		632 997	47 349	105	2.22 (1.81 to 2.68)	1.15 (0.71 to 1.87)*
Venous thromboembolism	US Open Claims	2 215 499	168 558	488	2.9 (2.64 to 3.16)	Reference
		624 163	46 679	190	4.07 (3.51 to 4.69)	0.99 (0.62 to 1.56)*
Ischaemic stroke	US Open Claims	2 214 613	168 485	533	3.16 (2.9 to 3.44)	Reference
		623 557	46 632	193	4.14 (3.58 to 4.77)	0.93 (0.59 to 1.47)*
Myocardial infarction	US Open Claims	2 222 711	169 104	513	3.03 (2.78 to 3.31)	Reference
		625 329	46 766	168	3.59 (3.07 to 4.18)	0.86 (0.54 to 1.36)*
Intestinal infarction	US Open Claims	2 267 972	172 560	54	0.31 (0.24 to 0.41)	Reference
		638 418	47 761	7	0.15 (0.06 to 0.3)	0.29 (0.12 to 0.73)*
Arterial thromboembolism	US Open Claims	2 171 445	165 188	2246	13.6 (13.04 to 14.17)	Reference
		611 054	45 682	720	15.76 (14.63 to 16.96)	0.83 (0.54 to 1.28)*
Splanchnic and visceral thrombosis	US Open Claims	2 271 071	172 798	17	0.1 (0.06 to 0.16)	Reference
		639 274	47 826	10	0.21 (0.1 to 0.38)	1.48 (0.60 to 3.65)*

Germany DA=IQVIA Disease Analyser Germany; Spain SIDIAP=Information System for Research in Primary Care.

* Did not pass the systematic error diagnostic test of >80% uncalibrated confidence intervals covering 1.

† Numbers of participants differ for each outcome, because patients with a previous history of that outcome were excluded before the propensity score matching for that analysis.

We observed a total of 862 thrombocytopenia events were in the matched first dose ChAdOx1-S recipients from Germany and the UK, and 520 events after a first dose of BNT162b2. Meta-analyses showed an increased risk of thrombocytopenia after first dose ChAdOx1-S compared with BNT162b2, with a pooled calibrated incidence rate ratio of 1.33 (95% confidence interval 1.18 to 1.50; fig 2), a calibrated incidence rate difference of 1.18 (0.57 to 1.8) per 1000 person years, and an absolute risk difference of 8.21 (3.59 to 12.82) per 100 000 recipients. In UK CPRD data, 827 and 442 thrombocytopenia events occurred after first dose ChAdOx1-S and BNT162b2, respectively. Incidence rates were 6.06 per 1000 person years (95% confidence interval 5.65 to 6.48) and 4.89 (4.45 to 5.37), respectively, with a calibrated incidence rate ratio of 1.31 (1.16 to 1.49). This finding was not replicated in the Germany DA data, where the calibrated incidence rate ratio was 1.01 (0.63 to 1.62). No differential risk of thrombocytopenia was seen after the second dose of ChAdOx1-S versus second dose BNT162b2 (meta-analytical calibrated incidence rate ratio 0.93 (0.78 to 1.11); fig 2). Similarly, no increased risk of thrombocytopenia was noted after Ad26.COV2.S compared with first dose BNT162b2 (meta-analytical calibrated incidence rate ratio 1.08 (0.58 to 1.99); fig 2).

For venous thromboembolism and deep vein thrombosis, the meta-analysis was unreliable because of heterogeneity (I^2^ values of 65% and 86%, respectively). No increased risk of venous thromboembolism was seen after the first dose of ChAdOx1-S versus BNT162b2 either in Germany DA (calibrated incidence rate ratio 1.61 (95% confidence interval 0.92 to 2.83)) or UK CPRD (0.91 (0.78 to 1.06); table 2). An increased risk of deep vein thrombosis was seen after first dose ChAdOx1-S compared with BNT162b2 in Germany DA (2.62, 1.34 to 5.13), but not replicated in UK CPRD data (0.89, 0.71 to 1.11; table 2). No increased risk of pulmonary embolism was seen in either database, with calibrated incidence rate ratio 0.93 (0.77 to 1.12) and 0.69 (0.26 to 1.83) in UK and German data respectively. No differential risks of venous thromboembolism, deep vein thrombosis, or pulmonary embolism were noted when comparing second dose ChAdOx1-S with BNT162b2 in pooled meta-analysis or database specific analyses (table 2, fig 2). In line with this, no association was seen between vaccination with Ad26.COV2.S and any venous thromboembolic event in database specific (table 3) or pooled meta-analysis (fig 2). Regarding rare thrombosis, the meta-analysis showed a lower risk of intestinal infarction for the single dose Ad26.COV2.S users compared with first dose BNT162b2, with a pooled calibrated incidence rate ratio of 0.37 (0.15 to 0.89), an incidence rate difference of −0.41 (−1.17 to 0.35) per 1000 person years, and an absolute risk difference of −3.34 (−9.77 to 3.09) per 100 000 vaccinations (fig 2). No other rare thrombotic events had differential risks between cohorts.

For composite arterial thromboembolism, the pooled calibrated incidence rate ratio for first dose ChAdOx1-S compared with first dose BNT162b2 was 0.87 (95% confidence interval 0.75 to 1.01; fig 2). The two reliable database specific analyses in table 2 showed consistent findings—the calibrated incidence rate ratio was 0.85 (0.73 to 0.99) in CPRD UK and 0.76 (0.41 to 1.39) in Germany DA. In line with this, no differences in risk of arterial thromboembolism, ischaemic stroke, or myocardial infarction were seen after second dose ChAdOx1-S versus two dose BNT162b2 or after Ad26.COV2.S versus first dose BNT162b2. Similar results were seen also for ischaemic stroke and myocardial infarction when analysed separately (table 3, fig 2).

Thrombosis with thrombocytopenia syndrome was very rare, and could only be analysed in UK data for ChAdOx1-S and in US and Spanish data for Ad26.COV2.S. A trend towards an increase in risk of thrombosis with thrombocytopenia syndrome was observed in UK CPRD after first dose ChAdOx1-S compared with first dose BNT162b2 (calibrated incidence rate ratio 1.29 (95% confidence interval 0.94 to 1.77)). The calibrated incidence rate ratio after second dose was 1.16 (0.71 to 1.89). For comparing Ad26.COV2.S with BNT162b2, meta-analyses were possible for venous thromboembolism with thrombocytopenia syndrome and deep vein thrombosis with thrombocytopenia syndrome. A similar association was seen for venous thromboembolism with thrombocytopenia syndrome in the meta-analysis of US and Spanish data (pooled calibrated incidence rate ratio 2.26 (0.93 to 5.52)) and, with much more uncertainty, for deep vein thrombosis with thrombocytopenia syndrome (1.83 (0.62 to 5.38); fig 2). Database specific estimates from US Open Claims were in line with the pooled results (table 3).

### Sensitivity and subgroup analyses

Sensitivity analyses restricting the time window for thrombosis with thrombocytopenia syndrome to five days or reducing the threshold of platelet count (to lower than 100 000 platelets per μL) found results consistent with the main analysis (table 4).

**Table 4:** Sensitivity analysis of incidence rates per 1000 person years and incidence rate ratios of developing thrombosis with thrombocytopenia syndrome or venous or arterial thromboembolic events in the 28 days after use of adenovirus versus mRNA based covid-19 vaccination in analyses passing diagnostics

Sensitivity analysis and medical records database	Target comparator combination	Event	No of individuals after propensity score matching	No of person years	No of events	Incidence rates (95% CI)/1000 person years	Calibrated incidence rate ratio (95% CI)
**Thrombocytopenia window to five days before/after thrombosis after vaccination**							
UK CPRD	First dose BNT162b2 (comparator)	Any thrombosis (venous thromboembolism or arterial thromboembolism) with thrombocytopenia syndrome	1 934 829	95 580	63	0.66 (0.51 to 0.84)	Reference
	First dose ChAdOx1-S (target)		1 934 829	143 963	120	0.83 (0.69 to 1)	1.3 (0.95 to 1.79)
	Second dose BNT162b2 (comparator)		1 076 870	64 286	38	0.59 (0.42 to 0.81)	Reference
	Second dose ChAdOx1-S (target)		795 723	41 085	37	0.9 (0.63 to 1.24)	1.23 (0.74 to 2.04)
US Open Claims	First dose BNT162b2 (comparator)		2 365 342	172 785	376	2.18 (1.96 to 2.41)	Reference
	First dose Ad26.COV2.S (target)		628 592	47 020	143	3.04 (2.56 to 3.58)	1.09 (0.66 to 1.81)*
	First dose mRNA-1273 (comparator)		2 232 627	169 867	378	2.23 (2.01 to 2.46)	Reference
	Ad26.COV2.S (target)		628 758	47 030	143	3.04 (2.56 to 3.58)	0.96 (0.6 to 1.53)*
**Thrombocytopenia threshold of <100 000 platelets per microlitre**							
US Open Claims	First dose mRNA-1273 (comparator)	Deep vein thrombosis with thrombocytopenia syndrome	2 271 774	172 851	12	0.07 (0.04 to 0.12)	Reference
	Ad26.COV2.S (target)		639 496	47 843	6	0.13 (0.05 to 0.27)	1.35 (0.45 to 4.05)*
US Open Claims	First dose BNT162b2 (comparator)	Venous thromboembolism with thrombocytopenia syndrome	2 404 904	175 752	13	0.07 (0.04 to 0.13)	Reference
	Ad26.COV2.S (target)		639 269	47 828	11	0.23 (0.11 to 0.41)	2.45 (0.95 to 6.29)*
	First dose mRNA-1273 (comparator)		2 271 552	172 835	14	0.08 (0.04 to 0.14)	Reference
	Ad26.COV2.S (target)		639 432	47 838	11	0.23 (0.11 to 0.41)	1.92 (0.77 to 4.8)*
UK CPRD	First dose BNT162b2 (comparator)	Any thrombosis (venous thromboembolism or arterial thromboembolism) with thrombocytopenia syndrome	1 263 960	95 597	63	0.66 (0.51 to 0.84)	Reference
	First dose ChAdOx1-S (target)		1 935 138	143 986	119	0.83 (0.68 to 0.99)	1.29 (0.94 to 1.78)
	Second dose BNT162b2 (comparator)		1 077 077	64 299	39	0.61 (0.43 to 0.83)	Reference
	Second dose ChAdOx1-S (target)		795 893	41 094	37	0.9 (0.63 to 1.24)	1.25 (0.76 to 2.06)
US Open Claims	First dose BNT162b2 (comparator)		2 365 254	172 778	378	2.19 (1.97 to 2.42)	Reference
	Ad26.COV2.S (target)		628 571	47 019	146	3.11 (2.62 to 3.65)	1.11 (0.67 to 1.84)*
	First dose mRNA-1273 (comparator)		2 232 550	169 861	380	2.24 (2.02 to 2.47)	Reference
	Ad26.COV2.S (target)		628 737	47 028	146	3.1 (2.62 to 3.65)	0.97 (0.61 to 1.55)*

UK CPRD=Clinical Practice Research Datalink Aurum.

* Did not pass the systematic error diagnostics of >80% uncalibrated confidence intervals covering 1.

Stratified analyses are reported in supplementary A tables 11 and 12, and include findings from the UK CPRD and US Open Claims databases, as these were the only ones with sufficient power (minimum detectable rate ratio <5) for at least one outcome. An increased risk of thrombocytopenia was observed in those aged 40-49 years, 70-79 years, and among women in the UK data receiving first dose ChAdOx1-S compared with first dose BNT162b2. Additionally, the calibrated incidence rate ratio for composite arterial thromboembolism after ChAdOx1-S compared with BNT162b2 vaccination was lower in men, with a calibrated incidence rate ratio of 0.75 (95% confidence interval 0.61 to 0.92) (supplementary A table 11). Conversely, a subgroup analysis in US Open Claims data found an increased risk of arterial thromboembolism after Ad26.COV2.S compared with BNT162b2 and mRNA-1273 vaccination in people aged 20-29 years (calibrated incidence rate ratio 4.64 (2.16 to 9.97) and 5.10 (1.71 to 15.19), respectively). This finding was not replicated in any other subgroups.

## Discussion

### Principal findings

To our knowledge, this is the first multinational analysis of the comparative safety of adenovirus based compared with mRNA based covid-19 vaccines. In this matched cohort study, we compared the rates of thrombosis and of thrombosis with thrombocytopenia within 28 days after vaccination. We used routinely collected health data from five European countries and the US, and produced risk estimates after applying methods to minimise confounding and systematic error. After excluding many analyses because of identified confounding or limited statistical power, we observed a 30% increased risk of thrombocytopenia following first dose ChAdOx1-S compared with first dose BNT162b2.

### Findings in context

Thrombosis with concomitant thrombocytopenia was very rare, and we did not find any statistically significant increase in risk with either adenovirus based vaccine compared with any mRNA based vaccine. However, this finding should be put in context with previous research, because some of our estimates were close to significance, suggesting a potential increased risk of venous thromboembolism with thrombocytopenia syndrome after vaccination with Ad26.COV2.S. While thrombosis events and thrombocytopenia have been studied as separate outcomes, thrombosis with thrombocytopenia syndrome has rarely been studied as an individual outcome in previous real world studies owing to the complexity of the case definition and rare nature of the outcome in case definition.^38^ A US case series using the Vaccine Adverse Event Reporting System estimated rates of thrombosis with thrombocytopenia syndrome to be 3.83 per 1 million vaccine doses of Ad26.COV2.S and 0.00855 per 1 million vaccine doses of mRNA based covid-19 vaccines.^39^ Yet the authors stated that cases of thrombosis with thrombocytopenia syndrome reported after mRNA vaccines were associated with different demographic characteristics and medical history compared with cases after Ad26.COV2.S. By comparison, we used routinely collected health data and were able to estimate the comparative risks between vaccines, therefore minimising surveillance bias.

Subgroup analyses showed a 25% lower risk of arterial thromboembolism after first dose ChAdOx1-S versus BNT162b2 in men based in the UK, and a fourfold to fivefold increased risk of arterial thromboembolism in younger people (aged 20-29 years) vaccinated with Ad26.COV2.S compared with either mRNA vaccine in the US. However, these findings were not replicated in other contributing data sources or in other age groups, and deserve further research.

Thrombosis with thrombocytopenia syndrome or vaccine induced immune thrombotic thrombocytopenia was first reported after use of the ChAdOx1-S vaccine in early 2021.^4^ ^5^ A disproportionality analysis using WHO’s VigiBase database reported a safety signal for cerebral venous sinus thrombosis and ischaemic stroke for ChAdOx1-S, BNT162b2, and mRNA-1273.^40^ The authors called for well designed comparative safety studies on adverse events of all three vaccines. A study based on Danish and Norwegian data also found higher than expected rates of venous thromboembolism, pulmonary embolism, and cerebral venous sinus thrombosis after vaccination compared with background rates.^10^ While these studies provided important insights into the incidence of adverse outcomes reported after vaccination, they failed to adjust for potential confounders including comorbidity, frailty, nursing home residence, or history of other risk factors for thrombosis or coagulopathy.

The risk of thrombocytopenia after covid-19 vaccination has been studied by comparing vaccinated with unvaccinated groups, and using self-controlled designs. Hippisley-Cox et al conducted a self-controlled case series analysis of English data including about 30 million vaccinated people.^12^ They provided epidemiological evidence of a 30% increased risk of thrombocytopenia and venous thromboembolism after ChAdOx1-S vaccination, and an elevated risk of cerebral venous sinus thrombosis after ChAdOx1-S and BNT162b2. In a population based cohort study in England, Whiteley et al reported increased rates of thrombocytopenia during the 28 days after ChAdOx1-S compared with unvaccinated people among those aged under 70 years, but no association with BNT162b2.^41^ Our study compares both vaccines, and we found a 30% excess risk of thrombocytopenia after ChAdOx1-S compared with BNT162b2, consistent with previous studies.

Regarding arterial thromboembolism, a study from Scotland found an increased risk of arterial thromboembolic events in nested case-control analyses, which was attenuated in self-controlled case series analyses.^13^ An English self-controlled case series study found an increased risk of arterial thromboembolism after BNT162b2 but not ChAdOx1-S vaccination.^12^ Whiteley et al reported lower rates of major arterial thromboembolism and venous thromboembolism after vaccination with both ChAdOx1-S and BNT162b2 compared with unvaccinated people, after adjusting for potential confounding factors.^41^ Partially consistent with these results, we observed a lower rate of arterial thromboembolism after ChAdOx1-S compared with BNT162b2 in UK CPRD data, not replicated elsewhere or with other adenovirus based vaccines (Ad26.COV2.S *v* BNT162b2). The observed increase in risk of arterial thromboembolism in young people after Ad26.COV2.S versus mRNA based vaccines in US data was not replicated elsewhere or with ChAdOx1-S, and needs further research.

Study outcomes of cerebral venous sinus thrombosis and splanchnic and visceral thrombosis were also very rare. Kerr et al reported that cerebral venous sinus thrombosis was observed in about 16.34 per million doses of ChAdOx1-S, and 12.60 per million doses of BNT162b2. In a self-controlled case series analysis using data from England, Scotland, and Wales, ChAdOx1-S was associated with an elevated risk of cerebral venous sinus thrombosis in the 28 days after ChAdOx1-S vaccination (incidence rate ratio 1.93 (95% confidence interval 1.20 to 3.11)) but not after BNT162b2 vaccination.^11^ Similarly, a large record linkage study of hospital admissions in England showed an increased risk of cerebral venous sinus thrombosis after first dose ChAdOx1-S, seen only in adults aged under 65 years, and not after BNT162b2.^42^ In a previous study, we reported that background incidence rates varied across data sources, and suggested the use of analyses within databases for historical rate comparisons.^43^ In the present study, while we did not see large heterogeneity of incidence rates after vaccination between databases, relative rates varied. In our meta-analysis, the pooled estimates were largely driven by databases with larger sample sizes such as UK CPRD and US Open Claims data.

### Strengths and limitations

The results of our study should be interpreted in the context of its known limitations. Owing to heterogeneity across data sources, misclassification of vaccine use and outcomes might be problematic. Regarding vaccination, the UK and Spanish data sources captured vaccine information more reliably than previous studies through linkage to official vaccination registries. By contrast, the German and French records and US datasets are expected to include incomplete vaccine records. The use of comparative safety analyses minimises the impact of this problem, because only vaccinated cohorts are included for analysis.

Information bias due to outcome ascertainment was likely to be present in our study. We used robust methods for the creation and transportation of algorithms for the identification of all of the study events.^25^ However, some study events typically treated in hospital could be incompletely captured in some of our databases, including the German and French data sources. But inpatient data were available for the Spanish database through linkage and for US claims based on reimbursement. Our choice of matched cohort design should additionally minimise the impact of misclassification, because we do not expect incompleteness to be conditional on the vaccine received.

As in any observational study, analyses are susceptible to unmeasured confounders. Although the routinely collected health data and the use of large scale propensity scores allowed us to control for many potential confounders, we observed evidence of systematic errors in some analyses, especially in the US Open Claims database. Factors such as health seeking behaviour or family history of study outcomes were unmeasured or partially measured. In our study, we used empirical calibration to account for the unmeasured confounding.

Each country has its own immunisation schedule, and the studied vaccines were not all approved at the same time. For example, the vaccination campaign began on 8 December 2020 in England, and BNT162b2 was first given to care home residents, people aged ≥80 years, and frontline health workers, followed by vulnerable people and those aged ≥70 years. Individuals vaccinated earlier therefore have higher background rates, especially for thromboembolic events. Age and calendar time were therefore essential confounders, accounted for in our propensity score models. Propensity score matching created comparable cohorts, at the cost of excluding those with extreme propensity score values, who could not find a match. For example, in the UK CPRD, while 11% of the original BNT162b2 cohort was indexed in December 2020, almost none was included after matching. This factor should be taken into account when interpreting our findings.

We analysed data up to mid-2021, so only the first and second waves of the pandemic were represented. However, the proportion of included people with a history of covid-19 infection before vaccination was balanced in all eligible comparisons, both before and after matching.

In our study, we reported the database specific incidence rates of outcomes for both the original full cohorts and the propensity score matched cohorts. The incidence rates from the full cohorts were crude without any adjustment. While reflecting the real world incidence, they were highly subjected to the population characteristics and thus were not directly comparable between cohorts. The incidence rates from matched cohorts, on the other hand, can be compared since the propensity score matching accounted for the measured confounding. Caution is needed when interpreting these incidence rates as the generalisability of the rates is limited.

Finally, and despite the use of large international data sources, we had limited power for the analysis of thrombosis with thrombocytopenia syndrome, a rare event, resulting in only three databases (UK, Spain, and the US) contributing to our findings. In addition, meta-analysis was only meaningful for the analysis of Ad26.CoV.S, and resulted in wide confidence intervals and borderline (not significant) estimates. These analyses therefore warrant replication elsewhere.

Our study also has important strengths. While other epidemiological methods have been used in vaccine safety studies, a cohort study with active comparators enabled us to directly estimate the relative risk of developing thromboembolic events or thrombosis with thrombocytopenia syndrome after different covid-19 vaccines, which is not feasible in self-controlled designs or in observed to expected analyses. Our study therefore answers a more reliable question at this stage of the pandemic (ie, “which vaccine is safer” rather than “are vaccines safer than no vaccination”). The OMOP CDM allowed us to replicate the exact same analysis across different databases, therefore improving robustness, transparency, and reproducibility.

To reduce bias and confounding and ensure the reported results are reliable, we used robust diagnostics in our study design and statistical analysis plan. We used large scale propensity score modelling based on an L1 regularised logistic regression to minimise observed confounding. This approach has been shown to preferable to traditional propensity score estimation.^44^ We examined residual confounding after matching, and did not perform analyses where relevant confounding was observed. Further, we leveraged previously validated negative control outcomes 27 45 to assess risk of residual (unobserved) bias. Empirical calibration was then used to minimise any remaining systematic error.

**What is already known on this topic**

Thrombosis with thrombocytopenia syndrome is being investigated as an adverse reaction of adenovirus based covid-19 vaccines
The comparative risk of thrombosis with thrombocytopenia syndrome or thromboembolic events after vaccination with different covid-19 vaccines remains unclear

**What this study adds**

This multinational analysis of comparative safety of covid-19 vaccines used routinely collected data from Europe and the US
A 30% increased risk of thrombocytopenia was seen after first dose ChAdOx1-S compared with first dose BNT162b2 vaccination
A trend towards an increased risk of venous thrombosis with thrombocytopenia was observed after a first vaccine dose of Ad26.COV2.S, which needs replication elsewhere
Although rare, the observed risks after adenovirus based vaccines should be considered when planning further immunisation campaigns and future vaccine development

## Data Availability

Patient level data cannot be shared without approval from data custodians owing to local information governance and data protection regulations. The analytical code is available at: https://github.com/oxford-pharmacoepi/ROC22_CovVaxComparativeSafety/tree/main/CovVaxComparativeSafety. Additional correspondence and requests for materials should be addressed to the corresponding author (EB).

## Web extra

Extra material supplied by authors

Web appendix 1: Supplementary materials A

Web appendix 2: Supplementary materials B

## Notes

Contributors: XL, VS, and DP-A designed the study, and KV and CC reviewed and approved the study protocol. XL, VS, and DP-A wrote the related analysis plan, and KV and CC reviewed and approved it. TD-S, CY, CR, AD, PR, MM, LHJ, M-AM, J-MR-A, EB, and XL curated or analysed the data. MAS and KL contributed to analytical coding and related software. DP-A, KV, and PR were responsible for funding applications and project management. XL and DP-A wrote the first draft of the current manuscript, and all coauthors provided feedback and approved the final version for submission. XL and DP-A are guarantors. The corresponding author attests that all listed authors meet authorship criteria and that no others meeting the criteria have been omitted.

The lead author (XL) affirms that this manuscript is an honest, accurate, and transparent account of the study being reported; that no important aspects of the study have been omitted; and that any discrepancies from the study as planned (and, if relevant, registered) have been explained.

Dissemination to participants and related patient and public communities: We will disseminate a lay summary of our findings through our Twitter and other social media accounts. Members of the general public could also help disseminate the paper, especially on social media.

Provenance and peer review: Not commissioned; externally peer reviewed.
