# Supplementary material for: Comparative risk of thrombosis with thrombocytopenia syndrome or thromboembolic events associated with different covid-19 vaccines: international network cohort study from five European countries and the US
Source: BMJ. 2022 Oct 26;379:e071594. doi: 10.1136/bmj-2022-071594 (PMC9597610; doi:10.1136/bmj-2022-071594)

Contents

Tables 2

Supplementary Table 1. Negative control outcome concept list. 2

Supplementary Table 2. Baseline characteristics of eligible ChAdOx1 and BNT162b2 cohorts of vaccinated people identified EU based databases. 3

Supplementary Table 3. Baseline characteristics of eligible Ad26.COV2.S and BNT162b2 cohorts of vaccinated people identified EU based databases. 5

Supplementary Table 4. Baseline characteristics of eligible Ad26.COV2.S, BNT162b2, and mRNA-1273 cohorts of vaccinated people identified US Open Claims. 8

Supplementary Table 5. Baseline characteristics of eligible cohorts of vaccinated people identified in IQVIA LPD France 9

Supplementary Table 6. Baseline characteristics of eligible cohorts of vaccinated people identified in IPCI 12

Supplementary Table 7. Baseline characteristics of eligible cohorts of vaccinated people identified in IQVIA US Hospital CDM 13

Supplementary Table 8. Summary of diagnostics at the database-target-comparator level 15

Supplementary Table 9. Crude incidence rate and corresponding 95% confidence intercals, European data 16

Supplementary Table 10. Crude incidence rate and corresponding 95% confidence intercals, US data 19

Supplementary Table 11. Incidence rates before- and after- propensity score matching. 22

Supplementary Table 12. Age and sex stratified incidence rate ratio, CPRD UK. 24

Supplementary Table 13. Age and sex stratified incidence rate ratio, US Open Claims 26

Supplementary Table 14. Cohort selection and minimal detectable relative risk, database-target-outcome. 30

Supplementary Table 15. List of covariates with top 10 highest absolute value of propensity score model coefficient. 34

Figures 37

Figure 1. Passed systematic error diagnostic: <20% negative control outcomes associated with exposure in the uncalibrated analyses (CPRD Aurum, 1st dose ChAdOx1 vs BNT162b2) 37

Figure 2. Systematic error in the comparison between Janssen and mRNA vaccines in US Open Claims 38

Figure 3. Systematic error in meta-analysis 39

Tables

Supplementary Table 1. Negative control outcome concept list.

Concept Id	Outcome Name		Concept Id	Outcome Name
438945	Accidental poisoning by benzodiazepine-based tranquilizer		138690	Infestation by Pediculus
434455	Acquired claw toes		4152376	Intentional self poisoning
316211	Acquired spondylolisthesis		192953	Intestinal adhesions with obstruction
201612	Alcoholic liver damage		196347	Intestinal parasitism
438730	Alkalosis		137977	Jaundice
441258	Anemia in neoplastic disease		317510	Leukemia
432513	Animal bite wound		765053	Lump in right breast
4171556	Ankle ulcer		378165	Nystagmus
4098292	Antiphospholipid syndrome		434085	Obstruction of duodenum
77650	Aseptic necrosis of bone		4147016	Open wound of buttock
4239873	Benign neoplasm of ciliary body		4129404	Open wound of upper arm
23731	Benign neoplasm of larynx		438120	Opioid dependence
199764	Benign neoplasm of ovary		75924	Osteodystrophy
195500	Benign neoplasm of uterus		432594	Osteomalacia
4145627	Biliary calculus		30365	Panhypopituitarism
4108471	Burn of digit of hand		4108371	Peripheral gangrene
75121	Burn of lower leg		440367	Plasmacytosis
4284982	Calculus of bile duct without obstruction		439233	Poisoning by antidiabetic agent
434327	Cannabis abuse		442149	Poisoning by bee sting
78497	Cellulitis and abscess of toe		4314086	Poisoning due to sting of ant
4001454	Cervical spine ankylosis		4147660	Postural kyphosis
4068241	Chronic instability of knee		434319	Premature ejaculation
195596	Chronic pancreatitis		199754	Primary malignant neoplasm of pancreas
4206338	Chronic salpingitis		4311499	Primary malignant neoplasm of respiratory tract
4058397	Claustrophobia		436635	Primary malignant neoplasm of sigmoid colon
74816	Contusion of toe		196044	Primary malignant neoplasm of stomach
73302	Curvature of spine		433716	Primary malignant neoplasm of testis
4151134	Cyst of pancreas		133424	Primary malignant neoplasm of thyroid gland
77638	Displacement of intervertebral disc without myelopathy		194997	Prostatitis
195864	Diverticulum of bladder		80286	Prosthetic joint loosening
201346	Edema of penis		443274	Psychostimulant dependence
200461	Endometriosis of uterus		314962	Raynaud’s disease
377877	Esotropia		37018294	Residual osteitis
193530	Follicular cyst of ovary		4288241	Salmonella enterica subspecies arizonae infection
4094822	Foreign body in respiratory tract		45757269	Sclerosing mesenteritis
443421	Gallbladder and bile duct calculi		74722	Secondary localized osteoarthrosis of pelvic region
135215	Hashimoto thyroiditis		200348	Secondary malignant neoplasm of large intestine
442190	Hemorrhage of colon		43020446	Sedative withdrawal
43020475	High risk heterosexual behavior		74194	Sprain of spinal ligament
194149	Hirschsprung’s disease		4194207	Tailor’s bunion
443204	Human ehrlichiosis		193521	Tropical sprue
4226238	Hyperosmolar coma due to diabetes mellitus		40482801	Type II diabetes mellitus uncontrolled

4032787	Hyperosmolarity		74719	Ulcer of foot
197032	Hyperplasia of prostate		196625	Viral hepatitis A without hepatic coma
140362	Hypoparathyroidism		197494	Viral hepatitis C
435371	Hypothermia		4284533	Vitamin D-dependent rickets

Supplementary Table 2. Baseline characteristics of eligible ChAdOx1 and BNT162b2 cohorts of vaccinated people identified EU based databases.

	**UK CPRD**						**Germany DA**					
	**Before PS matching**			**After PS matching**			**Before PS matching**			**After PS matching**		
	**ChAdOx1 1st dose**	**BNT162b2 1st dose**		**ChAdOx1 1st dose**	**BNT162b2 1st dose**		**ChAdOx1 1st dose**	**BNT162b2 1st dose**		**ChAdOx1 1st dose**	**BNT162b2 1st dose**	
**Characteristic**	**%**	**%**	**SMD**	**%**	**%**	**SMD**	**%**	**%**	**SMD**	**%**	**%**	**SMD**
**Cohort Count**	3,741,359	1,804,763		1,247,556	1,912,752		88,463	344,916		85,163	211,587	
**Age group**												
<20	0.4	0.6	-0.03	0.5	0.6	-0.01	0.5	2	-0.14	0.4	0.4	0.01
20 - 29	4.4	5.4	-0.05	4.7	4.7	0.00	4.3	7.4	-0.14	3.8	3.5	0.02
30 - 39	7.5	7.9	-0.02	8.3	6.9	0.05	5.9	9.9	-0.15	5.5	5.2	0.01
40 - 49	18.3	10.9	0.21	10.4	11.1	-0.03	8.7	12.8	-0.14	8.2	7.8	0.01
50 - 59	28.8	16	0.31	15.6	16.9	-0.03	19.1	22.4	-0.08	19	18.7	0.01
60 - 69	21.3	17.8	0.09	23.4	22.2	0.03	37.2	22.5	0.33	37.5	37.3	0.01
70 - 79	14.7	20.4	-0.15	27.2	25.8	0.03	18	16.1	0.05	18.8	19.9	-0.03
80 - 89	3.6	17.7	-0.47	8.8	9.5	-0.02	5.4	5.9	-0.02	5.7	6	-0.02
90 - 99	1.1	3.3	-0.15	2.1	2.1	0.00	1.1	1	0.01	1.1	1.2	-0.01
100 – 109	0.1	0.1	-0.01	0.1	0.1	0.01						
**Gender: female**	51.5	58.2	-0.14	56.1	56.1	0.00	41.2	55	-0.28	42	42.3	0.00
**Index month**												
12		11										
1	13.7	47.6	-0.79	37.9	41.6	-0.08						
2	29	36.3	-0.16	54.8	50.2	0.09						
3	45.7	3.6	1.12	4.4	5.2	-0.04	1.9	0.1	0.18	0.5	0.4	0.02
4	9	1.6	0.34	2.3	2.3	-0.01	22.2	30.7	-0.20	24.6	25.1	-0.01
5	2.5	0.4	0.17	0.6	0.6	-0.01	60.4	26.4	0.73	61.1	61.3	0.00
6							13.2	24.5	-0.29	12.1	11.9	0.01
7							2	12.7	-0.42	1.6	1.4	0.02
8							0.3	5.5	-0.32	0.1	0.1	0.00
**Covid-19 infection prior vaccination**	5.5	4	0.07	4.6	4.4	0.01						
**Charlson index – Romano adaptation**	79.8	131.1	-0.33	123.6	121.7	0.01	158.8	157.2	0.01	164.1	169.1	-0.02
**CHADS2VASc**	128.3	200.6	-0.54	190.4	187.3	0.02	196.9	194	0.02	204	209.1	-0.03

**Medical history: General**												
Acute respiratory disease	41.4	44.1	-0.05	44	43.9	0.00	57.9	59.6	-0.03	58.1	57.7	0.01
Attention deficit hyperactivity disorder	0.3	0.2	0.01	0.2	0.2	0.00	0.6	0.4	0.02	0.6	0.5	0.00
Chronic obstructive lung disease	3.1	5.3	-0.11	5.5	5.2	0.01	11.5	11.3	0.00	12	12.2	-0.01
Crohn’s disease	0.5	0.6	-0.01	0.7	0.6	0.01	0.6	0.8	-0.03	0.6	0.6	0.00
Dementia	1.2	2	-0.07	1.8	1.7	0.02	2.4	2.2	0.01	2.5	2.6	-0.01
Depressive disorder	21.3	20.3	0.02	21.3	21.2	0.00	22	24.2	-0.05	22.8	23	-0.01
Diabetes mellitus	10	15.5	-0.17	16.1	15.8	0.01	21.4	20.1	0.03	22.4	23.1	-0.02
Gastroesophageal reflux disease	5.1	5.9	-0.04	5.9	5.9	0.00	5.5	5.8	-0.01	5.8	5.8	0.00
Gastrointestinal hemorrhage	8.6	9.7	-0.04	9.7	9.5	0.01	3.6	3.2	0.02	3.6	3.5	0.00
Hyperlipidemia	9.2	13	-0.12	13.1	12.9	0.01	34.5	29.6	0.11	35.5	36.1	-0.01
Hypertensive disorder	23.8	35.8	-0.26	34.7	34.2	0.01	49.8	43.8	0.12	52.1	52.9	-0.02
Lesion of liver	0.9	1.2	-0.03	1.3	1.3	0.00	1.3	1.4	0.00	1.4	1.4	0.00
Obesity	4.7	5.3	-0.03	5.7	5.6	0.01	15.5	15.3	0.01	16	16.2	-0.01
Osteoarthritis	18.9	28	-0.21	26.9	26.5	0.01	33.1	29	0.09	33.9	34.2	-0.01
Pneumonia	2.8	3.6	-0.05	3.6	3.4	0.01	6.6	6.9	-0.01	6.7	6.8	-0.01
Psoriasis	4.4	4.7	-0.01	4.9	4.9	0.00	4.2	3.7	0.02	4.3	4.4	0.00
Renal impairment	6.3	13.3	-0.24	11.2	11.1	0.00	8.2	7.3	0.03	8.3	8.6	-0.01
Rheumatoid arthritis	1.1	1.6	-0.05	1.6	1.6	0.00	3.7	4	-0.01	3.9	4	-0.01
Ulcerative colitis	0.7	0.8	-0.01	0.9	0.9	0.01	0.8	1	-0.02	0.8	0.8	0.00
Urinary tract infectious disease	13.5	16.9	-0.10	16.1	16	0.00	15	17.3	-0.06	15.6	15.6	0.00
Viral hepatitis C	0.2	0.2	0.00	0.2	0.2	0.00	0.4	0.4	0.00	0.4	0.4	0.00
Visual system disorder	39.7	48.7	-0.18	47.4	46.9	0.01	26.4	25.4	0.02	26.8	27.1	-0.01
**Medical history: Cardiovascular disease**												
Atrial fibrillation	2.9	6.6	-0.17	5.5	5.4	0.00	3.3	3.3	0.00	3.5	3.7	-0.01
Cerebrovascular disease	2.8	5.5	-0.14	4.9	4.8	0.01	7.7	7.3	0.02	8.1	8.5	-0.02
Coronary arteriosclerosis	0.8	1.4	-0.07	1.4	1.3	0.00	5.7	5.4	0.01	6	6.2	-0.01
Heart disease	10.5	19.5	-0.25	17.7	17.5	0.01	31	29.4	0.04	32.3	33.3	-0.02
Heart failure	1.3	2.6	-0.10	2.2	2.2	0.01	7.3	6.8	0.02	7.5	7.9	-0.02
Ischemic heart disease	4.1	8.3	-0.17	7.6	7.4	0.01	13.8	12.7	0.03	14.5	14.9	-0.01
Peripheral vascular disease	0.7	1.3	-0.06	1.3	1.2	0.01	6.7	5.8	0.04	6.9	7.1	-0.01
Pulmonary embolism	1	1.5	-0.05	1.3	1.3	0.00	0.9	1.5	-0.05	0.8	0.9	0.00
Venous thrombosis	4.4	6.1	-0.08	4.2	4.1	0.01	5.1	5.7	-0.03	4	4.1	0.00
**Medical history: Neoplasms**												

Malignant tumor of breast	1.6	2.7	-0.07	2.7	2.7	0.00	1.7	2.3	-0.04	1.8	1.9	0.00
Hematologic neoplasm	0.4	0.7	-0.04	0.6	0.6	0.00	0.9	1.2	-0.03	1	1	-0.01
Malignant lymphoma	0.3	0.6	-0.04	0.6	0.6	0.00	0.7	0.9	-0.02	0.7	0.7	-0.01
Malignant neoplasm of anorectum	0.2	0.4	-0.04	0.4	0.4	0.00	0.4	0.4	0.00	0.4	0.5	-0.01
Malignant neoplastic disease	8.2	14.7	-0.21	13.4	13.4	0.00	12.1	12.8	-0.02	12.6	13	-0.01
Malignant tumor of colon	0.3	0.7	-0.05	0.6	0.6	0.00	0.8	0.8	0.00	0.8	0.9	-0.01
Malignant tumor of lung	0.2	0.3	-0.03	0.3	0.3	0.00	0.2	0.3	-0.02	0.2	0.3	0.00
**Medication use**												
Lipid modifying agents	9.4	15.7	-0.19	15.6	15.3	0.01	19.5	17.1	0.06	20.8	21.5	-0.02
Antithrombotic agents	4.4	8.9	-0.18	7.8	7.7	0.00	14.1	14.2	0.00	14.9	15.5	-0.02
Agents acting on the renin-angiotensin system	9.1	14	-0.16	13.7	13.6	0.00	35.9	30.6	0.11	38.3	39.1	-0.02
Beta blocking agents	4.5	7.7	-0.13	7.1	7.1	0.00	23.1	20.8	0.06	24.7	25.4	-0.02
Diuretics	3.4	6.2	-0.13	5.6	5.6	0.00	19.8	17.2	0.07	21.1	21.6	-0.01
Drugs for acid related disorders	11.1	15.3	-0.13	15	14.9	0.00	17.4	18.1	-0.02	18.6	19	-0.01
Calcium channel blockers	6.5	9.9	-0.12	9.8	9.7	0.00	16	13.7	0.07	17	17.3	-0.01
Antiinflammatory and antirheumatic products	6.5	9.5	-0.11	9.3	9.1	0.01	20.7	19	0.04	21.7	22	-0.01
Drugs used in diabetes	4.3	6.8	-0.11	7.1	7	0.01	11.4	10.4	0.03	12.1	12.5	-0.01
Drugs for obstructive airway diseases	9.5	11.9	-0.08	12.1	12	0.01	9.3	10.2	-0.03	9.9	10	-0.01
Antibacterials for systemic use	8.6	10.6	-0.07	10.5	10.3	0.01	6.4	7.9	-0.06	6.6	6.8	-0.01
Opioids	5.7	7.3	-0.06	7.3	7.2	0.00	4.9	4.8	0.01	5.1	5.2	-0.01
Antineoplastic agents	0.4	0.6	-0.03	0.6	0.6	0.00	0.9	1	-0.01	0.9	1	0.00
Immunosuppressants	0.4	0.6	-0.03	0.6	0.6	0.00	0.7	1	-0.03	0.8	0.8	0.00
Antiepileptics	2.5	2.9	-0.03	3	3	0.00	2.4	2.7	-0.02	2.6	2.7	-0.01
Psycholeptics	2.7	3.1	-0.02	3.2	3.2	0.00	4.3	4.6	-0.02	4.6	4.7	-0.01
Antipsoriatics	0.3	0.3	0.00	0.3	0.4	0.00	0.3	0.2	0.00	0.3	0.3	0.00

- Characteristics are based on deep vein thrombosis as the outcome event. Numbers are slightly different for other outcome events, as patients with prior history of that event were excluded from the analysis.

Supplementary Table 3. Baseline characteristics of eligible Ad26.COV2.S and BNT162b2 cohorts of vaccinated people identified EU based databases.

	**Germany DA**						**Spain SIDIAP**					
	**Before PS matching**			**After PS matching**			**Before PS matching**			**After PS matching**		
	**Ad26.COV2.S**	**BNT162b2**		**Ad26.COV2.S**	**BNT162b2**		**Ad26.COV2.S**	**BNT162b2**		**Ad26.COV2.S**	**BNT162b2**	
Characteristic	**%**	**%**	**SMD**	**%**	**%**	**SMD**	**%**	**%**	**SMD**	**%**	**%**	**SMD**

**Cohort Count**	18,485	344,916		18,428	67,016		126,189	1,973,028		116,087	421,532	
**Age group**												
<20							0.3	0.2	0.02	0.3	0.2	0.01
20 - 29	10.3	7.4	0.10	5	4.9	0.01	3	1.9	0.07	3	2.5	0.03
30 - 39	13.1	9.9	0.10	8.1	8	0.00	4.8	8.3	-0.14	4.8	5	-0.01
40 - 49	16.9	12.8	0.11	13.8	13.8	0.00	40.8	25.6	0.33	42.4	44.7	-0.05
50 - 59	28.1	22.4	0.13	29.3	29.5	-0.01	13.7	24.9	-0.29	14.9	14.2	0.02
60 - 69	21	22.5	-0.04	28.3	28.5	-0.01	18.8	2.7	0.54	12.5	10.4	0.07
70 - 79	6.1	16.1	-0.32	9.6	9.5	0.00	18.4	19.9	-0.04	21.7	22.7	-0.03
80 - 89	2.6	5.9	-0.16	4.5	4.5	0.00	0.2	12.8	-0.53	0.3	0.2	0.02
90 - 99	0.5	1	-0.05	0.9	0.8	0.01	0.1	3.7	-0.27	0.1	0.1	0.01
**Gender: female**	38.2	55	-0.34	41.6	41.2	0.01	46.1	54.1	-0.16	46.3	46.8	-0.01
**Index month**												
1							0.1	6.6	-0.37	0.1	0.1	-0.01
2							0.1	5.9	-0.35	0.1	0.1	-0.01
3		0.1					0.1	8.5		0.1	0.2	
4		30.7					9.4	18.8	-0.27	11.1	10.8	0.01
5	20.2	26.4	-0.15	24.1	23.7	0.01	16.6	22.4	-0.15	19.3	19.6	-0.01
6	63.1	24.5	0.84	65.8	66.4	-0.01	73.7	37.7	0.78	69.3	69.2	0.00
7	11	12.7	-0.05	7.7	7.4	0.01						
8	5.7	5.5	0.01	2.3	2.4	-0.01						
**Covid-19 infection prior vaccination**	1.7	2.1	-0.03	1.9	2	-0.01	8.2	6.6	0.06	7.4	7.7	-0.01
**Charlson index - Romano adaptation**	109.1	157.2	-0.24	157.3	158.5	-0.01	68.6	106.2	-0.22	69.7	70.5	-0.01
**CHADS2VASc**	132.1	194	-0.42	185.7	186.6	-0.01	123.4	185.5	-0.41	126.7	122.7	0.03
**Medical history: General**												
Acute respiratory disease	62.8	59.6	0.07	64.6	64.9	-0.01	53.8	56.2	-0.05	54.5	54.9	-0.01
Attention deficit hyperactivity disorder	0.6	0.4	0.02	0.7	0.7	0.00	0.3	0.2	0.02	0.3	0.3	0.00
Chronic liver disease	0.9	1	-0.01	1.3	1.3	0.00	1.8	1.4	0.03	1.7	1.8	0.00
Chronic obstructive lung disease	9.3	11.3	-0.07	12.8	13	-0.01	4.1	5.4	-0.06	4.1	3.9	0.01
Crohn's disease	0.6	0.8	-0.03	0.7	0.7	0.01	0.2	0.2	-0.01	0.2	0.2	-0.01
Dementia	1.4	2.2	-0.06	2.4	2.3	0.00	0.3	2.7	-0.19	0.4	0.4	0.00
Depressive disorder	22	24.2	-0.05	28.1	28.4	-0.01	13.7	15.2	-0.04	13.7	14	-0.01
Diabetes mellitus	15.8	20.1	-0.11	24.6	24.7	0.00	11.7	14.1	-0.07	11.6	11.5	0.00
Gastroesophageal reflux disease	4.5	5.8	-0.06	6.3	6.3	0.00	7	7.9	-0.04	7	6.9	0.00
Human immunodeficiency virus infection	0.2	0.3	-0.02	0.2	0.2	-0.01	0.6	0.3	0.05	0.6	0.6	0.00
Hypertensive disorder	36.2	43.8	-0.15	55.6	55.7	0.00	25.5	34.7	-0.20	25.6	25	0.01
Lesion of liver	1.1	1.4	-0.02	1.7	1.7	0.00	1.1	1	0.01	1.1	1.1	-0.01
Obesity	14.2	15.3	-0.03	19.3	19.4	0.00	24	25.5	-0.04	24	23.9	0.00
Osteoarthritis	26.1	29	-0.07	34.2	34.3	0.00	16.8	24.5	-0.19	17	16.3	0.02
Pneumonia	5.5	6.9	-0.06	7	6.8	0.01	6.6	8.1	-0.06	6.6	6.7	-0.01

Psoriasis	3.4	3.7	-0.02	4.4	4.4	0.00	2.8	3	-0.01	2.8	2.9	0.00
Renal impairment	4.6	7.3	-0.11	7.3	7.3	0.00	3.8	9.6	-0.24	3.9	3.8	0.00
Rheumatoid arthritis	2.6	4	-0.08	3.7	3.7	0.00	0.5	0.8	-0.04	0.5	0.6	-0.01
Schizophrenia	0.5	0.4	0.01	0.7	0.6	0.00	1.2	0.5	0.07	1.2	1.3	-0.01
Ulcerative colitis	0.7	1	-0.03	0.9	0.8	0.01	0.4	0.4	-0.01	0.4	0.4	-0.01
Urinary tract infectious disease	12.8	17.3	-0.13	15.9	15.7	0.01	19.3	24	-0.12	19.4	19.8	-0.01
Viral hepatitis C	0.6	0.4	0.03	0.8	0.7	0.01	1.3	0.9	0.04	1.3	1.3	0.00
**Medical history: Cardiovascular disease**												
Atrial fibrillation	1.7	3.3	-0.10	2.8	2.7	0.00	2.1	5.5	-0.18	2.2	2.2	0.00
Cerebrovascular disease	3.9	7.3	-0.15	6.5	6.6	0.00	1.8	3.6	-0.12	1.8	2	-0.01
Heart disease	21.3	29.4	-0.19	31.6	31.7	0.00	12.2	20.1	-0.22	12.4	12.4	0.00
Heart failure	3.9	6.8	-0.13	6.7	6.8	0.00	1.5	4.2	-0.16	1.6	1.6	-0.01
Peripheral vascular disease	4.1	5.8	-0.08	6.9	7	0.00	1.7	2.5	-0.06	1.7	1.7	-0.01
Pulmonary embolism	0.6	1.5	-0.09	0.8	0.8	0.00	0.4	0.7	-0.04	0.4	0.4	0.00
Venous thrombosis	4.2	5.7	-0.07	4.3	4.3	0.00	2.9	3.8	-0.05	2.9	2.8	0.00
**Medical history: Neoplasms**												
Malignant tumor of breast	0.9	2.3	-0.12	1.2	1.1	0.01	1.2	1.8	-0.05	1.3	1.3	0.00
Hematologic neoplasm	0.8	1.2	-0.04	1.1	1.1	0.00	0.6	1	-0.04	0.6	0.7	-0.01
Malignant lymphoma	0.4	0.9	-0.06	0.6	0.6	0.00	0.2	0.4	-0.04	0.2	0.2	0.00
Malignant neoplasm of anorectum	0.2	0.4	-0.04	0.3	0.3	0.00	0.2	0.4	-0.03	0.3	0.3	0.00
Malignant neoplastic disease	7.1	12.8	-0.19	10	10	0.00	7.1	11.9	-0.16	7.5	7.4	0.00
Malignant tumor of lung	0.1	0.3	-0.05	0.2	0.2	-0.01	0.2	0.2	-0.02	0.2	0.2	0.00
Malignant tumor of urinary bladder	0.2	0.4	-0.04	0.3	0.3	0.00	0.5	0.9	-0.05	0.6	0.6	0.00
**Medication use**												
Lipid modifying agents	10.9	17.1	-0.18	20.3	20.4	0.00	15.3	19.6	-0.11	15.5	15.4	0.00
Antithrombotic agents	8.1	14.2	-0.20	14.7	14.9	-0.01	9.6	16.3	-0.20	9.7	9.7	0.00
Agents acting on the renin-angiotensin system	23	30.6	-0.17	42.4	42.6	0.00	18.9	25.7	-0.17	19	18.6	0.01
Diuretics	11.5	17.2	-0.16	21.4	21.5	0.00	12	18.9	-0.19	12.2	12	0.01
Calcium channel blockers	10.1	13.7	-0.11	18.9	19	0.00	6	9.2	-0.12	6.1	5.9	0.01
Antiinflammatory and antirheumatic products	17.6	19	-0.03	26.8	27.1	-0.01	23.9	24.1	0.00	23.9	24.1	0.00
Drugs used in diabetes	7.5	10.4	-0.10	13.8	13.8	0.00	9	10.7	-0.06	8.9	8.9	0.00
Drugs for obstructive airway diseases	7.3	10.2	-0.10	11.4	11.5	0.00	12.6	14	-0.04	12.7	12.7	0.00
Antibacterials for systemic use	6.3	7.9	-0.06	8.6	8.8	-0.01	13.6	14.6	-0.03	13.5	13.7	0.00
Antineoplastic agents	0.5	1	-0.05	0.9	0.9	0.00	0.7	1	-0.04	0.7	0.8	-0.01
Immunosuppressants	0.4	1	-0.08	0.6	0.6	0.00	0.6	1	-0.04	0.7	0.9	-0.02
Antiepileptics	1.8	2.7	-0.06	3.2	3.3	-0.01	7.2	7.1	0.01	7.2	7.5	-0.01
Psycholeptics	3.3	4.6	-0.07	5.8	5.9	0.00	19.8	23.1	-0.08	19.9	20.2	-0.01
Antipsoriatics	0.2	0.2	-0.01	0.3	0.3	-0.01	0.9	1	-0.02	0.9	0.9	0.00
Antidepressants	5.1	6.5	-0.06	8.8	9	-0.01	13.5	15.8	-0.07	13.6	14	-0.01

Psychostimulants, agents used for adhd and nootropics	0.1	0.1	-0.01	0.1	0.2	-0.01	1.1	1.4	-0.03	1.1	1.1	0.00

- Characteristics are based on deep vein thrombosis as the outcome event. Numbers are slightly different for other outcome events, as patients with prior history of that event were excluded from the analysis.

Supplementary Table 4. Baseline characteristics of eligible Ad26.COV2.S, BNT162b2, and mRNA-1273 cohorts of vaccinated people identified US Open Claims.

	**US Open Claims**						**US Open Claims**					
	**Before PS matching**			**After PS matching**			**Before PS matching**			**After PS matching**		
	**Ad26.COV2.S**	**BNT162b2**		**Ad26.COV2.S**	**BNT162b2**		**Ad26.COV2.S**	**mRNA-1273**		**Ad26.COV2.S**	**mRNA-1273**	
**Characteristic**	**%**	**%**	**SMD**	**%**	**%**	**SMD**	**%**	**%**	**SMD**	**%**	**%**	**SMD**
**Cohort Count**	628,004	5,288,749		628,002	2,363,428		628,164	3,923,371		628,164	2,230,157	
**Age group**												
<20	2.2	4.7	-0.14	1.8	1.9	-0.01	2.2	1.6	0.04	1.8	1.9	0.00
20 - 29	13.4	16	-0.07	10.4	10.9	-0.02	13.4	11.5	0.06	10.4	10.9	-0.01
30 - 39	15.2	17	-0.05	12.7	13.1	-0.01	15.2	13.1	0.06	12.7	13	-0.01
40 - 49	17.3	16.9	0.01	16.3	16.5	-0.01	17.3	14.2	0.08	16.3	16.1	0.01
50 - 59	22.1	17.7	0.11	23.5	23.6	0.00	22.1	17.9	0.10	23.5	22.9	0.02
60 - 69	18.9	15.1	0.10	21.8	21.4	0.01	18.9	20.6	-0.04	21.8	21.8	0.00
70 - 79	7.6	8.2	-0.02	9.3	8.6	0.02	7.6	14	-0.21	9.3	9.3	0.00
80 - 89	3.3	4.5	-0.06	4.1	3.9	0.01	3.3	6.9	-0.16	4.1	4.3	-0.01
**Gender: female**	47	54.5	-0.15	52	52.8	-0.02	47	54	-0.14	52	53.4	-0.03
**Index month**												
12		1						0.7				
1		6						8.9				
2		6.9						15.4				
3	23	20	0.07	26.9	29.3	-0.06	23	29.9	-0.16	26.9	30.7	-0.09
4	33.5	21.6	0.27	35.2	34.3	0.02	33.5	23.9	0.21	35.2	35	0.00
5	21.3	11.8	0.26	20	18	0.05	21.3	11.7	0.26	20	18.6	0.04
6	10.5	7.8	0.09	9.2	8.9	0.01	10.5	2.9	0.31	9.2	7.2	0.07
7	5.9	9	-0.12	4.7	5	-0.01	5.9	3.8	0.10	4.7	4.7	0.00
8	5.3	14.1	-0.30	3.7	4.1	-0.02	5.3	2.5	0.15	3.7	3.5	0.01
9	0.5	1.9	-0.13	0.3	0.3	-0.01	0.5	0.2	0.05	0.3	0.3	0.00
**Covid-19 infection prior vaccination**	2.9	3.3	-0.02	3.4	3.6	-0.01	2.9	2.8	0.01	3.4	3.5	0.00
**Charlson index - Romano adaptation**	108.2	113.3	-0.03	137.7	136.2	0.01	108.2	145.6	-0.17	137.7	139.7	-0.01
**CHADS2VASc**	129	138.5	-0.07	155.7	153.2	0.02	129	173.1	-0.29	155.7	158.3	-0.02
**Medical history: General**												
Acute respiratory disease	45.2	45.3	0.00	50.8	52	-0.03	45.2	46.4	-0.02	50.8	51.4	-0.01
Attention deficit hyperactivity disorder	4.4	4.2	0.01	5	5.3	-0.01	4.4	3.5	0.05	5	5	0.00
Chronic obstructive lung disease	5.4	5	0.02	7	6.6	0.02	5.4	7.1	-0.07	7	7.2	-0.01
Crohn's disease	0.5	0.6	0.00	0.7	0.7	0.00	0.5	0.6	-0.01	0.7	0.7	0.00
Dementia	1.2	1.9	-0.06	1.5	1.5	0.00	1.2	1.9	-0.06	1.5	1.5	0.00
Diabetes mellitus	12.9	13	0.00	16.8	16.6	0.01	12.9	17	-0.12	16.8	17.1	-0.01
Gastrointestinal hemorrhage	5.9	5.9	0.00	7.2	7.4	-0.01	5.9	7.2	-0.05	7.2	7.4	-0.01
Human immunodeficiency virus infection	0.4	0.4	-0.01	0.5	0.5	-0.01	0.4	0.4	-0.01	0.5	0.5	0.00
Hyperlipidemia	31.4	29.8	0.03	39.6	39.4	0.01	31.4	38.9	-0.16	39.6	39.7	0.00
Hypertensive disorder	30.7	29.3	0.03	39.4	38.7	0.02	30.7	38.1	-0.16	39.4	39.5	0.00
Lesion of liver	1	1	0.00	1.2	1.3	0.00	1	1.2	-0.02	1.2	1.2	0.00
Obesity	13.7	13.9	-0.01	16.9	17	0.00	13.7	15.4	-0.05	16.9	17	-0.01

Osteoarthritis	25.6	23.4	0.05	32	31.6	0.01	25.6	30.8	-0.12	32	31.9	0.00
Pneumonia	6.9	7.3	-0.02	8.1	8.1	0.00	6.9	8	-0.05	8.1	8.3	0.00
Psoriasis	1.9	1.8	0.00	2.4	2.4	0.00	1.9	2.2	-0.02	2.4	2.4	0.00
Renal impairment	5.4	6	-0.03	6.9	6.8	0.01	5.4	8.1	-0.11	6.9	7.1	-0.01
Rheumatoid arthritis	1.6	1.7	-0.01	2.1	2	0.01	1.6	2.2	-0.04	2.1	2.2	-0.01
Schizophrenia	0.6	0.7	-0.02	0.8	0.8	0.00	0.6	0.7	-0.01	0.8	0.8	0.00
Urinary tract infectious disease	14.3	16.2	-0.05	17.6	18	-0.01	14.3	17.8	-0.10	17.6	18.2	-0.02
Visual system disorder	35.9	36.5	-0.01	41.7	42.1	-0.01	35.9	41.3	-0.11	41.6	42	-0.01
**Medical history: Cardiovascular disease**												
Atrial fibrillation	2.9	3.3	-0.02	3.7	3.5	0.01	2.9	4.7	-0.09	3.7	3.8	0.00
Cerebrovascular disease	4.9	5.2	-0.01	6.4	6.2	0.01	4.9	7.4	-0.10	6.4	6.5	-0.01
Coronary arteriosclerosis	6.1	6.1	0.00	8	7.6	0.02	6.1	9.1	-0.11	8	8.1	0.00
Heart disease	17.9	18.1	0.00	22.7	22.3	0.01	17.9	23.9	-0.15	22.7	22.9	0.00
Heart failure	3.3	3.8	-0.03	4.2	4.1	0.01	3.3	5	-0.09	4.2	4.3	-0.01
Peripheral vascular disease	4	4.4	-0.02	5.1	5	0.01	4	6	-0.09	5.1	5.3	-0.01
Pulmonary embolism	0.8	0.9	-0.01	0.6	0.7	0.00	0.8	1.1	-0.04	0.6	0.7	-0.01
Venous thrombosis	2.1	2.2	-0.01	1.4	1.4	0.00	2.1	2.8	-0.05	1.4	1.4	0.00
**Medical history: Neoplasms**												
Malignant tumor of breast	1.3	1.5	-0.02	1.7	1.7	0.00	1.3	2	-0.06	1.7	1.7	0.00
Hematologic neoplasm	1.1	1.2	-0.01	1.4	1.4	0.00	1.1	1.5	-0.04	1.4	1.4	0.00
Malignant neoplasm of anorectum	0.2	0.2	0.00	0.3	0.3	0.01	0.2	0.3	-0.01	0.3	0.3	0.00
Malignant neoplastic disease	8.5	8.7	-0.01	10.8	10.4	0.01	8.5	12.2	-0.12	10.8	10.7	0.00
Malignant tumor of colon	0.4	0.4	0.00	0.5	0.5	0.00	0.4	0.6	-0.03	0.5	0.5	0.00
Malignant tumor of urinary bladder	0.3	0.3	0.00	0.3	0.3	0.00	0.3	0.4	-0.03	0.3	0.3	0.00
Primary malignant neoplasm of prostate	1	1	-0.01	1.3	1.2	0.01	1	1.6	-0.06	1.3	1.3	0.00
**Medication use**												
Agents acting on the renin-angiotensin system	17.5	15.5	0.05	25.1	24.3	0.02	17.5	21.5	-0.10	25.1	24.7	0.01
Beta blocking agents	10.4	9.8	0.02	14.9	14.3	0.02	10.4	13.8	-0.10	14.8	14.8	0.00
Diuretics	11.4	10.6	0.03	16.2	15.7	0.01	11.4	14.3	-0.09	16.2	16	0.01
Drugs for acid related disorders	10.8	10.5	0.01	15.2	14.8	0.01	10.8	13.4	-0.08	15.2	15.2	0.00
Calcium channel blockers	8.3	7.9	0.02	11.8	11.5	0.01	8.3	10.8	-0.09	11.8	11.8	0.00
Antiinflammatory and antirheumatic products	12.9	12.8	0.00	17.2	17	0.00	12.8	14.2	-0.04	17.2	17.2	0.00
Drugs used in diabetes	8.7	8.2	0.02	12.5	12.2	0.01	8.7	11	-0.08	12.5	12.5	0.00
Antibacterials for systemic use	20.9	21.5	-0.01	26.7	26.9	0.00	20.9	23.1	-0.05	26.7	26.6	0.00
Opioids	7.7	7.1	0.02	10.3	10.1	0.01	7.7	8.3	-0.02	10.3	10.2	0.01
Antineoplastic agents	2.3	2.5	-0.01	3.2	3.2	0.00	2.3	3	-0.04	3.2	3.3	-0.01
Immunosuppressants							1.4	1.9	-0.04	2	2	0.00
Antiepileptics	8.7	7.9	0.03	12.3	12.1	0.01	8.7	9.9	-0.04	12.3	12.1	0.01
Psycholeptics	11.3	10.6	0.02	15.9	15.8	0.00	11.3	12.3	-0.03	15.8	15.5	0.01
Antipsoriatics	0.3	0.3	-0.01	0.4	0.4	0.00	0.3	0.4	-0.01	0.4	0.4	0.00
Antidepressants	17.3	16.2	0.03	24.5	24.7	0.00	17.3	18.7	-0.04	24.5	24.2	0.01
Psychostimulants, agents used for adhd and nootropics	3.4	3.2	0.01	4.9	5.1	-0.01	3.4	2.9	0.03	4.9	4.7	0.01

- Characteristics are based on deep vein thrombosis as the outcome event. Numbers are slightly different for other outcome events, as patients with prior history of that event were excluded from the analysis.

Supplementary Table 5. Baseline characteristics of eligible cohorts of vaccinated people identified in IQVIA LPD France

**Characteristic**	**Before PS matching**			**After PS matching**		
	**ChAdOx1**	**BNT162b2**		**ChAdOx1**	**BNT162b2**	
	**%**	**%**	**SMD**	**%**	**%**	**SMD**

**Cohort count**	25,663	12,244		6,991	15,042	
Age group						
20 - 29	0.3	3.7	-0.24	0.9	0.8	0.01
30 - 39	0.6	4.5	-0.25	1.6	1.3	0.02
40 - 49	1.6	8.7	-0.33	3.9	3.1	0.04
50 - 59	23.2	14.1	0.24	17.9	18.9	-0.02
60 - 69	44.1	18.7	0.57	27.4	28.2	-0.02
70 - 79	24.5	28.4	-0.09	33.0	32.8	0.00
80 - 89	4.5	17.9	-0.43	12.8	12.6	0.01
90 - 99	1.1	3.1	-0.14	2.4	2.2	0.01
Gender: female	47.6	56.4	-0.17	54.5	54.2	0.00
Index month						
2	3.6	9.0	-0.22	6.5	5.9	0.02
3	50.3	20.5	0.66	29.9	31.3	-0.03
4	26.5	22.3	0.10	30.6	31.1	-0.01
5	9.7	17.8	-0.24	18.0	17.2	0.02
6	7.0	10.6	-0.13	10.4	9.9	0.02
7	2.6	6.1	-0.17	4.2	4.2	0.00
8	0.1	3.5	-0.25	0.2	0.2	0.01
Covid-19 history						
Covid-19 infection prior vaccination	4.7	6.1	-0.06	5.0	4.8	0.01
Medical history: General						
Acute respiratory disease	44.2	45.5	-0.02	43.8	43.4	0.01
Chronic liver disease	0.5	0.4	0.02	0.5	0.5	-0.01
Chronic obstructive lung disease	4.9	4.4	0.02	4.5	4.7	-0.01
Crohn's disease	0.2	0.4	-0.03	0.3	0.3	-0.01
Dementia	0.3	0.4	-0.02	0.5	0.4	0.01
Depressive disorder	17.9	19.0	-0.03	18.1	18.7	-0.01
Diabetes mellitus	19.9	15.3	0.12	16.7	17.3	-0.01
Gastroesophageal reflux disease	16.3	18.2	-0.05	18.1	18.4	-0.01
Gastrointestinal hemorrhage	3.3	3.8	-0.03	3.5	3.6	0.00
Human immunodeficiency virus infection	0.6	0.4	0.04	0.4	0.4	0.00
Hyperlipidemia	20.8	22.1	-0.03	23.0	23.2	-0.01
Hypertensive disorder	44.1	44.1	0.00	46.9	47.3	-0.01
Lesion of liver	0.6	0.5	0.01	0.6	0.5	0.01
Obesity	1.3	0.9	0.04	1.0	0.9	0.01
Osteoarthritis	22.3	22.7	-0.01	23.5	23.8	-0.01
Pneumonia	4.5	4.8	-0.01	4.6	4.9	-0.01
Psoriasis	4.4	4.2	0.01	4.4	4.5	0.00
Renal impairment	1.4	2.5	-0.08	2.1	2.2	0.00
Rheumatoid arthritis	0.7	1.0	-0.03	0.9	0.9	0.00
Schizophrenia	0.1	0.1	0.01	0.1	<0.1	0.00
Ulcerative colitis	0.3	0.3	-0.01	0.2	0.3	-0.02

Urinary tract infectious disease	7.1	8.9	-0.06	7.9	8.2	-0.01
Viral hepatitis C	0.3	0.2	0.02	0.2	0.3	-0.01
Visual system disorder	21.0	23.7	-0.06	22.6	22.1	0.01
Medical history: Cardiovascular disease						
Atrial fibrillation	0.8	1.5	-0.07	1.1	1.2	0.00
Cerebrovascular disease	4.0	5.2	-0.06	3.0	3.2	-0.01
Coronary arteriosclerosis	2.2	2.3	-0.01	2.5	2.3	0.01
Heart disease	14.7	18.1	-0.09	17.3	16.9	0.01
Heart failure	1.2	2.1	-0.07	1.9	1.7	0.02
Ischemic heart disease	4.9	4.8	0.00	4.8	5.0	-0.01
Peripheral vascular disease	1.6	1.3	0.02	1.5	1.6	0.00
Pulmonary embolism	0.7	1.1	-0.04	0.9	0.9	-0.01
Venous thrombosis	2.0	2.8	-0.05	2.5	2.6	-0.01
Medical history: Neoplasms						
Hematologic neoplasm	0.3	0.5	-0.03	0.5	0.5	0.00
Malignant lymphoma	0.3	0.6	-0.05	0.5	0.6	-0.01
Malignant neoplasm of anorectum	1.5	0.9	0.06	0.9	1.0	-0.01
Malignant neoplastic disease	7.8	9.2	-0.05	8.6	9.0	-0.01
Malignant tumor of breast	1.8	2.2	-0.03	2.2	2.2	0.00
Malignant tumor of colon	1.5	0.9	0.06	1.0	1.1	0.00
Malignant tumor of urinary bladder	0.2	0.4	-0.03	0.3	0.3	0.00
Primary malignant neoplasm of prostate	1.7	1.7	0.00	1.7	1.7	0.00
Medication use						
Agents acting on the renin-angiotensin system	35.6	33.5	0.04	36.8	37.2	-0.01
Antidepressants	10.3	11.4	-0.03	10.9	11.1	0.00
Antiepileptics	4.0	4.1	-0.01	4.3	4.4	0.00
Antiinflammatory and antirheumatic products	34.5	34.7	0.00	34.7	34.8	0.00
Antineoplastic agents	0.9	0.9	0.00	0.9	1.0	0.00
Antipsoriatics	0.5	0.6	-0.01	0.6	0.6	0.00
Antithrombotic agents	22.8	25.9	-0.07	24.6	24.3	0.01
Beta blocking agents	19.7	21.6	-0.04	22.3	22.4	0.00
Diuretics	19.5	20.4	-0.02	21.7	21.8	0.00
Drugs for acid related disorders	28.0	30.1	-0.04	30.1	30.4	0.00
Drugs for obstructive airway diseases	17.7	17.9	-0.01	18.1	18.2	0.00
Drugs used in diabetes	17.2	12.9	0.12	14.4	14.8	-0.01
Immunosuppressants	0.4	0.7	-0.04	0.6	0.6	0.00
Lipid modifying agents	31.5	29.1	0.05	31.4	31.7	-0.01
Opioids	13.5	12.9	0.02	13.3	13.0	0.01
Psycholeptics	17.3	18.4	-0.03	18.8	18.7	0.00
Psychostimulants, agents used for adhd and nootropics	3.0	3.3	-0.02	3.3	3.3	0.00

- Characteristics are based on deep vein thrombosis as the outcome event. Numbers are slightly different for other outcome events, as patients with prior history of that event were excluded from the analysis.

Supplementary Table 6. Baseline characteristics of eligible cohorts of vaccinated people identified in IPCI

Characteristic	Before PS matching			After PS matching		
	ChAdOx1	BNT162b2		ChAdOx1	BNT162b2	
	%	%	SMD	%	%	SMD
**Cohort count**	70,427	208,713		22,039	49,198	
Age group						
10 - 19	0.2	0.8	-0.08	0.5	0.4	0.01
20 - 29	1.7	5.2	-0.19	4.1	3.6	0.03
30 - 39	1.8	11.3	-0.39	4.6	3.7	0.04
40 - 49	3.0	9.1	-0.26	6.6	5.7	0.04
50 - 59	5.7	21.3	-0.47	13.0	10.7	0.07
60 - 69	83.5	15.0	1.89	59.6	61.5	-0.04
70 - 79	1.7	26.5	-0.76	5.2	7.5	-0.09
80 - 89	1.4	9.9	-0.38	3.9	4.1	-0.01
90 - 99	0.9	1.0	-0.02	2.4	2.7	-0.02
Gender: female	50.3	51.2	-0.02	54.6	55.2	-0.01
Index month						
2	3.0	0.2	0.23	1.1	1.1	0.00
3	18.5	7.6	0.33	7.4	7.5	0.00
4	42.4	24.0	0.40	22.5	24.5	-0.05
5	29.4	30.7	-0.03	52.3	52.3	0.00
6	6.8	37.5	-0.80	16.6	14.5	0.06
Covid-19 history						
Covid-19 infection prior vaccination	6.9	5.4	0.065	6.4	6.3	0.003
Medical history: General						
Attention deficit hyperactivity disorder	1.0	1.5	-0.05	1.4	1.3	0.01
Chronic liver disease	0.2	0.2	0.01	0.2	0.2	0.00
Chronic obstructive lung disease	6.0	5.1	0.04	5.6	6.1	-0.02
Crohn's disease	0.4	0.5	-0.01	0.4	0.4	0.00
Dementia	0.5	1.0	-0.06	1.0	1.2	-0.02
Depressive disorder	11.4	9.3	0.07	11.1	11.2	0.00
Diabetes mellitus	12.5	12.6	0.00	12.4	13.0	-0.02
Gastroesophageal reflux disease	3.4	3.5	-0.01	3.3	3.5	-0.01
Gastrointestinal hemorrhage	6.1	5.8	0.01	5.9	6.1	-0.01
Human immunodeficiency virus infection	0.2	0.1	0.01	0.2	0.2	0.00
Hyperlipidemia	14.8	13.2	0.05	13.1	13.9	-0.02
Hypertensive disorder	32.7	31.3	0.03	30.6	32.6	-0.04
Obesity	8.0	4.9	0.13	7.7	7.7	0.00
Osteoarthritis	18.4	19.7	-0.03	18.3	19.8	-0.04
Pneumonia	11.1	11.4	-0.01	11.3	11.8	-0.02
Psoriasis	4.7	4.3	0.02	4.4	4.6	-0.01
Renal impairment	3.7	6.8	-0.14	5.0	5.6	-0.03
Rheumatoid arthritis	2.7	2.9	-0.01	2.8	3.0	-0.01
Schizophrenia	0.3	0.2	0.02	0.3	0.4	0.00
Urinary tract infectious disease	24.1	26.2	-0.05	26.6	27.2	-0.01
Viral hepatitis C	0.1	0.1	0.02	0.1	0.1	0.01
Visual system disorder	48.5	51.9	-0.07	49.1	50.3	-0.02
Medical history: Cardiovascular disease						
Cerebrovascular disease	4.7	5.7	-0.05	3.3	3.7	-0.03
Coronary arteriosclerosis	0.8	0.8	0.00	0.8	0.8	0.00
Heart disease	17.1	19.6	-0.06	17.5	18.9	-0.04

Heart failure	1.8	2.4	-0.04	2.5	2.8	-0.02
Ischemic heart disease	8.1	9.0	-0.04	8.2	8.8	-0.02
Peripheral vascular disease	1.3	1.4	-0.01	1.4	1.6	-0.01
Venous thrombosis	3.6	3.8	-0.01	3.9	4.3	-0.02
Medical history: Neoplasms						
Hematologic neoplasm	0.2	0.3	-0.01	0.2	0.2	0.00
Malignant lymphoma	0.2	0.3	-0.01	0.2	0.2	-0.01
Malignant neoplastic disease	14.8	17.3	-0.07	15.3	16.1	-0.02
Malignant tumor of breast	2.9	2.8	0.01	2.8	2.9	-0.01
Malignant tumor of urinary bladder	0.5	0.7	-0.03	0.5	0.5	0.00
Medication use						
Agents acting on the renin-angiotensin system	20.9	21.5	-0.01	19.9	21.3	-0.03
Antibacterials for systemic use	12.9	14.5	-0.05	14.6	15.3	-0.02
Antidepressants	9.1	7.9	0.04	8.9	9.3	-0.02
Antiepileptics	2.9	2.6	0.02	3.0	3.1	-0.01
Antineoplastic agents	1.7	2.4	-0.04	2.0	2.1	-0.01
Antipsoriatics	0.5	0.5	0.00	0.5	0.6	-0.01
Antithrombotic agents	13.7	17.5	-0.10	13.7	15.0	-0.04
Beta blocking agents	14.3	17.2	-0.08	14.9	16.1	-0.04
Calcium channel blockers	11.0	11.4	-0.01	10.2	11.1	-0.03
Diuretics	13.3	13.7	-0.01	13.0	14.3	-0.04
Drugs for acid related disorders	25.0	27.3	-0.05	25.6	27.2	-0.04
Drugs for obstructive airway diseases	20.6	20.7	0.00	21.3	21.8	-0.01
Drugs used in diabetes	8.6	8.5	0.00	8.6	9.0	-0.01
Immunosuppressants	1.4	1.9	-0.04	1.7	1.8	-0.01
Lipid modifying agents	22.7	23.6	-0.02	20.8	22.3	-0.04
Opioids	7.0	6.4	0.03	7.3	7.7	-0.01
Psycholeptics	9.9	9.8	0.00	10.6	11.0	-0.01
Psychostimulants, agents used for adhd and nootropics	0.5	0.7	-0.03	0.7	0.7	0.00

- Characteristics are based on deep vein thrombosis as the outcome event. Numbers are slightly different for other outcome events, as patients with prior history of that event were excluded from the analysis.

Supplementary Table 7. Baseline characteristics of eligible cohorts of vaccinated people identified in IQVIA US Hospital CDM

**Characteristic**	**Before PS matching**			**After PS matching**		
	**Ad26.COV2.S**	**BNT162b2**		**Ad26.COV2.S**	**BNT162b2**	
	**%**	**%**	**SMD**	**%**	**%**	**SMD**
Coort Count	1,680	243,796		1,658	6,317	
Age group						
10 - 19	1.1	1.6	-0.04	0.7	0.5	0.03
20 - 29	8.1	6.5	0.06	4.6	4.0	0.03
30 - 39	13.0	9.2	0.12	9.4	9.2	0.01
40 - 49	14.7	11.5	0.10	11.8	11.0	0.03
50 - 59	25.4	15.9	0.24	26.4	27.9	-0.03
70 - 79	7.9	23.2	-0.43	9.5	10.1	-0.02
80 - 89	2.2	9.0	-0.30	2.4	2.3	0.01
Gender: female	54.3	59.1	-0.10	58.8	59.9	-0.02
Index month						
3	31.1	33.7	-0.06	49.5	50.2	-0.01
4	62.6	20.6	0.94	44.1	43.6	0.01
5	3.1	3.3	-0.02	3.3	3.4	-0.01

6	2.1	1.2	0.07	2.5	2.4	0.01
7	1.1	0.5	0.07	0.7	0.4	0.05
Covid-19 history						
Covid-19 infection prior vaccination	1.9	1.8	0.01	3.4	3.8	-0.02
Medical history: General						
Attention deficit hyperactivity disorder	1.1	1.0	0.01	1.0	1.1	-0.02
Chronic liver disease	2.9	2.3	0.04	5.6	5.2	0.02
Chronic obstructive lung disease	5.0	5.4	-0.02	8.8	8.5	0.01
Crohn's disease	0.5	0.6	-0.01	0.8	0.7	0.01
Depressive disorder	10.9	11.1	-0.01	16.7	16.9	-0.01
Diabetes mellitus	13.9	16.9	-0.08	23.0	23.4	-0.01
Gastroesophageal reflux disease	14.9	18.3	-0.09	24.8	25.6	-0.02
Gastrointestinal hemorrhage	3.6	3.5	0.01	6.0	5.9	0.00
Human immunodeficiency virus infection	1.0	0.8	0.02	1.9	1.6	0.02
Hyperlipidemia	24.8	36.0	-0.25	37.5	39.5	-0.04
Hypertensive disorder	34.0	40.8	-0.14	52.1	54.5	-0.05
Lesion of liver	1.6	1.5	0.01	3.1	2.7	0.02
Obesity	13.2	15.1	-0.06	21.3	22.1	-0.02
Osteoarthritis	15.2	21.3	-0.16	25.7	26.6	-0.02
Pneumonia	3.7	4.0	-0.02	6.1	5.8	0.01
Psoriasis	0.6	1.0	-0.04	1.0	1.1	-0.01
Renal impairment	6.5	8.7	-0.08	12.7	12.6	0.00
Rheumatoid arthritis	1.1	1.7	-0.06	2.2	2.1	0.01
Schizophrenia	0.8	0.3	0.07	1.6	1.4	0.01
Ulcerative colitis	0.3	0.5	-0.03	0.5	0.5	0.01
Urinary tract infectious disease	8.4	9.2	-0.03	12.6	12.0	0.02
Viral hepatitis C	2.3	1.0	0.10	5.0	4.2	0.04
Visual system disorder	9.9	15.0	-0.16	16.6	17.9	-0.03
Medical history: Cardiovascular disease						
Atrial fibrillation	2.6	5.6	-0.15	4.3	4.8	-0.02
Cerebrovascular disease	2.6	4.2	-0.09	3.1	3.4	-0.02
Coronary arteriosclerosis	6.0	10.1	-0.15	10.2	9.8	0.01
Heart disease	14.2	22.5	-0.21	22.0	21.8	0.00
Heart failure	3.5	4.6	-0.06	6.9	6.3	0.03
Ischemic heart disease	4.0	6.0	-0.09	7.3	7.0	0.01
Peripheral vascular disease	2.9	3.6	-0.04	6.4	5.8	0.03
Pulmonary embolism	1.0	1.0	0.00	1.6	1.7	-0.01
Venous thrombosis	1.5	2.0	-0.04	2.6	2.4	0.01
Medical history: Neoplasms						
Hematologic neoplasm	0.9	2.0	-0.09	2.0	2.0	0.00
Malignant lymphoma	0.4	0.9	-0.07	0.5	0.7	-0.03
Malignant neoplasm of anorectum	0.2	0.3	-0.02	0.4	0.3	0.00
Malignant neoplastic disease	6.5	11.6	-0.18	11.4	11.6	-0.01
Malignant tumor of breast	1.6	2.9	-0.09	2.7	2.7	0.00
Malignant tumor of colon	0.3	0.6	-0.04	0.7	0.7	0.00
Malignant tumor of urinary bladder	0.2	0.4	-0.05	0.4	0.3	0.01
Primary malignant neoplasm of prostate	1.1	2.0	-0.07	2.1	2.3	-0.01
Medication use						
Agents acting on the renin-angiotensin system	1.3	1.1	0.02	3.0	2.8	0.01
Antibacterials for systemic use	5.3	4.2	0.05	11.0	10.7	0.01
Antidepressants	1.5	1.0	0.04	3.2	3.2	0.00
Antiepileptics	1.7	1.2	0.05	4.0	3.6	0.03

Antiinflammatory and antirheumatic products	5.1	3.4	0.08	10.6	10.1	0.02
Antineoplastic agents	0.6	1.0	-0.04	1.9	1.6	0.02
Antithrombotic agents	3.9	3.6	0.01	8.0	7.9	0.01
Beta blocking agents	2.0	1.6	0.03	4.6	3.8	0.04
Diuretics	1.4	1.1	0.03	3.1	3.5	-0.02
Drugs for acid related disorders	3.0	2.5	0.03	6.2	5.6	0.02
Drugs for obstructive airway diseases	4.1	3.7	0.02	8.9	8.3	0.02
Drugs used in diabetes	0.2	0.3	-0.01	0.5	0.7	-0.02
Immunosuppressants	0.2	0.3	-0.03	0.5	0.6	0.00
Lipid modifying agents	1.8	1.6	0.02	3.5	3.7	-0.01
Opioids	5.5	5.0	0.02	12.1	11.4	0.02
Psycholeptics	5.7	4.7	0.05	11.8	11.7	0.00
Psychostimulants, agents used for adhd and nootropics	0.1	0.1	0.00	<0.3	0.2	-0.01

- Characteristics are based on deep vein thrombosis as the outcome event. Numbers are slightly different for other outcome events, as patients with prior history of that event were excluded from the analysis.

Supplementary Table 8. Summary of diagnostics at the database-target-comparator level.

DATABASE	DIAGNOSTIC		***1st dose ChAdOx1 vs BNT162b2***	***1st dose ChAdOx1 vs mRNA-1273***	***2nd dose ChAdOx1 vs BNT162b2***	***2nd dose ChAdOx1 vs mRNA-1273***	***Ad26.COV2.S vs BNT162b2***	***Ad26.COV2.S vs mRNA-1273***
**UK CPRD**								
	**Covariate balance**	*SMD<0.1 for all*	✓	n/a	✓	n/a	n/a	n/a
	**Power**	*MDRR<5 for 1+ outcomes*	10	n/a	9	n/a	n/a	n/a
	**Systematic error**	*<20% associated w exposure*	98.0%	n/a	94.4%	n/a	n/a	n/a
**Spain SIDIAP**								
	**Covariate balance**	*SMD<0.1 for all*	x	x	x	x	✓	n/a
	**Power**	*4<5 for 1+ outcomes*					8	n/a
	**Systematic error**	*<20% associated w exposure*					96.4%	n/a
**GERMANY DA**								
	**Covariate balance**	*SMD<0.1 for all*	✓	x	✓	x	✓	n/a
	**Power**	*MDRR<5 for 1+ outcomes*	7	0	0	0	1	n/a
	**Systematic error**	*<20% associated w exposure*	84.2%		$		70.0%	n/a
**FRANCE LPD**								
	**Covariate balance**	*SMD<0.1 for all*	✓	✓	x	x	n/a	n/a
	**Power**	*MDRR<5 for 1+ outcomes*	0	0	0	0	n/a	n/a
	**Systematic error**	*<20% associated w exposure*	83.3%	$			n/a	n/a
**Netherlands IPCI**								

	**Covariate balance**	*SMD<0.1 for all*	✓	n/a	✓	n/a	✓	✓
	**Power**	*MDRR<5 for 1+ outcomes*	3	n/a	0	n/a	0	0
	**Systematic error**	*<20% associated w exposure*	$	n/a	$	n/a	$	$
**IQVIA US OpenClaims**								
	**Covariate balance**	*SMD<0.1 for all*	n/a	n/a	n/a	n/a	✓	✓
	**Power**	*MDRR<5 for 1+ outcomes*	n/a	n/a	n/a	n/a	12	13
	**Systematic error**	*<20% associated w exposure*	n/a	n/a	n/a	n/a	72.2%	67.9%
**IQVIA US HOSPITAL CDM**								
	**Covariate balance**	*SMD<0.1 for all*	n/a	n/a	n/a	n/a	x	x
	**Power**	*MDRR<5 for 1+ outcomes*	n/a	n/a	n/a	n/a	0	0
	**Systematic error**	*<20% associated w exposure*	n/a	n/a	n/a	n/a		

Grey = Excluded from further analyses; Orange = Included for meta-analysis, underpowered for database-specific effect estimation; Green = Systematic error detected, only calibrated IRRs should be interpreted. $: empirical calibration was not conducted due to insufficient NCOs estimated.

Supplementary Table 9. Crude incidence rate and corresponding 95% confidence intercals, European data

	UK CPRD				Germany DA				SIDIAP			
Vaccine	N	Person-year	Events	IR per 1,000 person-years (95% CI)	N	Person-year	Events	IR per 1,000 person-years (95% CI)	N	Person-year	Events	IR per 1,000 person-years (95% CI)
**Thrombocytopenia**												
ChAdOx1 1st dose	3,621,898	263,673	1,261	4.78 (4.52 to 5.05)	85,500	6,480	37	5.71 (4.02 to 7.87)				
ChAdOx1 2nd dose	1,115,370	54,189	347	6.4 (5.75 to 7.11)	17,426	1,219	<5					
BNT162b2 1st dose	1,724,118	130,845	641	4.9 (4.53 to 5.29)	334,413	25,186	116	4.61 (3.81 to 5.52)	1,754,222	109,785	1,516	13.81 (13.12 to 14.52)
BNT162b2 2nd dose	1,276,204	80,349	458	5.7 (5.19 to 6.25)	170,133	12,124	59	4.87 (3.7 to 6.28)	1,131,167		1,242	16.45 (15.54 to 17.39)
mRNA -1273 1st dose					614	46	<5		215,405	15,527	416	26.79 (24.28 to 29.49)
mRNA -1273 2nd dose					72	5	0	0 (0 to 694.52)	158,659	8,191	310	37.85 (33.75 to 42.3)
Ad26.COV2.S					17,988	1,217	13	10.68 (5.69 to 18.27)	115,521	5,434	57	10.49 (7.94 to 13.59)
**Any thrombosis (venous thrombo-embolism or arterial thromboembolism) with thrombocytopenia syndrome (Any-TTS)**												
ChAdOx1 1st dose	3,772,477	274,860	197	0.72 (0.62 to 0.82)								
ChAdOx1 2nd dose	1,186,267	57,864	51	0.88 (0.66 to 1.16)								
BNT162b2 1st dose	1,827,767	138,755	98	0.71 (0.57 to 0.86)					1,990,976	126,431	0	0 (0 to 0.03)
BNT162b2 2nd dose	1,358,901	85,737	68	0.79 (0.62 to 1.01)					1,328,942	89,897	0	0 (0 to 0.04)
mRNA -1273 1st dose									246,113	17,799	0	0 (0 to 0.21)
mRNA -1273 2nd dose									184,804	9,891	0	0 (0 to 0.37)
Ad26.COV2.S									126,950	6,054	0	0 (0 to 0.61)
**TTS_ATE**												
ChAdOx1 1st dose	3,788,372	276,051	7	0.03 (0.01 to 0.05)	89,691	6,799	0	0 (0 to 0.54)				
ChAdOx1 2nd dose	1,194,498	58,293	<5		18,765	1,316	0	0 (0 to 2.8)				
BNT162b2 1st dose	1,839,463	139,647	6	0.04 (0.02 to 0.09)	350,881	26,434	<5		1,987,022	126,133	80	0.63 (0.5 to 0.79)
BNT162b2 2nd dose	1,368,269	86,343	<5		181,708	12,971	0	0 (0 to 0.28)	1,325,147	89,614	74	0.83 (0.65 to 1.04)
mRNA -1273 1st dose					653	49	0	0 (0 to 74.7)	245,696	17,768	10	0.56 (0.27 to 1.03)
mRNA -1273 2nd dose					72	5	0	0 (0 to 694.52)	184,428	9,864	5	0.51 (0.16 to 1.18)

Ad26.COV2.S					18,757	1,271	0	0 (0 to 2.9)	126,844	6,048	<5	
**TTS_CVST**												
ChAdOx1 1st dose	3,788,833	276,086	<5		89,718	6,802	0	0 (0 to 0.54)				
ChAdOx1 2nd dose	1,194,815	58,310	0	0 (0 to 0.06)	18,774	1,316	0	0 (0 to 2.8)				
BNT162b2 1st dose	1,839,964	139,685	0	0 (0 to 0.03)	351,008	26,444	<5		1,990,968	126,430	0	0 (0 to 0.03)
BNT162b2 2nd dose	1,368,704	86,372	0	0 (0 to 0.04)	181,818	12,979	0	0 (0 to 0.28)	1,328,935	89,897	0	0 (0 to 0.04)
mR -1273 1st dose					653	49	0	0 (0 to 74.7)	246,109	17,799	0	0 (0 to 0.21)
mR -1273 2nd dose					72	5	0	0 (0 to 694.52)	184,800	9,890	0	0 (0 to 0.37)
Ad26.COV2.S					18,763	1,272	0	0 (0 to 2.9)	126,949	6,054	0	0 (0 to 0.61)
**Deep vein thrombosis with thrombocytopenia syndrome (TTS-DVT)**												
ChAdOx1 1st dose	3,788,419	276,054	10	0.04 (0.02 to 0.07)	89,706	6,801	0	0 (0 to 0.54)				
ChAdOx1 2nd dose	1,194,560	58,298	<5		18,770	1,316	0	0 (0 to 2.8)				
BNT162b2 1st dose	1,839,609	139,658	<5		350,943	26,439	<5		1,990,183	126,371	18	0.14 (0.08 to 0.23)
BNT162b2 2nd dose	1,368,413	86,353	7	0.08 (0.03 to 0.17)	181,768	12,976	<5		1,328,197	89,842	11	0.12 (0.06 to 0.22)
mRNA -1273 1st dose					652	49	0	0 (0 to 74.82)	245,907	17,784	5	0.28 (0.09 to 0.66)
mRNA -1273 2nd dose					72	5	0	0 (0 to 694.52)	184,624	9,878	6	0.61 (0.22 to 1.32)
Ad26.COV2.S					18,758	1,271	0	0 (0 to 2.9)	126,923	6,053	<5	
**TTS_ATE**												
ChAdOx1 1st dose	3,788,766	276,081	<5		89,704	6,800	0	0 (0 to 0.54)				
ChAdOx1 2nd dose	1,194,766	58,307	<5		18,771	1,316	0	0 (0 to 2.8)				
BNT162b2 1st dose	1,839,898	139,680	<5		350,954	26,440	<5		1,988,798	126,266	41	0.32 (0.23 to 0.44)
BNT162b2 2nd dose	1,368,650	86,368	<5		181,769	12,976	0	0 (0 to 0.28)	1,326,837	89,739	39	0.43 (0.31 to 0.59)
mRNA -1273 1st dose					653	49	0	0 (0 to 74.7)	245,927	17,786	5	0.28 (0.09 to 0.66)
mRNA -1273 2nd dose					72	5	0	0 (0 to 694.52)	184,639	9,879	<5	
Ad26.COV2.S					18,762	1,272	0	0 (0 to 2.9)	126,893	6,051	0	0 (0 to 0.61)
**TTS_MI**												
ChAdOx1 1st dose	3,788,454	276,057	<5		89,705	6,801	0	0 (0 to 0.54)				
ChAdOx1 2nd dose	1,194,555	58,296	<5		18,768	1,316	0	0 (0 to 2.8)				
BNT162b2 1st dose	1,839,542	139,653	<5		350,932	26,438	0	0 (0 to 0.14)	1,989,318	126,306	36	0.29 (0.2 to 0.39)
BNT162b2 2nd dose	1,368,336	86,347	<5		181,754	12,974	0	0 (0 to 0.28)	1,327,361	89,781	34	0.38 (0.26 to 0.53)
mRNA -1273 1st dose					653	49	0	0 (0 to 74.7)	245,917	17,785	<5	
mRNA -1273 2nd dose					72	5	0	0 (0 to 694.52)	184,621	9,878	<5	
Ad26.COV2.S					18,758	1,271	0	0 (0 to 2.9)	126,902	6,051	<5	
**TTS_PE**												
ChAdOx1 1st dose	3,788,598	276,068	7	0.03 (0.01 to 0.05)	89,698	6,800	<5					
ChAdOx1 2nd dose	1,194,665	58,302	0	0 (0 to 0.06)	18,770	1,316	0	0 (0 to 2.8)				
BNT162b2 1st dose	1,839,770	139,670	<5		350,891	26,435	<5		1,990,144	126,368	28	0.22 (0.15 to 0.32)
BNT162b2 2nd dose	1,368,545	86,362	<5		181,732	12,973	<5		1,328,162	89,839	17	0.19 (0.11 to 0.3)
mRNA -1273 1st dose					651	49	0	0 (0 to 74.94)	245,920	17,785	9	0.51 (0.23 to 0.96)
mRNA -1273 2nd dose					72	5	0	0 (0 to 694.52)	184,642	9,879	<5	
Ad26.COV2.S					18,759	1,272	<5		126,917	6,052	0	0 (0 to 0.61)
**TTS_SVT**												
ChAdOx1 1st dose	3,788,796	276,083	<5		89,714	6,801	0	0 (0 to 0.54)				
ChAdOx1 2nd dose	1,194,797	58,309	0	0 (0 to 0.06)	18,772	1,316	0	0 (0 to 2.8)				
BNT162b2 1st dose	1,839,935	139,683	0	0 (0 to 0.03)	350,997	26,443	0	0 (0 to 0.14)	1,990,776	126,416	<5	
BNT162b2 2nd dose	1,368,682	86,371	0	0 (0 to 0.04)	181,809	12,979	0	0 (0 to 0.28)	1,328,759	89,884	5	0.06 (0.02 to 0.13)
mRNA -1273 1st dose					653	49	0	0 (0 to 74.7)	245,964	17,788	<5	
mRNA -1273 2nd dose					72	5	0	0 (0 to 694.52)	184,680	9,882	0	0 (0 to 0.37)
Ad26.COV2.S					18,763	1,272	0	0 (0 to 2.9)	126,942	6,054	0	0 (0 to 0.61)
**Venous thrombo-embolism with thrombocytopenia syndrome (TTS-VTE)**												
ChAdOx1 1st dose	3,788,220	276,039	14	0.05 (0.03 to 0.09)	89,697	6,800	0	0 (0 to 0.54)				
ChAdOx1 2nd dose	1,194,431	58,291	<5		18,767	1,316	0	0 (0 to 2.8)				

BNT162b2 1st dose	1,839,448	139,646	5	0.04 (0.01 to 0.08)	350,877	26,434	<5		1,989,653	126,332	39	0.31 (0.22 to 0.42)
BNT162b2 2nd dose	1,368,277	86,344	9	0.1 (0.05 to 0.2)	181,720	12,972	<5		1,327,705	89,805	25	0.28 (0.18 to 0.41)
mRNA -1273 1st dose					651	49	0	0 (0 to 74.94)	245,790	17,775	15	0.84 (0.47 to 1.39)
mRNA -1273 2nd dose					72	5	0	0 (0 to 694.52)	184,523	9,870	9	0.91 (0.42 to 1.73)
Ad26.COV2.S					18,755	1,271	<5		126,901	6,051	<5	
**Cerebral venous sinus thrombosis**												
ChAdOx1 1st dose	3,788,249	276,041	13	0.05 (0.03 to 0.08)	89,711	6,801	<5					
ChAdOx1 2nd dose	1,194,604	58,300	<5		18,770	1,316	0	0 (0 to 2.8)				
BNT162b2 1st dose	1,839,631	139,660	0	0 (0 to 0.03)	350,965	26,441	<5		1,990,871	126,424	<5	
BNT162b2 2nd dose	1,368,495	86,361	0	0 (0 to 0.04)	181,790	12,977	0	0 (0 to 0.28)	1,328,866	89,892	0	0 (0 to 0.04)
mRNA -1273 1st dose					653	49	0	0 (0 to 74.7)	246,081	17,797	0	0 (0 to 0.21)
mRNA -1273 2nd dose					72	5	0	0 (0 to 694.52)	184,775	9,889	<5	
Ad26.COV2.S					18,762	1,272	<5		126,938	6,053	0	0 (0 to 0.61)
**Splanchnic and Visceral Thrombosis**												
ChAdOx1 1st dose	3,787,759	276,004	17	0.06 (0.04 to 0.1)	89,698	6,800	0	0 (0 to 0.54)				
ChAdOx1 2nd dose	1,194,267	58,283	5	0.09 (0.03 to 0.2)	18,767	1,316	0	0 (0 to 2.8)				
BNT162b2 1st dose	1,839,281	139,633	5	0.04 (0.01 to 0.08)	350,832	26,431	<5		1,989,938	126,356	15	0.12 (0.07 to 0.2)
BNT162b2 2nd dose	1,368,176	86,340	<5		181,688	12,970	<5		1,328,039	89,832	14	0.16 (0.09 to 0.26)
mRNA -1273 1st dose					653	49	0	0 (0 to 74.7)	245,579	17,760	7	0.39 (0.16 to 0.81)
mRNA -1273 2nd dose					72	5	0	0 (0 to 694.52)	184,343	9,859	6	0.61 (0.22 to 1.32)
Ad26.COV2.S					18,759	1,271	0	0 (0 to 2.9)	126,887	6,051	<5	
**Deep vein thrombosis (DVT)**												
ChAdOx1 1st dose	3,741,359	272,472	335	1.23 (1.1 to 1.37)	88,463	6,706	21	3.13 (1.94 to 4.79)				
ChAdOx1 2nd dose	1,169,773	57,025	86	1.51 (1.21 to 1.86)	18,390	1,288	0	0 (0 to 2.86)				
BNT162b2 1st dose	1,804,763	136,994	236	1.72 (1.51 to 1.96)	344,916	25,982	33	1.27 (0.87 to 1.78)	1,973,028	125,127	219	1.75 (1.53 to 2)
BNT162b2 2nd dose	1,341,717	84,648	143	1.69 (1.42 to 1.99)	177,348	12,651	27	2.13 (1.41 to 3.11)	1,312,985	88,727	170	1.92 (1.64 to 2.23)
mRNA -1273 1st dose					634	47	<5		243,786	17,626	36	2.04 (1.43 to 2.83)
mRNA -1273 2nd dose					71	5	0	0 (0 to 704.69)	182,802	9,759	26	2.66 (1.74 to 3.9)
Ad26.COV2.S					18,485	1,252	<5		126,189	6,012	10	1.66 (0.8 to 3.06)
Pulmonary embolism												
ChAdOx1 1st dose	3,757,176	273,676	387	1.41 (1.28 to 1.56)	88,838	6,734	6	0.89 (0.33 to 1.94)				
ChAdOx1 2nd dose	1,176,965	57,391	70	1.22 (0.95 to 1.54)	18,500	1,296	<5					
BNT162b2 1st dose	1,815,404	137,808	275	2 (1.77 to 2.25)	345,384	26,017	36	1.38 (0.97 to 1.92)	1,978,276	125,496	177	1.41 (1.21 to 1.63)
BNT162b2 2nd dose	1,349,726	85,156	122	1.43 (1.19 to 1.71)	177,597	12,667	20	1.58 (0.96 to 2.44)	1,317,392	89,043	131	1.47 (1.23 to 1.75)
mRNA -1273 1st dose					643	48	0	0 (0 to 75.88)	244,418	17,674	48	2.72 (2 to 3.6)
mRNA -1273 2nd dose					71	5	0	0 (0 to 704.69)	183,339	9,793	23	2.35 (1.49 to 3.52)
Ad26.COV2.S					18,581	1,259	<5		126,442	6,024	5	0.83 (0.27 to 1.94)
**Venous thrombo-embolism (VTE)**												
ChAdOx1 1st dose	3,715,771	270,525	656	2.42 (2.24 to 2.62)	87,718	6,649	25	3.76 (2.43 to 5.55)				
ChAdOx1 2nd dose	1,155,369	56,282	143	2.54 (2.14 to 2.99)	18,163	1,272	<5					
BNT162b2 1st dose	1,784,864	135,473	462	3.41 (3.11 to 3.74)	340,391	25,639	59	2.3 (1.75 to 2.97)	1,964,216	124,479	358	2.88 (2.59 to 3.19)
BNT162b2 2nd dose	1,326,295	83,658	248	2.96 (2.61 to 3.36)	173,977	12,401	43	3.47 (2.51 to 4.67)	1,304,959	88,132	273	3.1 (2.74 to 3.49)
mRNA -1273 1st dose					629	47	<5		242,639	17,541	79	4.5 (3.57 to 5.61)
mRNA -1273 2nd dose					70	5	0	0 (0 to 715.16)	181,795	9,691	42	4.33 (3.12 to 5.86)
Ad26.COV2.S					18,332	1,241	8	6.45 (2.78 to 12.7)	125,850	5,992	14	2.34 (1.28 to 3.92)
**Ischaemic stroke**												
ChAdOx1 1st dose	3,776,958	275,183	95	0.35 (0.28 to 0.42)	88,152	6,682	13	1.95 (1.04 to 3.33)				
ChAdOx1 2nd dose	1,187,113	57,901	33	0.57 (0.39 to 0.8)	18,246	1,278	0	0 (0 to 2.89)				
BNT162b2 1st dose	1,829,692	138,900	118	0.85 (0.7 to 1.02)	344,214	25,927	26	1 (0.66 to 1.47)	1,935,389	122,252	549	4.49 (4.12 to 4.88)
BNT162b2 2nd dose	1,360,495	85,825	52	0.61 (0.45 to 0.79)	176,358	12,574	8	0.64 (0.27 to 1.25)	1,276,052	85,949	523	6.08 (5.57 to 6.63)
mRNA -1273 1st dose					645	48	0	0 (0 to 75.64)	242,770	17,550	39	2.22 (1.58 to 3.04)
mRNA -1273 2nd dose					71	5	0	0 (0 to 704.69)	181,905	9,704	24	2.47 (1.58 to 3.68)

Ad26.COV2.S					18,433	1,248	<5		125,015	5,935	18	3.03 (1.8 to 4.79)
**Myocardial infarction**												
ChAdOx1 1st dose	3,727,073	271,378	422	1.56 (1.41 to 1.71)	87,339	6,620	10	1.51 (0.72 to 2.78)				
ChAdOx1 2nd dose	1,155,608	56,294	85	1.51 (1.21 to 1.87)	17,915	1,253	<5					
BNT162b2 1st dose	1,783,357	135,356	268	1.98 (1.75 to 2.23)	341,848	25,748	34	1.32 (0.91 to 1.85)	1,956,402	123,866	289	2.33 (2.07 to 2.62)
BNT162b2 2nd dose	1,324,606	83,538	158	1.89 (1.61 to 2.21)	174,395	12,425	24	1.93 (1.24 to 2.87)	1,297,110	87,586	269	3.07 (2.72 to 3.46)
mRNA -1273 1st dose					645	48	0	0 (0 to 75.63)	242,726	17,546	30	1.71 (1.15 to 2.44)
mRNA -1273 2nd dose					71	5	0	0 (0 to 704.69)	181,881	9,716	21	2.16 (1.34 to 3.3)
Ad26.COV2.S					18,277	1,236	<5		125,219	5,951	10	1.68 (0.81 to 3.09)
**Intestinal infarction**												
ChAdOx1 1st dose	3,787,035	275,950	28	0.1 (0.07 to 0.15)	89,687	6,799	0	0 (0 to 0.54)				
ChAdOx1 2nd dose	1,193,764	58,255	5	0.09 (0.03 to 0.2)	18,768	1,316	0	0 (0 to 2.8)				
BNT162b2 1st dose	1,838,646	139,584	19	0.14 (0.08 to 0.21)	350,850	26,432	0	0 (0 to 0.14)	1,988,290	126,231	24	0.19 (0.12 to 0.28)
BNT162b2 2nd dose	1,367,612	86,300	10	0.12 (0.06 to 0.21)	181,693	12,970	0	0 (0 to 0.28)	1,326,443	89,712	20	0.22 (0.14 to 0.34)
mRNA -1273 1st dose					653	49	0	0 (0 to 74.7)	245,853	17,780	<5	
mRNA -1273 2nd dose					72	5	0	0 (0 to 694.52)	184,577	9,875	5	0.51 (0.16 to 1.18)
Ad26.COV2.S					18,758	1,271	0	0 (0 to 2.9)	126,855	6,048	<5	
**Arterial thromboembolism (ATE)**												
ChAdOx1 1st dose	3,716,038	270,539	629	2.32 (2.15 to 2.51)	85,890	6,509	21	3.23 (2 to 4.93)				
ChAdOx1 2nd dose	1,148,502	55,917	141	2.52 (2.12 to 2.97)	17,438	1,218	<5					
BNT162b2 1st dose	1,773,973	134,639	451	3.35 (3.05 to 3.67)	335,573	25,270	63	2.49 (1.92 to 3.19)	1,904,070	119,934	998	8.32 (7.81 to 8.85)
BNT162b2 2nd dose	1,317,134	83,041	232	2.79 (2.45 to 3.18)	169,391	12,055	31	2.57 (1.75 to 3.65)	1,247,356	83,871	918	10.95 (10.25 to 11.68)
mRNA -1273 1st dose					637	48	0	0 (0 to 76.59)	239,599	17,313	86	4.97 (3.97 to 6.13)
mRNA -1273 2nd dose					70	5	0	0 (0 to 715.16)	179,168	9,542	53	5.55 (4.16 to 7.27)
Ad26.COV2.S					17,966	1,213	<5		123,408	5,839	34	5.82 (4.03 to 8.14)

Supplementary Table 10. Crude incidence rate and corresponding 95% confidence intercals, US data

	US Hospital CDM				US Open Claims			
Vaccine	N	Person-year	Events	IR per 1,000 person-years (95% CI)	N	Person-year	Events	IR per 1,000 person-years (95% CI)
**thrombocytopenia**								
BNT162b2 1st dose	240,105	15,552	135	8.68 (7.28 to 10.27)	5,285,326	376,318	1,038	2.76 (2.59 to 2.93)
BNT162b2 2nd dose	72,551	5,182	86	16.6 (13.27 to 20.5)	2,863,353	212,064	754	3.56 (3.31 to 3.82)
mRNA-1273 1st dose	61,081	4,603	25	5.43 (3.51 to 8.02)	3,923,483	298,953	946	3.16 (2.97 to 3.37)
mRNA-1273 2nd dose	19,561	1,382	25	18.08 (11.7 to 26.7)	2,189,283	165,463	642	3.88 (3.59 to 4.19)
Ad26.COV2.S	1,645	114	6	52.28 (19.18 to 113.78)	628,294	46,997	170	3.62 (3.09 to 4.2)
**TTS_any**								
BNT162b2 1st dose					5,288,025	376,521	818	2.17 (2.03 to 2.33)
BNT162b2 2nd dose					2,865,177	212,203	595	2.8 (2.58 to 3.04)
mRNA-1273 1st dose					3,925,741	299,125	750	2.51 (2.33 to 2.69)
mRNA-1273 2nd dose					2,190,856	165,583	502	3.03 (2.77 to 3.31)
Ad26.COV2.S					628,572	47,019	146	3.11 (2.62 to 3.65)
**TTS_ATE**								
BNT162b2 1st dose	247,434	16,066	12	0.75 (0.39 to 1.3)	5,374,657	382,956	36	0.09 (0.07 to 0.13)
BNT162b2 2nd dose	76,551	5,475	6	1.1 (0.4 to 2.39)	2,921,124	216,416	32	0.15 (0.1 to 0.21)

mRNA-1273 1st dose	62,720	4,726	<5		3,999,861	304,784	41	0.13 (0.1 to 0.18)
mRNA-1273 2nd dose	20,392	1,442	<5		2,240,829	169,389	33	0.19 (0.13 to 0.27)
Ad26.COV2.S	1,712	119	<5		639,180	47,822	<5	
**TTS_CVST**								
BNT162b2 1st dose	247,714	16,085	0	0 (0 to 0.23)	5,377,723	383,187	0	0 (0 to 0.01)
BNT162b2 2nd dose	76,682	5,484	0	0 (0 to 0.67)	2,923,166	216,571	0	0 (0 to 0.02)
mRNA-1273 1st dose	62,781	4,731	0	0 (0 to 0.78)	4,002,519	304,987	0	0 (0 to 0.01)
mRNA-1273 2nd dose	20,431	1,445	0	0 (0 to 2.55)	2,242,605	169,524	0	0 (0 to 0.02)
Ad26.COV2.S	1,715	119	0	0 (0 to 30.87)	639,538	47,849	<5	
**TTS_DVT**								
BNT162b2 1st dose	247,507	16,070	7	0.44 (0.18 to 0.9)	5,375,843	383,046	25	0.07 (0.04 to 0.1)
BNT162b2 2nd dose	76,552	5,475	<5		2,921,944	216,478	15	0.07 (0.04 to 0.11)
mRNA-1273 1st dose	62,737	4,727	<5		4,001,015	304,872	20	0.07 (0.04 to 0.1)
mRNA-1273 2nd dose	20,406	1,443	<5		2,241,586	169,446	12	0.07 (0.04 to 0.12)
Ad26.COV2.S	1,715	119	0	0 (0 to 30.87)	639,334	47,833	6	0.13 (0.05 to 0.27)
**TTS_ATE**								
BNT162b2 1st dose	247,611	16,078	<5		5,376,277	383,078	18	0.05 (0.03 to 0.07)
BNT162b2 2nd dose	76,635	5,481	<5		2,922,248	216,501	14	0.06 (0.04 to 0.11)
mRNA-1273 1st dose	62,763	4,729	0	0 (0 to 0.78)	4,001,310	304,894	20	0.07 (0.04 to 0.1)
mRNA-1273 2nd dose	20,417	1,444	0	0 (0 to 2.55)	2,241,759	169,460	14	0.08 (0.05 to 0.14)
Ad26.COV2.S	1,715	119	<5		639,374	47,836	<5	
**TTS_MI**								
BNT162b2 1st dose	247,553	16,074	9	0.56 (0.26 to 1.06)	5,376,066	383,062	16	0.04 (0.02 to 0.07)
BNT162b2 2nd dose	76,609	5,479	5	0.91 (0.3 to 2.13)	2,922,006	216,483	20	0.09 (0.06 to 0.14)
mRNA-1273 1st dose	62,740	4,727	<5		4,001,023	304,873	20	0.07 (0.04 to 0.1)
mRNA-1273 2nd dose	20,408	1,443	<5		2,241,633	169,450	19	0.11 (0.07 to 0.18)
Ad26.COV2.S	1,712	119	<5		639,336	47,833	<5	
**TTS_PE**								
BNT162b2 1st dose	247,545	16,072	<5		5,376,230	383,074	19	0.05 (0.03 to 0.08)
BNT162b2 2nd dose	76,580	5,477	0	0 (0 to 0.67)	2,922,111	216,491	12	0.06 (0.03 to 0.1)
mRNA-1273 1st dose	62,749	4,728	0	0 (0 to 0.78)	4,001,176	304,884	13	0.04 (0.02 to 0.07)
mRNA-1273 2nd dose	20,417	1,444	<5		2,241,723	169,457	13	0.08 (0.04 to 0.13)
Ad26.COV2.S	1,713	119	0	0 (0 to 30.9)	639,368	47,836	6	0.13 (0.05 to 0.27)
**TTS_SVT**								
BNT162b2 1st dose	247,668	16,081	<5		5,377,449	383,166	<5	
BNT162b2 2nd dose	76,653	5,482	<5		2,922,992	216,558	<5	
mRNA-1273 1st dose	62,772	4,730	0	0 (0 to 0.78)	4,002,311	304,971	<5	
mRNA-1273 2nd dose	20,427	1,445	<5		2,242,473	169,514	5	0.03 (0.01 to 0.07)
Ad26.COV2.S	1,714	119	0	0 (0 to 30.89)	639,507	47,847	<5	
**TTS_VTE**								
BNT162b2 1st dose	247,444	16,065	8	0.5 (0.21 to 0.98)	5,375,270	383,003	32	0.08 (0.06 to 0.12)
BNT162b2 2nd dose	76,517	5,472	<5		2,921,557	216,448	16	0.07 (0.04 to 0.12)
mRNA-1273 1st dose	62,725	4,726	<5		4,000,511	304,834	23	0.08 (0.05 to 0.11)
mRNA-1273 2nd dose	20,402	1,443	<5		2,241,261	169,422	16	0.09 (0.05 to 0.15)
Ad26.COV2.S	1,713	119	0	0 (0 to 30.9)	639,270	47,828	11	0.23 (0.11 to 0.41)

**Cerebral venous sinus thrombosis**								
BNT162b2 1st dose	247,697	16,083	0	0 (0 to 0.23)	5,377,097	383,140	10	0.03 (0.01 to 0.05)
BNT162b2 2nd dose	76,675	5,484	0	0 (0 to 0.67)	2,922,724	216,537	9	0.04 (0.02 to 0.08)
mRNA-1273 1st dose	62,776	4,730	0	0 (0 to 0.78)	4,002,042	304,950	6	0.02 (0.01 to 0.04)
mRNA-1273 2nd dose	20,428	1,445	<5		2,242,315	169,502	<5	
Ad26.COV2.S	1,714	119	0	0 (0 to 30.89)	639,457	47,843	<5	
**Splanchnic and Visceral Thrombosis**								
BNT162b2 1st dose	247,470	16,067	<5		5,374,280	382,930	32	0.08 (0.06 to 0.12)
BNT162b2 2nd dose	76,533	5,473	5	0.91 (0.3 to 2.13)	2,920,875	216,398	21	0.1 (0.06 to 0.15)
mRNA-1273 1st dose	62,704	4,725	<5		3,999,804	304,780	42	0.14 (0.1 to 0.19)
mRNA-1273 2nd dose	20,394	1,442	<5		2,240,723	169,381	28	0.17 (0.11 to 0.24)
Ad26.COV2.S	1,713	119	0	0 (0 to 30.91)	639,112	47,817	10	0.21 (0.1 to 0.38)
**Deep vein thrombosis**								
BNT162b2 1st dose	243,746	15,808	45	2.85 (2.08 to 3.81)	5,288,296	376,489	787	2.09 (1.95 to 2.24)
BNT162b2 2nd dose	74,589	5,332	41	7.69 (5.52 to 10.43)	2,862,601	211,990	508	2.4 (2.19 to 2.61)
mRNA-1273 1st dose	61,716	4,650	16	3.44 (1.97 to 5.59)	3,922,758	298,892	674	2.25 (2.09 to 2.43)
mRNA-1273 2nd dose	19,885	1,405	18	12.8 (7.59 to 20.23)	2,187,848	165,348	453	2.74 (2.49 to 3)
Ad26.COV2.S	1,680	116	<5		628,004	46,974	121	2.58 (2.14 to 3.08)
**Pulmonary embolism**								
BNT162b2 1st dose	244,991	15,895	36	2.26 (1.59 to 3.14)	5,324,735	379,216	598	1.58 (1.45 to 1.71)
BNT162b2 2nd dose	75,211	5,378	22	4.09 (2.56 to 6.19)	2,887,007	213,839	351	1.64 (1.47 to 1.82)
mRNA-1273 1st dose	62,104	4,679	10	2.14 (1.02 to 3.93)	3,955,656	301,407	465	1.54 (1.41 to 1.69)
mRNA-1273 2nd dose	20,064	1,418	13	9.16 (4.88 to 15.67)	2,210,655	167,087	317	1.9 (1.69 to 2.12)
Ad26.COV2.S	1,684	117	<5		632,835	47,339	105	2.22 (1.81 to 2.69)
**Venous thrombo-embolism**								
BNT162b2 1st dose	242,061	15,691	67	4.27 (3.31 to 5.42)	5,256,785	374,134	1,146	3.06 (2.89 to 3.25)
BNT162b2 2nd dose	73,682	5,266	52	9.87 (7.37 to 12.95)	2,841,124	210,370	726	3.45 (3.2 to 3.71)
mRNA-1273 1st dose	61,319	4,620	22	4.76 (2.98 to 7.21)	3,895,067	296,777	971	3.27 (3.07 to 3.48)
mRNA-1273 2nd dose	19,667	1,390	25	17.99 (11.64 to 26.55)	2,169,182	163,926	646	3.94 (3.64 to 4.26)
Ad26.COV2.S	1,659	115	<5		624,003	46,670	190	4.07 (3.51 to 4.69)
**Ischaemic stroke**								
BNT162b2 1st dose	243,522	15,794	53	3.36 (2.51 to 4.39)	5,252,695	373,795	1,213	3.25 (3.07 to 3.43)
BNT162b2 2nd dose	74,521	5,327	45	8.45 (6.16 to 11.3)	2,837,126	210,050	841	4 (3.74 to 4.28)
mRNA-1273 1st dose	61,702	4,649	19	4.09 (2.46 to 6.38)	3,889,140	296,321	1,160	3.91 (3.69 to 4.15)
mRNA-1273 2nd dose	19,851	1,402	12	8.55 (4.42 to 14.94)	2,165,395	163,635	800	4.89 (4.56 to 5.24)
Ad26.COV2.S	1,664	116	<5		623,397	46,622	193	4.14 (3.58 to 4.77)
**Myocardial infarction**								
BNT162b2 1st dose	243,360	15,786	61	3.86 (2.96 to 4.96)	5,281,985	375,988	1,003	2.67 (2.51 to 2.84)
BNT162b2 2nd dose	74,570	5,330	48	9 (6.64 to 11.94)	2,857,066	211,557	734	3.47 (3.22 to 3.73)
mRNA-1273 1st dose	61,614	4,643	23	4.95 (3.14 to 7.43)	3,909,897	297,907	1,024	3.44 (3.23 to 3.65)

mRNA-1273 2nd dose	19,816	1,400	22	15.7 (9.84 to 23.78)	2,178,281	164,615	691	4.2 (3.89 to 4.52)
Ad26.COV2.S	1,672	116	<5		625,169	46,757	168	3.59 (3.07 to 4.18)
**Intestinal infarction**								
BNT162b2 1st dose	247,266	16,053	<5		5,368,328	382,481	99	0.26 (0.21 to 0.32)
BNT162b2 2nd dose	76,446	5,467	7	1.28 (0.51 to 2.64)	2,916,832	216,091	70	0.32 (0.25 to 0.41)
mRNA-1273 1st dose	62,682	4,723	<5		3,993,545	304,301	95	0.31 (0.25 to 0.38)
mRNA-1273 2nd dose	20,371	1,440	0	0 (0 to 2.56)	2,236,418	169,052	103	0.61 (0.5 to 0.74)
Ad26.COV2.S	1,714	119	0	0 (0 to 30.89)	638,258	47,752	7	0.15 (0.06 to 0.3)
**Arterial thromboembolism**								
BNT162b2 1st dose	239,472	15,517	140	9.02 (7.59 to 10.65)	5,172,486	367,768	4,666	12.69 (12.33 to 13.06)
BNT162b2 2nd dose	72,596	5,187	110	21.2 (17.43 to 25.56)	2,781,792	205,853	3,211	15.6 (15.06 to 16.15)
mRNA-1273 1st dose	60,601	4,566	54	11.83 (8.88 to 15.43)	3,810,079	290,277	4,615	15.9 (15.44 to 16.36)
mRNA-1273 2nd dose	19,275	1,361	40	29.38 (20.99 to 40.01)	2,110,385	159,437	2,926	18.35 (17.69 to 19.03)
Ad26.COV2.S	1,625	113	<5		610,896	45,673	720	15.76 (14.63 to 16.96)

Supplementary Table 11. Incidence rates before- and after- propensity score matching.

Database	Vaccine	IR pre-matching	IR after-matching		Database	Vaccine	IR pre-matching	IR after-matching
**Thrombocytopenia**					**Intestinal infarction**			
CPRD_Aurum	ChAdOx1 1st dose	4.78 (4.52 to 5.05)	6.06 (5.65 to 6.48)		CPRD_Aurum	ChAdOx1 1st dose	0.1 (0.07 to 0.15)	0.15 (0.1 to 0.23)
CPRD_Aurum	ChAdOx1 2nd dose	6.4 (5.75 to 7.11)	5.98 (5.23 to 6.8)		CPRD_Aurum	BNT162b2 1st dose	0.14 (0.08 to 0.21)	0.15 (0.08 to 0.24)
CPRD_Aurum	BNT162b2 1st dose	4.9 (4.53 to 5.29)	4.89 (4.45 to 5.37)		CPRD_Aurum	BNT162b2 2nd dose	0.12 (0.06 to 0.21)	0.12 (0.05 to 0.24)
CPRD_Aurum	BNT162b2 2nd dose	5.7 (5.19 to 6.25)	5.75 (5.16 to 6.39)		US_Open_Claims	BNT162b2 1st dose	0.26 (0.21 to 0.32)	0.3 (0.23 to 0.4)
France_LPD	ChAdOx1 1st dose	3.7 (1.49 to 7.62)	4.56 (1.48 to 10.63)		US_Open_Claims	mRNA-1273 1st dose	0.31 (0.25 to 0.38)	0.31 (0.24 to 0.41)
Germany_DA	ChAdOx1 1st dose	5.71 (4.02 to 7.87)	5.61 (3.91 to 7.81)		US_Open_Claims	Ad26.COV2.S	0.15 (0.06 to 0.3)	0.15 (0.06 to 0.3)
Germany_DA	BNT162b2 1st dose	4.61 (3.81 to 5.52)	5.03 (3.97 to 6.27)		**ATE**			
Germany_DA	BNT162b2 2nd dose	4.87 (3.7 to 6.28)	2.8 (1.4 to 5.02)		CPRD_Aurum	ChAdOx1 1st dose	2.32 (2.15 to 2.51)	2.97 (2.69 to 3.27)
Germany_DA	Ad26.COV2.S	10.68 (5.69 to 18.27)	9.89 (5.11 to 17.28)		CPRD_Aurum	ChAdOx1 2nd dose	2.52 (2.12 to 2.97)	2.54 (2.07 to 3.09)
IPCI	BNT162b2 1st dose	3.07 (2.2 to 4.16)	3.2 (1.6 to 5.73)		CPRD_Aurum	BNT162b2 1st dose	3.35 (3.05 to 3.67)	3.57 (3.19 to 3.97)
SIDIAP	BNT162b2 1st dose	13.81 (13.12 to 14.52)	9.88 (8.55 to 11.36)		CPRD_Aurum	BNT162b2 2nd dose	2.79 (2.45 to 3.18)	2.46 (2.08 to 2.88)
SIDIAP	Ad26.COV2.S	10.49 (7.94 to 13.59)	9.73 (7.2 to 12.86)		Germany_DA	ChAdOx1 1st dose	3.23 (2 to 4.93)	3.03 (1.83 to 4.74)
US_Open_Claims	BNT162b2 1st dose	2.76 (2.59 to 2.93)	2.72 (2.48 to 2.98)		Germany_DA	BNT162b2 1st dose	2.49 (1.92 to 3.19)	2.83 (2.06 to 3.8)
US_Open_Claims	mRNA-1273 1st dose	3.16 (2.97 to 3.37)	2.85 (2.6 to 3.12)		Germany_DA	BNT162b2 2nd dose	2.57 (1.75 to 3.65)	1.79 (0.72 to 3.69)
US_Open_Claims	Ad26.COV2.S	3.62 (3.09 to 4.2)	3.62 (3.09 to 4.2)		IPCI	ChAdOx1 1st dose	4.28 (2.68 to 6.48)	3.23 (1.05 to 7.53)
**TTS_any**					IPCI	BNT162b2 1st dose	5.49 (4.3 to 6.92)	5.34 (3.16 to 8.44)
CPRD_Aurum	ChAdOx1 1st dose	0.72 (0.62 to 0.82)	0.84 (0.7 to 1)		IPCI	BNT162b2 2nd dose	6.35 (4.26 to 9.13)	4.24 (1.55 to 9.22)
CPRD_Aurum	ChAdOx1 2nd dose	0.88 (0.66 to 1.16)	0.93 (0.65 to 1.27)		SIDIAP	BNT162b2 1st dose	8.32 (7.81 to 8.85)	5.55 (4.6 to 6.65)
CPRD_Aurum	BNT162b2 1st dose	0.71 (0.57 to 0.86)	0.67 (0.52 to 0.86)		SIDIAP	Ad26.COV2.S	5.82 (4.03 to 8.14)	6.27 (4.34 to 8.76)
CPRD_Aurum	BNT162b2 2nd dose	0.79 (0.62 to 1.01)	0.65 (0.47 to 0.88)		US_Open_Claims	BNT162b2 1st dose	12.69 (12.33 to 13.06)	13.26 (12.72 to 13.83)
US_Open_Claims	BNT162b2 1st dose	2.17 (2.03 to 2.33)	2.19 (1.97 to 2.42)		US_Open_Claims	mRNA-1273 1st dose	15.9 (15.44 to 16.37)	13.6 (13.04 to 14.17)
US_Open_Claims	mRNA-1273 1st dose	2.51 (2.33 to 2.69)	2.24 (2.02 to 2.47)		US_Open_Claims	Ad26.COV2.S	15.76 (14.63 to 16.96)	15.76 (14.63 to 16.96)
US_Open_Claims	Ad26.COV2.S	3.11 (2.62 to 3.65)	3.11 (2.62 to 3.65)		**SVT**			
**TTS_ATE**					CPRD_Aurum_202106	ChAdOx1 1st dose	0.06 (0.04 to 0.1)	0.1 (0.05 to 0.16)

CPRD_Aurum	ChAdOx1 1st dose	0.03 (0.01 to 0.05)	0.03 (0.01 to 0.08)		US_Open_Claims	BNT162b2 1st dose	0.08 (0.06 to 0.12)	0.11 (0.07 to 0.17)
US_Open_Claims	BNT162b2 1st dose	0.09 (0.07 to 0.13)	0.09 (0.05 to 0.15)		US_Open_Claims	mRNA-1273 1st dose	0.14 (0.1 to 0.19)	0.1 (0.06 to 0.16)
US_Open_Claims	mRNA-1273 1st dose	0.13 (0.1 to 0.18)	0.14 (0.09 to 0.21)		US_Open_Claims	Ad26.COV2.S	0.21 (0.1 to 0.38)	0.21 (0.1 to 0.38)
**TTS_DVT**					**CVST**			
CPRD_Aurum	BNT162b2 2nd dose	0.08 (0.03 to 0.17)	0.08 (0.03 to 0.18)		US_Open_Claims	BNT162b2 1st dose	0.03 (0.01 to 0.05)	0.03 (0.01 to 0.07)
US_Open_Claims	BNT162b2 1st dose	0.07 (0.04 to 0.1)	0.06 (0.03 to 0.1)		**DVT**			
US_Open_Claims	mRNA-1273 1st dose	0.07 (0.04 to 0.1)	0.07 (0.04 to 0.12)		CPRD_Aurum	ChAdOx1 1st dose	1.23 (1.1 to 1.37)	1.36 (1.17 to 1.56)
US_Open_Claims	Ad26.COV2.S	0.13 (0.05 to 0.27)	0.13 (0.05 to 0.27)		CPRD_Aurum	ChAdOx1 2nd dose	1.51 (1.21 to 1.86)	1.51 (1.15 to 1.93)
**TTS_isc stroke**					CPRD_Aurum	BNT162b2 1st dose	1.72 (1.51 to 1.96)	1.59 (1.35 to 1.87)
US_Open_Claims	BNT162b2 1st dose	0.05 (0.03 to 0.07)	0.05 (0.02 to 0.09)		CPRD_Aurum	BNT162b2 2nd dose	1.69 (1.42 to 1.99)	1.51 (1.23 to 1.85)
US_Open_Claims	mRNA-1273 1st dose	0.07 (0.04 to 0.1)	0.06 (0.03 to 0.11)		Germany_DA	ChAdOx1 1st dose	3.13 (1.94 to 4.79)	3.25 (2.01 to 4.97)
**TTS_MI**					Germany_DA	BNT162b2 1st dose	1.27 (0.87 to 1.78)	1.31 (0.81 to 2)
US_Open_Claims	BNT162b2 1st dose	0.04 (0.02 to 0.07)	0.05 (0.02 to 0.09)		IPCI	BNT162b2 1st dose	1.48 (0.91 to 2.29)	1.44 (0.47 to 3.36)
US_Open_Claims	mRNA-1273 1st dose	0.07 (0.04 to 0.1)	0.08 (0.04 to 0.14)		SIDIAP	BNT162b2 1st dose	1.75 (1.53 to 2)	1.5 (1.03 to 2.1)
**TTS_PE**					SIDIAP	Ad26.COV2.S	1.66 (0.8 to 3.06)	1.79 (0.86 to 3.29)
CPRD_Aurum	ChAdOx1 1st dose	0.03 (0.01 to 0.05)	0.03 (0.01 to 0.08)		US_Open_Claims	BNT162b2 1st dose	2.09 (1.95 to 2.24)	2.01 (1.8 to 2.23)
US_Open_Claims	BNT162b2 1st dose	0.05 (0.03 to 0.08)	0.05 (0.02 to 0.09)		US_Open_Claims	mRNA-1273 1st dose	2.25 (2.09 to 2.43)	1.98 (1.77 to 2.2)
US_Open_Claims	mRNA-1273 1st dose	0.04 (0.02 to 0.07)	0.04 (0.02 to 0.08)		US_Open_Claims	Ad26.COV2.S	2.58 (2.14 to 3.08)	2.58 (2.14 to 3.08)
US_Open_Claims	Ad26.COV2.S	0.13 (0.05 to 0.27)	0.13 (0.05 to 0.27)		**PE**			
**TTS_VTE**					CPRD_Aurum	ChAdOx1 1st dose	1.41 (1.28 to 1.56)	1.88 (1.66 to 2.12)
CPRD_Aurum	ChAdOx1 1st dose	0.05 (0.03 to 0.09)	0.05 (0.02 to 0.1)		CPRD_Aurum	ChAdOx1 2nd dose	1.22 (0.95 to 1.54)	1.3 (0.97 to 1.7)
CPRD_Aurum	BNT162b2 2nd dose	0.1 (0.05 to 0.2)	0.09 (0.03 to 0.2)		CPRD_Aurum	BNT162b2 1st dose	2 (1.77 to 2.25)	2.08 (1.8 to 2.39)
US_Open_Claims	BNT162b2 1st dose	0.08 (0.06 to 0.12)	0.07 (0.04 to 0.13)		CPRD_Aurum	BNT162b2 2nd dose	1.43 (1.19 to 1.71)	1.44 (1.16 to 1.77)
US_Open_Claims	mRNA-1273 1st dose	0.08 (0.05 to 0.11)	0.08 (0.04 to 0.14)		Germany_DA	ChAdOx1 1st dose	0.89 (0.33 to 1.94)	0.93 (0.34 to 2.02)
US_Open_Claims	Ad26.COV2.S	0.23 (0.11 to 0.41)	0.23 (0.11 to 0.41)		Germany_DA	BNT162b2 1st dose	1.38 (0.97 to 1.92)	1.24 (0.76 to 1.92)
**Ischemic stroke**					Germany_DA	BNT162b2 2nd dose	1.58 (0.96 to 2.44)	2.17 (0.99 to 4.13)
CPRD_Aurum	ChAdOx1 1st dose	0.35 (0.28 to 0.42)	0.52 (0.41 to 0.65)		IPCI	BNT162b2 1st dose	1.78 (1.14 to 2.65)	1.73 (0.63 to 3.76)
CPRD_Aurum	ChAdOx1 2nd dose	0.57 (0.39 to 0.8)	0.56 (0.35 to 0.84)		SIDIAP	BNT162b2 1st dose	1.41 (1.21 to 1.63)	0.63 (0.35 to 1.06)
CPRD_Aurum	BNT162b2 1st dose	0.85 (0.7 to 1.02)	0.79 (0.63 to 0.99)		SIDIAP	Ad26.COV2.S	0.83 (0.27 to 1.94)	0.89 (0.29 to 2.09)
CPRD_Aurum	BNT162b2 2nd dose	0.61 (0.45 to 0.79)	0.43 (0.29 to 0.63)		US_Open_Claims	BNT162b2 1st dose	1.58 (1.45 to 1.71)	1.44 (1.26 to 1.63)
Germany_DA	ChAdOx1 1st dose	1.95 (1.04 to 3.33)	1.71 (0.85 to 3.06)		US_Open_Claims	mRNA-1273 1st dose	1.54 (1.41 to 1.69)	1.33 (1.16 to 1.51)
Germany_DA	BNT162b2 1st dose	1 (0.66 to 1.47)	0.94 (0.53 to 1.55)		US_Open_Claims	Ad26.COV2.S	2.22 (1.81 to 2.69)	2.22 (1.81 to 2.69)
SIDIAP	BNT162b2 1st dose	4.49 (4.12 to 4.88)	2.8 (2.15 to 3.6)		**VTE**			
SIDIAP	Ad26.COV2.S	3.03 (1.8 to 4.79)	3.27 (1.94 to 5.16)		CPRD_Aurum	ChAdOx1 1st dose	2.42 (2.24 to 2.62)	2.98 (2.7 to 3.28)
US_Open_Claims	BNT162b2 1st dose	3.25 (3.07 to 3.44)	3.15 (2.89 to 3.43)		CPRD_Aurum	ChAdOx1 2nd dose	2.54 (2.14 to 2.99)	2.63 (2.15 to 3.18)
US_Open_Claims	mRNA-1273 1st dose	3.92 (3.7 to 4.15)	3.16 (2.9 to 3.44)		CPRD_Aurum	BNT162b2 1st dose	3.41 (3.11 to 3.74)	3.37 (3 to 3.76)
US_Open_Claims	Ad26.COV2.S	4.14 (3.58 to 4.77)	4.14 (3.58 to 4.77)		CPRD_Aurum	BNT162b2 2nd dose	2.96 (2.61 to 3.36)	2.85 (2.45 to 3.3)
**MI**					Germany_DA	ChAdOx1 1st dose	3.76 (2.43 to 5.55)	3.91 (2.53 to 5.77)
CPRD_Aurum	ChAdOx1 1st dose	1.56 (1.41 to 1.71)	2.01 (1.78 to 2.26)		Germany_DA	BNT162b2 1st dose	2.3 (1.75 to 2.97)	2.52 (1.8 to 3.43)
CPRD_Aurum	ChAdOx1 2nd dose	1.51 (1.21 to 1.87)	1.53 (1.17 to 1.96)		Germany_DA	BNT162b2 2nd dose	3.47 (2.51 to 4.67)	3.93 (2.25 to 6.38)
CPRD_Aurum	BNT162b2 1st dose	1.98 (1.75 to 2.23)	2.15 (1.87 to 2.47)		Germany_DA	Ad26.COV2.S	6.45 (2.78 to 12.7)	6.47 (2.79 to 12.74)
CPRD_Aurum	BNT162b2 2nd dose	1.89 (1.61 to 2.21)	1.74 (1.43 to 2.1)		IPCI	ChAdOx1 1st dose	3.82 (2.33 to 5.9)	3.81 (1.4 to 8.29)
Germany_DA	ChAdOx1 1st dose	1.51 (0.72 to 2.78)	1.57 (0.75 to 2.89)		IPCI	BNT162b2 1st dose	2.99 (2.14 to 4.07)	3.2 (1.6 to 5.72)
Germany_DA	BNT162b2 1st dose	1.32 (0.91 to 1.85)	1.64 (1.07 to 2.4)		IPCI	BNT162b2 2nd dose	2.99 (1.63 to 5.01)	4.16 (1.53 to 9.05)
IPCI	BNT162b2 1st dose	3.1 (2.22 to 4.2)	2.64 (1.21 to 5.01)		SIDIAP	BNT162b2 1st dose	2.88 (2.59 to 3.19)	1.91 (1.38 to 2.59)
IPCI	BNT162b2 2nd dose	3.46 (1.98 to 5.62)	3.49 (1.13 to 8.15)		SIDIAP	Ad26.COV2.S	2.34 (1.28 to 3.92)	2.52 (1.38 to 4.22)
SIDIAP	BNT162b2 1st dose	2.33 (2.07 to 2.62)	1.74 (1.23 to 2.39)		US_Open_Claims	BNT162b2 1st dose	3.06 (2.89 to 3.25)	2.95 (2.7 to 3.22)
SIDIAP	Ad26.COV2.S	1.68 (0.81 to 3.09)	1.81 (0.87 to 3.33)		US_Open_Claims	mRNA-1273 1st dose	3.27 (3.07 to 3.48)	2.9 (2.64 to 3.16)
US_Open_Claims	BNT162b2 1st dose	2.67 (2.5 to 2.84)	2.74 (2.5 to 3)		US_Open_Claims	Ad26.COV2.S	4.07 (3.51 to 4.69)	4.07 (3.51 to 4.69)
US_Open_Claims	mRNA-1273 1st dose	3.44 (3.23 to 3.65)	3.03 (2.78 to 3.31)					
US_Open_Claims	Ad26.COV2.S	3.59 (3.07 to 4.18)	3.59 (3.07 to 4.18)					

Supplementary Table 12. Age and sex stratified incidence rate ratio, CPRD UK.

Target vs. Comparator	Subgroup	MDRR	IRR	calibrated IRR	NCO eligibility
**CPRD_Aurum_202106**					
**ATE**					
ChAdOx1 vs. BNT162b2 1st dose	age group: 40 - 49	3.92	1.22 (0.48 to 3.53)	1.59 (0.62 to 4.13)	1
ChAdOx1 vs. BNT162b2 1st dose	age group: 50 - 59	1.82	0.57 (0.38 to 0.86)	0.69 (0.46 to 1.05)	1
ChAdOx1 vs. BNT162b2 1st dose	age group: 60 - 69	1.51	0.75 (0.57 to 1)	0.84 (0.57 to 1.24)	1
ChAdOx1 vs. BNT162b2 1st dose	age group: 70 - 79	1.44	0.74 (0.58 to 0.95)	0.95 (0.73 to 1.22)	1
ChAdOx1 vs. BNT162b2 1st dose	age group: 80 - 89	1.64	0.86 (0.6 to 1.22)	0.84 (0.58 to 1.23)	1
ChAdOx1 vs. BNT162b2 1st dose	gender = MALE	1.31	0.66 (0.55 to 0.79)	**0.75 (0.61 to 0.92)**	1
ChAdOx1 vs. BNT162b2 1st dose	gender = FEMALE	1.41	0.79 (0.63 to 1.01)	0.97 (0.76 to 1.23)	1
ChAdOx1 vs. BNT162b2 2nd dose	age group: 70 - 79	1.72	1.24 (0.85 to 1.81)	1.12 (0.75 to 1.67)	1
ChAdOx1 vs. BNT162b2 2nd dose	age group: 80 - 89	2	1.28 (0.79 to 2.02)	1.07 (0.62 to 1.86)	1
ChAdOx1 vs. BNT162b2 2nd dose	gender = MALE	1.63	1.32 (0.94 to 1.84)	1.11 (0.78 to 1.58)	1
ChAdOx1 vs. BNT162b2 2nd dose	gender = FEMALE	1.74	1.3 (0.88 to 1.9)	0.96 (0.64 to 1.46)	1
**DVT**					
ChAdOx1 vs. BNT162b2 1st dose	age group: 40 - 49	3.93	0.55 (0.22 to 1.4)	0.72 (0.28 to 1.82)	1
ChAdOx1 vs. BNT162b2 1st dose	age group: 50 - 59	2.2	0.54 (0.32 to 0.93)	0.66 (0.38 to 1.14)	1
ChAdOx1 vs. BNT162b2 1st dose	age group: 60 - 69	2.02	0.78 (0.49 to 1.28)	0.88 (0.51 to 1.5)	1
ChAdOx1 vs. BNT162b2 1st dose	age group: 70 - 79	1.71	0.71 (0.49 to 1.03)	0.91 (0.62 to 1.31)	1
ChAdOx1 vs. BNT162b2 1st dose	age group: 80 - 89	2.05	1.14 (0.69 to 1.91)	1.11 (0.66 to 1.87)	1
ChAdOx1 vs. BNT162b2 1st dose	gender = MALE	1.53	0.76 (0.57 to 1.02)	0.87 (0.64 to 1.18)	1
ChAdOx1 vs. BNT162b2 1st dose	gender = FEMALE	1.6	0.7 (0.51 to 0.97)	0.86 (0.62 to 1.19)	1
ChAdOx1 vs. BNT162b2 2nd dose	age group: 70 - 79	2.17	1.12 (0.64 to 1.9)	1.01 (0.57 to 1.79)	1
ChAdOx1 vs. BNT162b2 2nd dose	age group: 80 - 89	2.45	1.21 (0.64 to 2.21)	1.01 (0.5 to 2.02)	1
ChAdOx1 vs. BNT162b2 2nd dose	gender = MALE	2.01	1.41 (0.87 to 2.27)	1.19 (0.73 to 1.95)	1
ChAdOx1 vs. BNT162b2 2nd dose	gender = FEMALE	1.85	1.06 (0.67 to 1.63)	0.78 (0.49 to 1.26)	1
**Intestinal infarction**					
ChAdOx1 vs. BNT162b2 1st dose	gender = MALE	4.34	0.82 (0.29 to 2.37)	0.93 (0.33 to 2.65)	1
ChAdOx1 vs. BNT162b2 1st dose	gender = FEMALE	3.89	0.78 (0.32 to 2.09)	0.95 (0.39 to 2.31)	1
**Ischemic stroke**					
ChAdOx1 vs. BNT162b2 1st dose	age group: 50 - 59	4.37	0.62 (0.24 to 1.74)	0.75 (0.28 to 2)	1
ChAdOx1 vs. BNT162b2 1st dose	age group: 60 - 69	2.78	0.62 (0.32 to 1.25)	0.7 (0.34 to 1.44)	1
ChAdOx1 vs. BNT162b2 1st dose	age group: 70 - 79	2.12	0.55 (0.33 to 0.91)	0.7 (0.42 to 1.16)	1
ChAdOx1 vs. BNT162b2 1st dose	age group: 80 - 89	3	0.9 (0.39 to 1.93)	0.88 (0.37 to 2.07)	1
ChAdOx1 vs. BNT162b2 1st dose	gender = MALE	1.97	0.47 (0.3 to 0.75)	**0.54 (0.34 to 0.87)**	1
ChAdOx1 vs. BNT162b2 1st dose	gender = FEMALE	1.94	0.65 (0.42 to 1.03)	0.8 (0.51 to 1.25)	1
ChAdOx1 vs. BNT162b2 2nd dose	age group: 70 - 79	3.4	1.61 (0.68 to 4)	1.45 (0.6 to 3.49)	1
ChAdOx1 vs. BNT162b2 2nd dose	age group: 80 - 89	3.66	1.21 (0.47 to 2.96)	1.01 (0.38 to 2.72)	1
ChAdOx1 vs. BNT162b2 2nd dose	gender = MALE	2.91	1.61 (0.76 to 3.37)	1.36 (0.64 to 2.88)	1
ChAdOx1 vs. BNT162b2 2nd dose	gender = FEMALE	3.47	1.35 (0.55 to 3.15)	1 (0.4 to 2.47)	1
**MI**					
ChAdOx1 vs. BNT162b2 1st dose	age group: 50 - 59	2.16	0.55 (0.33 to 0.94)	0.67 (0.39 to 1.14)	1

ChAdOx1 vs. BNT162b2 1st dose	age group: 60 - 69	1.67	0.82 (0.58 to 1.18)	0.92 (0.59 to 1.43)	1
ChAdOx1 vs. BNT162b2 1st dose	age group: 70 - 79	1.59	0.83 (0.61 to 1.14)	1.06 (0.77 to 1.45)	1
ChAdOx1 vs. BNT162b2 1st dose	age group: 80 - 89	1.85	0.95 (0.62 to 1.47)	0.93 (0.59 to 1.46)	1
ChAdOx1 vs. BNT162b2 1st dose	gender = MALE	1.4	0.75 (0.6 to 0.95)	0.86 (0.67 to 1.1)	1
ChAdOx1 vs. BNT162b2 1st dose	gender = FEMALE	1.55	0.84 (0.62 to 1.13)	1.02 (0.75 to 1.38)	1
ChAdOx1 vs. BNT162b2 2nd dose	age group: 70 - 79	1.93	1.04 (0.65 to 1.66)	0.94 (0.58 to 1.54)	1
ChAdOx1 vs. BNT162b2 2nd dose	age group: 80 - 89	2.46	1.4 (0.75 to 2.47)	1.17 (0.59 to 2.29)	1
ChAdOx1 vs. BNT162b2 2nd dose	gender = MALE	1.8	1.19 (0.78 to 1.79)	1 (0.65 to 1.54)	1
ChAdOx1 vs. BNT162b2 2nd dose	gender = FEMALE	2	1.15 (0.7 to 1.85)	0.85 (0.5 to 1.44)	1
**PE**					
ChAdOx1 vs. BNT162b2 1st dose	age group: 40 - 49	3.39	0.59 (0.25 to 1.36)	0.77 (0.33 to 1.82)	1
ChAdOx1 vs. BNT162b2 1st dose	age group: 50 - 59	2.64	0.84 (0.44 to 1.69)	1.02 (0.54 to 1.95)	1
ChAdOx1 vs. BNT162b2 1st dose	age group: 60 - 69	1.82	0.66 (0.44 to 0.99)	0.73 (0.46 to 1.18)	1
ChAdOx1 vs. BNT162b2 1st dose	age group: 70 - 79	1.54	0.86 (0.64 to 1.16)	1.1 (0.81 to 1.48)	1
ChAdOx1 vs. BNT162b2 1st dose	age group: 80 - 89	1.81	1.09 (0.72 to 1.67)	1.07 (0.69 to 1.65)	1
ChAdOx1 vs. BNT162b2 1st dose	gender = MALE	1.48	0.76 (0.58 to 1)	0.87 (0.65 to 1.15)	1
ChAdOx1 vs. BNT162b2 1st dose	gender = FEMALE	1.45	0.79 (0.62 to 1.03)	0.97 (0.75 to 1.26)	1
ChAdOx1 vs. BNT162b2 2nd dose	age group: 70 - 79	2.14	1.12 (0.66 to 1.93)	1.01 (0.58 to 1.75)	1
ChAdOx1 vs. BNT162b2 2nd dose	age group: 80 - 89	2.35	0.73 (0.36 to 1.37)	0.61 (0.28 to 1.31)	1
ChAdOx1 vs. BNT162b2 2nd dose	gender = MALE	2.02	1.43 (0.87 to 2.32)	1.2 (0.73 to 1.98)	1
ChAdOx1 vs. BNT162b2 2nd dose	gender = FEMALE	1.94	0.9 (0.54 to 1.45)	0.66 (0.39 to 1.13)	1
**Thrombocytopenia**					
ChAdOx1 vs. BNT162b2 1st dose	age group: 30 - 39	2.31	1.21 (0.67 to 2.29)	1.61 (0.74 to 3.48)	1
ChAdOx1 vs. BNT162b2 1st dose	age group: 40 - 49	1.86	1.39 (0.9 to 2.19)	**1.82 (1.15 to 2.88)**	1
ChAdOx1 vs. BNT162b2 1st dose	age group: 50 - 59	1.6	0.87 (0.63 to 1.2)	1.05 (0.75 to 1.47)	1
ChAdOx1 vs. BNT162b2 1st dose	age group: 60 - 69	1.46	1.04 (0.8 to 1.36)	1.16 (0.8 to 1.69)	1
ChAdOx1 vs. BNT162b2 1st dose	age group: 70 - 79	1.34	1.09 (0.89 to 1.34)	**1.39 (1.12 to 1.73)**	1
ChAdOx1 vs. BNT162b2 1st dose	age group: 80 - 89	1.47	1.22 (0.93 to 1.6)	1.19 (0.88 to 1.6)	1
ChAdOx1 vs. BNT162b2 1st dose	gender = MALE	1.24	0.98 (0.85 to 1.14)	1.12 (0.94 to 1.34)	1
ChAdOx1 vs. BNT162b2 1st dose	gender = FEMALE	1.29	1.23 (1.02 to 1.47)	**1.49 (1.23 to 1.81)**	1
ChAdOx1 vs. BNT162b2 2nd dose	age group: 70 - 79	1.48	1.15 (0.87 to 1.51)	1.03 (0.77 to 1.39)	1
ChAdOx1 vs. BNT162b2 2nd dose	age group: 80 - 89	1.58	1 (0.72 to 1.37)	0.83 (0.54 to 1.28)	1
ChAdOx1 vs. BNT162b2 2nd dose	gender = MALE	1.37	1.04 (0.83 to 1.3)	0.88 (0.69 to 1.12)	1
ChAdOx1 vs. BNT162b2 2nd dose	gender = FEMALE	1.45	1.44 (1.12 to 1.85)	1.06 (0.79 to 1.42)	1
**TTS_any**					
ChAdOx1 vs. BNT162b2 1st dose	age group: 40 - 49	4.27	1.55 (0.55 to 4.72)	2.03 (0.71 to 5.77)	1
ChAdOx1 vs. BNT162b2 1st dose	age group: 50 - 59	3.43	1.28 (0.53 to 3.39)	1.55 (0.64 to 3.72)	1
ChAdOx1 vs. BNT162b2 1st dose	age group: 60 - 69	2.59	0.96 (0.5 to 1.93)	1.07 (0.53 to 2.15)	1
ChAdOx1 vs. BNT162b2 1st dose	age group: 70 - 79	2.23	1.05 (0.6 to 1.91)	1.34 (0.77 to 2.34)	1
ChAdOx1 vs. BNT162b2 1st dose	age group: 80 - 89	3	1.08 (0.49 to 2.39)	1.05 (0.48 to 2.32)	1
ChAdOx1 vs. BNT162b2 1st dose	gender = MALE	1.8	1.24 (0.82 to 1.92)	1.41 (0.92 to 2.17)	1
ChAdOx1 vs. BNT162b2 1st dose	gender = FEMALE	1.89	0.87 (0.56 to 1.35)	1.05 (0.68 to 1.63)	1
ChAdOx1 vs. BNT162b2 2nd dose	age group: 70 - 79	2.55	1.36 (0.7 to 2.74)	1.23 (0.63 to 2.41)	1

ChAdOx1 vs. BNT162b2 2nd dose	age group: 80 - 89	3.43	1.22 (0.49 to 2.8)	1.02 (0.39 to 2.65)	1
ChAdOx1 vs. BNT162b2 2nd dose	gender = MALE	2.33	1.4 (0.78 to 2.49)	1.18 (0.65 to 2.14)	1
ChAdOx1 vs. BNT162b2 2nd dose	gender = FEMALE	2.68	1.58 (0.79 to 3.18)	1.17 (0.58 to 2.37)	1
**VTE**					
ChAdOx1 vs. BNT162b2 1st dose	age group: 30 - 39	3.81	1.04 (0.41 to 2.82)	1.38 (0.48 to 3.93)	1
ChAdOx1 vs. BNT162b2 1st dose	age group: 40 - 49	2.66	0.57 (0.3 to 1.1)	0.75 (0.39 to 1.45)	1
ChAdOx1 vs. BNT162b2 1st dose	age group: 50 - 59	1.93	0.56 (0.36 to 0.87)	0.68 (0.44 to 1.05)	1
ChAdOx1 vs. BNT162b2 1st dose	age group: 60 - 69	1.61	0.72 (0.53 to 1)	0.81 (0.53 to 1.23)	1
ChAdOx1 vs. BNT162b2 1st dose	age group: 70 - 79	1.42	0.8 (0.63 to 1.01)	1.01 (0.79 to 1.3)	1
ChAdOx1 vs. BNT162b2 1st dose	age group: 80 - 89	1.62	1.13 (0.81 to 1.59)	1.11 (0.77 to 1.59)	1
ChAdOx1 vs. BNT162b2 1st dose	gender = MALE	1.36	0.77 (0.63 to 0.95)	0.88 (0.7 to 1.11)	1
ChAdOx1 vs. BNT162b2 1st dose	gender = FEMALE	1.35	0.75 (0.61 to 0.93)	0.92 (0.74 to 1.14)	1
ChAdOx1 vs. BNT162b2 2nd dose	age group: 70 - 79	1.74	1.08 (0.73 to 1.59)	0.97 (0.64 to 1.47)	1
ChAdOx1 vs. BNT162b2 2nd dose	age group: 80 - 89	1.88	0.9 (0.56 to 1.41)	0.75 (0.44 to 1.3)	1
ChAdOx1 vs. BNT162b2 2nd dose	gender = MALE	1.66	1.41 (0.99 to 1.99)	1.19 (0.82 to 1.71)	1
ChAdOx1 vs. BNT162b2 2nd dose	gender = FEMALE	1.6	0.92 (0.65 to 1.29)	0.68 (0.47 to 1)	1

Supplementary Table 13. Age and sex stratified incidence rate ratio, US Open Claims.

Target vs. Comparator	Subgroup	MDRR	IRR	calibrated IRR	NCO eligibility
**US_Open_Claims**					
**ATE**					
Ad26.COV2.S vs. BNT162b2 1st dose	age group: 20 - 29	3.95	5.89 (2.78 to 12.82)	**4.64 (2.16 to 9.97)**	1
Ad26.COV2.S vs. BNT162b2 1st dose	age group: 30 - 39	2.7	1.77 (0.95 to 3.12)	1.21 (0.56 to 2.6)	1
Ad26.COV2.S vs. BNT162b2 1st dose	age group: 40 - 49	1.74	1.26 (0.87 to 1.79)	0.74 (0.26 to 2.09)	0
Ad26.COV2.S vs. BNT162b2 1st dose	age group: 50 - 59	1.35	1.51 (1.24 to 1.82)	1.07 (0.52 to 2.19)	0
Ad26.COV2.S vs. BNT162b2 1st dose	age group: 60 - 69	1.23	1.01 (0.87 to 1.17)	0.84 (0.5 to 1.43)	1
Ad26.COV2.S vs. BNT162b2 1st dose	age group: 70 - 79	1.28	0.98 (0.83 to 1.16)	0.64 (0.3 to 1.39)	0
Ad26.COV2.S vs. BNT162b2 1st dose	age group: 80 - 89	1.35	0.75 (0.59 to 0.94)	**0.58 (0.36 to 0.95)**	1
Ad26.COV2.S vs. BNT162b2 1st dose	gender = MALE	1.17	1.23 (1.11 to 1.37)	0.93 (0.58 to 1.5)	0
Ad26.COV2.S vs. BNT162b2 1st dose	gender = FEMALE	1.22	1.04 (0.9 to 1.19)	0.8 (0.48 to 1.34)	0
Ad26.COV2.S vs. mRNA-1273 1st dose	age group: 20 - 29	3.9	6.01 (2.8 to 13.43)	**5.1 (1.71 to 15.19)**	1
Ad26.COV2.S vs. mRNA-1273 1st dose	age group: 30 - 39	2.34	1.16 (0.63 to 2.01)	0.64 (0.34 to 1.2)	1
Ad26.COV2.S vs. mRNA-1273 1st dose	age group: 40 - 49	1.64	0.95 (0.66 to 1.34)	0.51 (0.18 to 1.4)	0
Ad26.COV2.S vs. mRNA-1273 1st dose	age group: 50 - 59	1.32	1.21 (1 to 1.45)	0.83 (0.41 to 1.67)	0
Ad26.COV2.S vs. mRNA-1273 1st dose	age group: 60 - 69	1.25	1.19 (1.02 to 1.38)	0.98 (0.81 to 1.19)	1
Ad26.COV2.S vs. mRNA-1273 1st dose	age group: 70 - 79	1.28	1.11 (0.93 to 1.31)	0.66 (0.3 to 1.46)	0
Ad26.COV2.S vs. mRNA-1273 1st dose	age group: 80 - 89	1.39	1.03 (0.81 to 1.29)	0.77 (0.27 to 2.21)	0
Ad26.COV2.S vs. mRNA-1273 1st dose	gender = MALE	1.18	1.42 (1.27 to 1.58)	0.96 (0.64 to 1.45)	0
Ad26.COV2.S vs. mRNA-1273 1st dose	gender = FEMALE	1.21	0.88 (0.77 to 1.01)	0.63 (0.35 to 1.12)	0
**DVT**					
Ad26.COV2.S vs. BNT162b2 1st dose	age group: 40 - 49	2.76	1.49 (0.76 to 2.75)	0.87 (0.27 to 2.85)	0
Ad26.COV2.S vs. BNT162b2 1st dose	age group: 50 - 59	2.02	0.99 (0.59 to 1.59)	0.7 (0.29 to 1.67)	0

Ad26.COV2.S vs. BNT162b2 1st dose	age group: 60 - 69	1.68	1.17 (0.81 to 1.64)	0.97 (0.52 to 1.82)	1
Ad26.COV2.S vs. BNT162b2 1st dose	age group: 70 - 79	2.03	1.27 (0.78 to 2.01)	0.83 (0.34 to 2.05)	0
Ad26.COV2.S vs. BNT162b2 1st dose	age group: 80 - 89	2.49	1.13 (0.58 to 2.06)	0.87 (0.4 to 1.91)	1
Ad26.COV2.S vs. BNT162b2 1st dose	gender = MALE	1.58	1.37 (1.02 to 1.83)	1.04 (0.6 to 1.8)	0
Ad26.COV2.S vs. BNT162b2 1st dose	gender = FEMALE	1.55	1.13 (0.84 to 1.51)	0.87 (0.49 to 1.56)	0
Ad26.COV2.S vs. mRNA-1273 1st dose	age group: 40 - 49	3.13	2.1 (1.02 to 4.17)	1.12 (0.34 to 3.7)	0
Ad26.COV2.S vs. mRNA-1273 1st dose	age group: 50 - 59	2.09	1.16 (0.68 to 1.87)	0.79 (0.34 to 1.87)	0
Ad26.COV2.S vs. mRNA-1273 1st dose	age group: 60 - 69	1.73	1.4 (0.97 to 1.98)	1.15 (0.78 to 1.69)	1
Ad26.COV2.S vs. mRNA-1273 1st dose	age group: 70 - 79	1.95	1.1 (0.68 to 1.71)	0.65 (0.26 to 1.62)	0
Ad26.COV2.S vs. mRNA-1273 1st dose	age group: 80 - 89	2.43	1.11 (0.58 to 2)	0.83 (0.25 to 2.8)	0
Ad26.COV2.S vs. mRNA-1273 1st dose	gender = MALE	1.54	1.22 (0.91 to 1.63)	0.83 (0.5 to 1.36)	0
Ad26.COV2.S vs. mRNA-1273 1st dose	gender = FEMALE	1.59	1.37 (1.01 to 1.84)	0.99 (0.52 to 1.87)	0
**Intestinal infarction**					
Ad26.COV2.S vs. BNT162b2 1st dose	gender = MALE	3.49	0.95 (0.35 to 2.17)	0.72 (0.24 to 2.16)	0
Ad26.COV2.S vs. mRNA-1273 1st dose	gender = MALE	3.18	0.68 (0.25 to 1.58)	0.46 (0.16 to 1.36)	0
**Ischemic stroke**					
Ad26.COV2.S vs. BNT162b2 1st dose	age group: 30 - 39	4.62	2.43 (0.97 to 5.56)	1.66 (0.6 to 4.58)	1
Ad26.COV2.S vs. BNT162b2 1st dose	age group: 40 - 49	2.55	2.22 (1.27 to 3.78)	1.3 (0.42 to 4)	0
Ad26.COV2.S vs. BNT162b2 1st dose	age group: 50 - 59	1.81	1.57 (1.07 to 2.27)	1.11 (0.5 to 2.46)	0
Ad26.COV2.S vs. BNT162b2 1st dose	age group: 60 - 69	1.57	1.09 (0.79 to 1.47)	0.91 (0.5 to 1.66)	1
Ad26.COV2.S vs. BNT162b2 1st dose	age group: 70 - 79	1.64	0.97 (0.67 to 1.35)	0.63 (0.27 to 1.46)	0
Ad26.COV2.S vs. BNT162b2 1st dose	age group: 80 - 89	1.87	1.14 (0.73 to 1.72)	0.88 (0.48 to 1.63)	1
Ad26.COV2.S vs. BNT162b2 1st dose	gender = MALE	1.42	1.38 (1.09 to 1.72)	1.04 (0.62 to 1.75)	0
Ad26.COV2.S vs. BNT162b2 1st dose	gender = FEMALE	1.43	1.2 (0.94 to 1.52)	0.93 (0.53 to 1.61)	0
Ad26.COV2.S vs. mRNA-1273 1st dose	age group: 30 - 39	4.55	2.45 (0.96 to 5.87)	1.34 (0.52 to 3.48)	1
Ad26.COV2.S vs. mRNA-1273 1st dose	age group: 40 - 49	2.44	1.95 (1.11 to 3.31)	1.04 (0.34 to 3.12)	0
Ad26.COV2.S vs. mRNA-1273 1st dose	age group: 50 - 59	1.83	1.62 (1.09 to 2.36)	1.11 (0.51 to 2.42)	0
Ad26.COV2.S vs. mRNA-1273 1st dose	age group: 60 - 69	1.6	1.23 (0.89 to 1.66)	1.01 (0.72 to 1.42)	1
Ad26.COV2.S vs. mRNA-1273 1st dose	age group: 70 - 79	1.63	0.96 (0.67 to 1.35)	0.57 (0.24 to 1.35)	0
Ad26.COV2.S vs. mRNA-1273 1st dose	age group: 80 - 89	1.84	1.09 (0.71 to 1.63)	0.82 (0.27 to 2.49)	0
Ad26.COV2.S vs. mRNA-1273 1st dose	gender = MALE	1.43	1.48 (1.17 to 1.85)	1 (0.63 to 1.58)	0
Ad26.COV2.S vs. mRNA-1273 1st dose	gender = FEMALE	1.42	1.15 (0.9 to 1.45)	0.82 (0.45 to 1.51)	0
**MI**					
Ad26.COV2.S vs. BNT162b2 1st dose	age group: 40 - 49	2.81	0.95 (0.43 to 1.89)	0.55 (0.16 to 1.96)	0
Ad26.COV2.S vs. BNT162b2 1st dose	age group: 50 - 59	1.84	1.21 (0.79 to 1.79)	0.85 (0.38 to 1.92)	0
Ad26.COV2.S vs. BNT162b2 1st dose	age group: 60 - 69	1.62	1.55 (1.14 to 2.09)	1.3 (0.72 to 2.36)	1
Ad26.COV2.S vs. BNT162b2 1st dose	age group: 70 - 79	1.68	0.86 (0.58 to 1.24)	0.56 (0.24 to 1.31)	0
Ad26.COV2.S vs. BNT162b2 1st dose	age group: 80 - 89	1.88	0.95 (0.6 to 1.47)	0.74 (0.39 to 1.39)	1
Ad26.COV2.S vs. BNT162b2 1st dose	gender = MALE	1.41	1.23 (0.98 to 1.54)	0.93 (0.55 to 1.57)	0
Ad26.COV2.S vs. BNT162b2 1st dose	gender = FEMALE	1.54	1.37 (1.03 to 1.79)	1.05 (0.6 to 1.86)	0
Ad26.COV2.S vs. mRNA-1273 1st dose	age group: 40 - 49	2.51	0.72 (0.33 to 1.42)	0.38 (0.11 to 1.32)	0
Ad26.COV2.S vs. mRNA-1273 1st dose	age group: 50 - 59	1.86	1.33 (0.87 to 1.98)	0.91 (0.41 to 2.03)	0
Ad26.COV2.S vs. mRNA-1273 1st dose	age group: 60 - 69	1.57	1.31 (0.96 to 1.75)	1.07 (0.77 to 1.49)	1

Ad26.COV2.S vs. mRNA-1273 1st dose	age group: 70 - 79	1.74	1.11 (0.75 to 1.6)	0.66 (0.28 to 1.58)	0
Ad26.COV2.S vs. mRNA-1273 1st dose	age group: 80 - 89	1.95	1.19 (0.75 to 1.82)	0.89 (0.29 to 2.74)	0
Ad26.COV2.S vs. mRNA-1273 1st dose	gender = MALE	1.41	1.27 (1.01 to 1.59)	0.86 (0.54 to 1.36)	0
Ad26.COV2.S vs. mRNA-1273 1st dose	gender = FEMALE	1.49	1.15 (0.87 to 1.5)	0.82 (0.44 to 1.54)	0
**PE**					
Ad26.COV2.S vs. BNT162b2 1st dose	age group: 30 - 39	3.69	2.06 (0.91 to 4.36)	1.41 (0.56 to 3.56)	1
Ad26.COV2.S vs. BNT162b2 1st dose	age group: 40 - 49	3.08	1.8 (0.88 to 3.46)	1.05 (0.31 to 3.53)	0
Ad26.COV2.S vs. BNT162b2 1st dose	age group: 50 - 59	2.21	1.7 (1.02 to 2.74)	1.2 (0.51 to 2.83)	0
Ad26.COV2.S vs. BNT162b2 1st dose	age group: 60 - 69	2.04	0.95 (0.56 to 1.54)	0.79 (0.38 to 1.66)	1
Ad26.COV2.S vs. BNT162b2 1st dose	age group: 70 - 79	2.08	1.74 (1.08 to 2.75)	1.14 (0.47 to 2.77)	0
Ad26.COV2.S vs. BNT162b2 1st dose	age group: 80 - 89	3.47	1.52 (0.65 to 3.3)	1.18 (0.46 to 3.06)	1
Ad26.COV2.S vs. BNT162b2 1st dose	gender = MALE	1.68	1.45 (1.04 to 2)	1.1 (0.62 to 1.95)	0
Ad26.COV2.S vs. BNT162b2 1st dose	gender = FEMALE	1.65	1.53 (1.1 to 2.08)	1.18 (0.65 to 2.13)	0
Ad26.COV2.S vs. mRNA-1273 1st dose	age group: 30 - 39	4.14	2.86 (1.2 to 6.7)	1.57 (0.65 to 3.81)	1
Ad26.COV2.S vs. mRNA-1273 1st dose	age group: 40 - 49	3.26	2 (0.93 to 4.19)	1.07 (0.31 to 3.62)	0
Ad26.COV2.S vs. mRNA-1273 1st dose	age group: 50 - 59	2.15	1.56 (0.94 to 2.5)	1.07 (0.46 to 2.48)	0
Ad26.COV2.S vs. mRNA-1273 1st dose	age group: 60 - 69	2.08	1.07 (0.63 to 1.74)	0.88 (0.51 to 1.52)	1
Ad26.COV2.S vs. mRNA-1273 1st dose	age group: 70 - 79	2.21	2.27 (1.36 to 3.76)	1.35 (0.53 to 3.42)	0
Ad26.COV2.S vs. mRNA-1273 1st dose	age group: 80 - 89	3.45	1.68 (0.75 to 3.36)	1.26 (0.34 to 4.63)	0
Ad26.COV2.S vs. mRNA-1273 1st dose	gender = MALE	1.69	1.52 (1.08 to 2.11)	1.03 (0.61 to 1.73)	0
Ad26.COV2.S vs. mRNA-1273 1st dose	gender = FEMALE	1.68	1.72 (1.24 to 2.36)	1.24 (0.65 to 2.36)	0
**Thrombocytopenia**					
Ad26.COV2.S vs. BNT162b2 1st dose	age group: 20 - 29	4.21	1.62 (0.61 to 3.82)	1.27 (0.48 to 3.39)	1
Ad26.COV2.S vs. BNT162b2 1st dose	age group: 30 - 39	3.31	1.98 (0.95 to 3.82)	1.35 (0.57 to 3.2)	1
Ad26.COV2.S vs. BNT162b2 1st dose	age group: 40 - 49	2.43	3.06 (1.78 to 5.28)	1.79 (0.59 to 5.45)	0
Ad26.COV2.S vs. BNT162b2 1st dose	age group: 50 - 59	1.73	1.55 (1.08 to 2.18)	1.09 (0.5 to 2.38)	0
Ad26.COV2.S vs. BNT162b2 1st dose	age group: 60 - 69	1.67	1.09 (0.76 to 1.54)	0.91 (0.49 to 1.71)	1
Ad26.COV2.S vs. BNT162b2 1st dose	age group: 70 - 79	1.89	0.79 (0.47 to 1.25)	0.52 (0.21 to 1.28)	0
Ad26.COV2.S vs. BNT162b2 1st dose	age group: 80 - 89	2.12	0.93 (0.52 to 1.55)	0.72 (0.35 to 1.46)	1
Ad26.COV2.S vs. BNT162b2 1st dose	gender = MALE	1.43	1.33 (1.05 to 1.68)	1.01 (0.6 to 1.7)	0
Ad26.COV2.S vs. BNT162b2 1st dose	gender = FEMALE	1.5	1.26 (0.96 to 1.64)	0.97 (0.55 to 1.71)	0
Ad26.COV2.S vs. mRNA-1273 1st dose	age group: 20 - 29	3.2	1.03 (0.41 to 2.23)	0.87 (0.26 to 2.91)	1
Ad26.COV2.S vs. mRNA-1273 1st dose	age group: 30 - 39	3.4	2.09 (0.97 to 4.28)	1.15 (0.52 to 2.52)	1
Ad26.COV2.S vs. mRNA-1273 1st dose	age group: 40 - 49	2.37	2.88 (1.7 to 4.86)	1.53 (0.52 to 4.54)	0
Ad26.COV2.S vs. mRNA-1273 1st dose	age group: 50 - 59	1.71	1.43 (1 to 2.01)	0.98 (0.46 to 2.1)	0
Ad26.COV2.S vs. mRNA-1273 1st dose	age group: 60 - 69	1.67	1.07 (0.74 to 1.5)	0.88 (0.6 to 1.29)	1
Ad26.COV2.S vs. mRNA-1273 1st dose	age group: 70 - 79	1.94	0.92 (0.56 to 1.46)	0.55 (0.22 to 1.39)	0
Ad26.COV2.S vs. mRNA-1273 1st dose	age group: 80 - 89	2.1	0.92 (0.52 to 1.53)	0.69 (0.21 to 2.21)	0
Ad26.COV2.S vs. mRNA-1273 1st dose	gender = MALE	1.43	1.37 (1.08 to 1.73)	0.93 (0.58 to 1.48)	0
Ad26.COV2.S vs. mRNA-1273 1st dose	gender = FEMALE	1.47	1.12 (0.85 to 1.44)	0.8 (0.43 to 1.49)	0
**TTS_any**					
Ad26.COV2.S vs. BNT162b2 1st dose	age group: 30 - 39	3.59	2.38 (1.13 to 4.69)	1.63 (0.68 to 3.89)	1
Ad26.COV2.S vs. BNT162b2 1st dose	age group: 40 - 49	2.59	2.49 (1.38 to 4.47)	1.46 (0.47 to 4.56)	0

Ad26.COV2.S vs. BNT162b2 1st dose	age group: 50 - 59	1.87	1.58 (1.06 to 2.32)	1.12 (0.5 to 2.49)	0
Ad26.COV2.S vs. BNT162b2 1st dose	age group: 60 - 69	1.75	1.16 (0.79 to 1.67)	0.97 (0.51 to 1.84)	1
Ad26.COV2.S vs. BNT162b2 1st dose	age group: 70 - 79	2.05	0.92 (0.53 to 1.5)	0.6 (0.24 to 1.52)	0
Ad26.COV2.S vs. BNT162b2 1st dose	age group: 80 - 89	2.31	1.23 (0.69 to 2.11)	0.96 (0.46 to 1.97)	1
Ad26.COV2.S vs. BNT162b2 1st dose	gender = MALE	1.48	1.52 (1.18 to 1.94)	1.15 (0.68 to 1.95)	0
Ad26.COV2.S vs. BNT162b2 1st dose	gender = FEMALE	1.58	1.21 (0.89 to 1.63)	0.94 (0.52 to 1.68)	0
Ad26.COV2.S vs. mRNA-1273 1st dose	age group: 20 - 29	4.04	1.05 (0.34 to 2.64)	0.89 (0.23 to 3.5)	1
Ad26.COV2.S vs. mRNA-1273 1st dose	age group: 30 - 39	3.56	2.38 (1.09 to 4.9)	1.3 (0.59 to 2.88)	1
Ad26.COV2.S vs. mRNA-1273 1st dose	age group: 40 - 49	2.59	2.61 (1.45 to 4.65)	1.39 (0.45 to 4.25)	0
Ad26.COV2.S vs. mRNA-1273 1st dose	age group: 50 - 59	1.86	1.57 (1.05 to 2.33)	1.08 (0.49 to 2.37)	0
Ad26.COV2.S vs. mRNA-1273 1st dose	age group: 60 - 69	1.77	1.22 (0.82 to 1.77)	1 (0.66 to 1.52)	1
Ad26.COV2.S vs. mRNA-1273 1st dose	age group: 70 - 79	2.02	0.93 (0.54 to 1.51)	0.55 (0.21 to 1.43)	0
Ad26.COV2.S vs. mRNA-1273 1st dose	age group: 80 - 89	2.32	1.29 (0.72 to 2.19)	0.97 (0.3 to 3.13)	0
Ad26.COV2.S vs. mRNA-1273 1st dose	gender = MALE	1.49	1.65 (1.28 to 2.11)	1.12 (0.7 to 1.79)	0
Ad26.COV2.S vs. mRNA-1273 1st dose	gender = FEMALE	1.55	1.08 (0.79 to 1.44)	0.77 (0.41 to 1.46)	0
Ad26.COV2.S vs. BNT162b2 1st dose	gender = MALE	6.27	3.01 (0.99 to 8.47)	2.27 (0.68 to 7.59)	0
**VTE**					
Ad26.COV2.S vs. BNT162b2 1st dose	age group: 30 - 39	3.18	2.1 (1.06 to 3.94)	1.44 (0.64 to 3.26)	1
Ad26.COV2.S vs. BNT162b2 1st dose	age group: 40 - 49	2.23	1.41 (0.82 to 2.31)	0.82 (0.27 to 2.5)	0
Ad26.COV2.S vs. BNT162b2 1st dose	age group: 50 - 59	1.77	1.23 (0.83 to 1.77)	0.87 (0.39 to 1.92)	0
Ad26.COV2.S vs. BNT162b2 1st dose	age group: 60 - 69	1.6	1.19 (0.86 to 1.62)	1 (0.55 to 1.82)	1
Ad26.COV2.S vs. BNT162b2 1st dose	age group: 70 - 79	1.73	1.47 (1.02 to 2.09)	0.96 (0.42 to 2.22)	0
Ad26.COV2.S vs. BNT162b2 1st dose	age group: 80 - 89	2.24	1.06 (0.59 to 1.81)	0.82 (0.4 to 1.69)	1
Ad26.COV2.S vs. BNT162b2 1st dose	gender = MALE	1.45	1.34 (1.05 to 1.7)	1.01 (0.6 to 1.72)	0
Ad26.COV2.S vs. BNT162b2 1st dose	gender = FEMALE	1.43	1.34 (1.06 to 1.68)	1.03 (0.6 to 1.79)	0
Ad26.COV2.S vs. mRNA-1273 1st dose	age group: 30 - 39	2.64	1.31 (0.67 to 2.42)	0.72 (0.36 to 1.44)	1
Ad26.COV2.S vs. mRNA-1273 1st dose	age group: 40 - 49	2.45	1.91 (1.08 to 3.31)	1.02 (0.34 to 3.09)	0
Ad26.COV2.S vs. mRNA-1273 1st dose	age group: 50 - 59	1.77	1.24 (0.84 to 1.79)	0.85 (0.39 to 1.85)	0
Ad26.COV2.S vs. mRNA-1273 1st dose	age group: 60 - 69	1.61	1.27 (0.92 to 1.72)	1.04 (0.74 to 1.47)	1
Ad26.COV2.S vs. mRNA-1273 1st dose	age group: 70 - 79	1.71	1.4 (0.98 to 1.98)	0.83 (0.35 to 1.96)	0
Ad26.COV2.S vs. mRNA-1273 1st dose	age group: 80 - 89	2.35	1.42 (0.8 to 2.37)	1.06 (0.33 to 3.42)	0
Ad26.COV2.S vs. mRNA-1273 1st dose	gender = MALE	1.44	1.26 (0.99 to 1.6)	0.85 (0.53 to 1.36)	0
Ad26.COV2.S vs. mRNA-1273 1st dose	gender = FEMALE	1.45	1.52 (1.2 to 1.92)	1.09 (0.59 to 2.01)	0

Supplementary Table 14. Cohort selection and minimal detectable relative risk, database-target-outcome.

	Original cohort		Without prior outcomes		With at least 1 days at risk		Matched on propensity score		MDRR
**Outcome**	**Comparator**	**Target**	**Comparator**	**Target**	**Comparator**	**Target**	**Comparator**	**Target**	
**CPRD Aurum**	**ChAdOx1 1st dose vs. BNT162b2 1st dose**								
CVST	1840240	3789631	1839907	3789045	1839631	3788249	1946223	1271557	>12.97
DVT	1840240	3789631	1805035	3742139	1804763	3741359	1912752	1247556	1.36
Intestinal infarction	1840240	3789631	1838922	3787830	1838646	3787035	1945248	1270917	2.6
Ischemic stroke	1840240	3789631	1829966	3777750	1829692	3776958	1936816	1264894	1.59
ATE	1840240	3789631	1774242	3716815	1773973	3716038	1886308	1227495	1.23
MI	1840240	3789631	1783628	3727853	1783357	3727073	1895358	1233874	1.3
PE	1840240	3789631	1815678	3757964	1815404	3757176	1922818	1254781	1.3
SVT	1840240	3789631	1839557	3788555	1839281	3787759	1945829	1271295	>3.72
Thrombocytopenia	1840240	3789631	1724387	3622661	1724118	3621898	1836112	1195498	1.17
VTE	1840240	3789631	1785134	3716545	1784864	3715771	1893469	1233788	1.24
TTS_ATE	1840240	3789631	1839739	3789168	1839463	3788372	1946184	1271530	>6.12
TTS_CVST	1840240	3789631	1840240	3789629	1839964	3788833	1946593	1271821	Inf
TTS_DVT	1840240	3789631	1839885	3789215	1839609	3788419	1946277	1271591	>12.97
TTS_isc stroke	1840240	3789631	1840174	3789562	1839898	3788766	1946536	1271777	>12.97
TTS_MI	1840240	3789631	1839818	3789250	1839542	3788454	1946252	1271585	>6.12
TTS_PE	1840240	3789631	1840046	3789394	1839770	3788598	1946400	1271669	>6.12
TTS_SVT	1840240	3789631	1840211	3789592	1839935	3788796	1946566	1271800	>12.97
TTS_VTE	1840240	3789631	1839724	3789016	1839448	3788220	1946111	1271464	>5.23
TTS_all stroke	1840240	3789631	1839911	3789284	1839635	3788489	1946277	1271609	>6.12
TTS_any	1840240	3789631	1828042	3773269	1827767	3772477	1934651	1263613	1.52
**CPRD Aurum**	**ChAdOx1 2nd dose vs. BNT162b2 2nd dose**								
CVST	1369238	1195626	1369029	1195414	1368495	1194604	1084284	801323	Inf
DVT	1369238	1195626	1342243	1170572	1341717	1169773	1063064	784878	1.57
Intestinal infarction	1369238	1195626	1368146	1194574	1367612	1193764	1083567	800793	>4.82
Ischemic stroke	1369238	1195626	1361028	1187920	1360495	1187113	1078360	796695	2.21
ATE	1369238	1195626	1317653	1149297	1317134	1148502	1044491	770339	1.43
MI	1369238	1195626	1325126	1156405	1324606	1155608	1050018	774713	1.54
PE	1369238	1195626	1350256	1177764	1349726	1176965	1069375	789797	1.6
SVT	1369238	1195626	1368710	1195077	1368176	1194267	1084010	801100	>6.00
Thrombocytopenia	1369238	1195626	1276719	1116141	1276204	1115370	1012563	747810	1.27
VTE	1369238	1195626	1326817	1156159	1326295	1155369	1050916	775486	1.4
TTS_ATE	1369238	1195626	1368803	1195308	1368269	1194498	1084154	801245	>6.00
TTS_CVST	1369238	1195626	1369238	1195625	1368704	1194815	1084454	801467	Inf
TTS_DVT	1369238	1195626	1368947	1195370	1368413	1194560	1084231	801305	>6.00

TTS_isc stroke	1369238	1195626	1369184	1195576	1368650	1194766	1084417	801435	>6.00
TTS_MI	1369238	1195626	1368870	1195365	1368336	1194555	1084202	801289	>6.00
TTS_PE	1369238	1195626	1369079	1195475	1368545	1194665	1084319	801375	>12.61
TTS_SVT	1369238	1195626	1369216	1195607	1368682	1194797	1084432	801452	Inf
TTS_VTE	1369238	1195626	1368811	1195241	1368277	1194431	1084118	801225	>5.52
TTS_all stroke	1369238	1195626	1368976	1195381	1368442	1194571	1084262	801318	>6.00
TTS_any	1369238	1195626	1359432	1187076	1358901	1186267	1076722	795629	1.88
**Germany_DA**	**ChAdOx1 1st dose vs. BNT162b2 1st dose**								
CVST	391063	98562	391019	98555	350965	89711	215180	86344	>7.10
DVT	391063	98562	384716	97278	344916	88463	211587	85163	2.6
Intestinal infarction	391063	98562	390901	98529	350850	89687	215106	86325	Inf
Ischemic stroke	391063	98562	384122	96966	344214	88152	210616	84835	3.37
ATE	391063	98562	375271	94660	335573	85890	204702	82643	2.18
MI	391063	98562	381689	96137	341848	87339	208975	84048	2.81
PE	391063	98562	385227	97653	345384	88838	212362	85493	3.37
SVT	391063	98562	390884	98542	350832	89698	215110	86330	>15.98
Thrombocytopenia	391063	98562	373590	94196	334413	85500	204508	82281	1.79
VTE	391063	98562	380017	96507	340391	87718	209244	84436	2.15
TTS_ATE	391063	98562	390936	98535	350881	89691	215092	86322	>15.98
TTS_CVST	391063	98562	391063	98562	351008	89718	215207	86351	Inf
TTS_DVT	391063	98562	390996	98550	350943	89706	215166	86340	Inf
TTS_isc stroke	391063	98562	391009	98548	350954	89704	215149	86337	>15.98
TTS_MI	391063	98562	390987	98549	350932	89705	215148	86337	Inf
TTS_PE	391063	98562	390944	98542	350891	89698	215135	86332	>15.98
TTS_SVT	391063	98562	391052	98558	350997	89714	215198	86347	Inf
TTS_VTE	391063	98562	390929	98541	350877	89697	215139	86332	>15.98
TTS_all stroke	391063	98562	390954	98543	350902	89699	215114	86330	>15.98
**Germany_DA**	**Ad26.COV2.S vs. BNT162b2 1st dose**								
CVST	391063	37723	391019	37721	350965	18762	68021	18706	>8.62
DVT	391063	37723	384716	37360	344916	18485	67016	18428	>8.62
Intestinal infarction	391063	37723	390901	37713	350850	18758	68007	18701	Inf
Ischemic stroke	391063	37723	384122	37359	344214	18433	66774	18379	>8.61
ATE	391063	37723	375271	36825	335573	17966	65173	17914	>4.98
MI	391063	37723	381689	37169	341848	18277	66336	18223	>7.15
PE	391063	37723	385227	37492	345384	18581	67323	18525	>8.62
SVT	391063	37723	390884	37718	350832	18759	68009	18703	Inf
Thrombocytopenia	391063	37723	373590	36591	334413	17988	65217	17933	3.8
VTE	391063	37723	380017	37166	340391	18332	66431	18274	6.17
TTS_ATE	391063	37723	390936	37716	350881	18757	68004	18701	Inf

TTS_CVST	391063	37723	391063	37723	351008	18763	68026	18707	Inf
TTS_DVT	391063	37723	390996	37718	350943	18758	68001	18702	Inf
TTS_isc stroke	391063	37723	391009	37722	350954	18762	68024	18706	Inf
TTS_MI	391063	37723	390987	37717	350932	18758	68006	18702	Inf
TTS_PE	391063	37723	390944	37718	350891	18759	68008	18703	>21.04
TTS_SVT	391063	37723	391052	37723	350997	18763	68026	18707	Inf
TTS_VTE	391063	37723	390929	37714	350877	18755	67989	18699	>21.03
**IPCI**	**ChAdOx1 1st dose vs. BNT162b2 1st dose**								
CVST	218423	71083	218423	71083	210487	71001	49659	22239	Inf
DVT	218423	71083	216639	70509	208713	70427	49198	22039	>6.80
Intestinal infarction	218423	71083	218423	71083	210487	71001	49659	22239	Inf
Ischemic stroke	218423	71083	216518	70513	208584	70432	49162	22044	>6.80
ATE	218423	71083	211448	68697	203533	68619	47893	21489	3.54
MI	218423	71083	213239	69237	205322	69158	48370	21678	>5.05
PE	218423	71083	216686	70505	208768	70424	49225	22043	>6.22
SVT	218423	71083	218423	71083	210487	71001	49659	22239	Inf
Thrombocytopenia	218423	71083	214847	70030	206973	69952	48710	21854	>4.55
VTE	218423	71083	215067	69992	207158	69911	48804	21863	4.35
TTS_ATE	218423	71083	218410	71080	210474	70998	49649	22236	Inf
TTS_CVST	218423	71083	218423	71083	210487	71001	49659	22239	Inf
TTS_DVT	218423	71083	218417	71082	210481	71000	49655	22238	Inf
TTS_isc stroke	218423	71083	218421	71083	210485	71001	49659	22239	Inf
TTS_MI	218423	71083	218412	71080	210476	70998	49649	22236	Inf
TTS_PE	218423	71083	218410	71081	210474	70999	49653	22237	Inf
TTS_SVT	218423	71083	218423	71083	210487	71001	49659	22239	Inf
TTS_VTE	218423	71083	218407	71080	210471	70998	49650	22236	Inf
TTS_all stroke	218423	71083	218419	71082	210483	71000	49659	22239	Inf
**SIDIAP**	**Ad26.COV2.S vs. BNT162b2 1st dose**								
CVST	2027950	138351	2027844	138339	1990871	126938	423815	116789	Inf
DVT	2027950	138351	2009886	137540	1973028	126189	421532	116087	2.82
Intestinal infarction	2027950	138351	2025253	138252	1988290	126855	423558	116709	>8.61
Ischemic stroke	2027950	138351	1972241	136360	1935389	125015	417793	114999	2.15
ATE	2027950	138351	1940805	134695	1904070	123408	413039	113588	1.73
MI	2027950	138351	1993255	136561	1956402	125219	418734	115276	2.67
PE	2027950	138351	2015190	137815	1978276	126442	422330	116315	4.77
SVT	2027950	138351	2026906	138287	1989938	126887	423664	116747	>8.61
Thrombocytopenia	2027950	138351	1789028	126127	1754222	115521	386334	106217	1.54
VTE	2027950	138351	2001029	137188	1964216	125850	420502	115760	2.48
TTS_ATE	2027950	138351	2023991	138240	1987022	126844	423524	116696	>8.61

TTS_CVST	2027950	138351	2027942	138350	1990968	126949	423858	116802	Inf
TTS_DVT	2027950	138351	2027155	138321	1990183	126923	423781	116779	>8.61
TTS_isc stroke	2027950	138351	2025770	138290	1988798	126893	423667	116737	>21.00
TTS_MI	2027950	138351	2026290	138302	1989318	126902	423714	116762	>21.00
TTS_PE	2027950	138351	2027112	138316	1990144	126917	423767	116773	>21.00
TTS_SVT	2027950	138351	2027749	138343	1990776	126942	423830	116795	Inf
TTS_VTE	2027950	138351	2026620	138297	1989653	126901	423710	116760	>8.61
TTS_all stroke	2027950	138351	2025382	138280	1988414	126884	423637	116726	>21.00
**US_Open_Claims**	**Ad26.COV2.S**	**vs. BNT162b2 1st dose**							
CVST	6055754	939748	6055085	939659	5377556	639457	2405656	639456	>7.96
DVT	6055754	939748	5963034	927070	5288749	628004	2363428	628002	1.37
Intestinal infarction	6055754	939748	6046033	938328	5368789	638258	2401293	638257	2.43
Ischemic stroke	6055754	939748	5926489	922291	5253145	623397	2348140	623396	1.29
ATE	6055754	939748	5843606	908728	5172933	610896	2304844	610895	1.14
MI	6055754	939748	5956952	924232	5282441	625169	2356142	625168	1.31
PE	6055754	939748	6000736	932417	5325183	632835	2380869	632834	1.44
SVT	6055754	939748	6052148	939279	5374738	639112	2404366	639111	3.59
Thrombocytopenia	6055754	939748	5958632	926904	5285775	628294	2364195	628293	1.31
VTE	6055754	939748	5930163	922661	5257227	624003	2348419	624001	1.3
TTS_ATE	6055754	939748	6052570	939364	5375116	639180	2404622	639179	>4.49
TTS_CVST	6055754	939748	6055739	939747	5378182	639538	2405961	639537	>21.67
TTS_DVT	6055754	939748	6053805	939521	5376301	639334	2405147	639333	5.58
TTS_isc stroke	6055754	939748	6054244	939571	5376736	639374	2405354	639373	>6.74
TTS_MI	6055754	939748	6054026	939529	5376525	639336	2405217	639335	>6.74
TTS_PE	6055754	939748	6054201	939563	5376688	639368	2405319	639367	6.29
TTS_SVT	6055754	939748	6055459	939714	5377908	639507	2405815	639506	>8.80
TTS_VTE	6055754	939748	6053209	939451	5375727	639270	2404904	639269	4.07
TTS_all stroke	6055754	939748	6053844	939527	5376349	639334	2405207	639333	7.28
TTS_any	6055754	939748	5961410	927210	5288474	628572	2365254	628571	1.35
**US_Open_Claims**	**Ad26.COV2.S**	**vs. mRNA-1273 1st dose**							
CVST	4261016	939967	4260530	939878	4002663	639619	2272268	639619	>8.50
DVT	4261016	939967	4179884	927287	3923371	628164	2230157	628164	1.37
Intestinal infarction	4261016	939967	4251889	938545	3994166	638418	2267972	638418	2.38
Ischemic stroke	4261016	939967	4145567	922509	3889750	623557	2214613	623557	1.29
ATE	4261016	939967	4064922	908944	3810684	611054	2171445	611054	1.13
MI	4261016	939967	4166608	924449	3910513	625329	2222711	625329	1.3
PE	4261016	939967	4213302	932636	3956266	632997	2247746	632997	1.45
SVT	4261016	939967	4258228	939498	4000424	639274	2271071	639274	3.68
Thrombocytopenia	4261016	939967	4180086	927125	3924097	628459	2231498	628459	1.3

VTE	4261016	939967	4151615	922878	3895669	624163	2215499	624163	1.3
TTS_ATE	4261016	939967	4258299	939583	4000482	639342	2271303	639342	>3.51
TTS_CVST	4261016	939967	4261011	939966	4003140	639700	2272570	639700	>20.62
TTS_DVT	4261016	939967	4259478	939740	4001635	639496	2271774	639496	4.93
TTS_isc stroke	4261016	939967	4259779	939790	4001931	639536	2272003	639536	>5.43
TTS_MI	4261016	939967	4259484	939748	4001644	639498	2271842	639498	>4.72
TTS_PE	4261016	939967	4259644	939782	4001796	639530	2271912	639530	6.53
TTS_SVT	4261016	939967	4260797	939933	4002932	639669	2272454	639669	>8.50
TTS_VTE	4261016	939967	4258961	939670	4001130	639432	2271552	639432	3.87
TTS_all stroke	4261016	939967	4259463	939746	4001622	639496	2271845	639496	6.1
TTS_any	4261016	939967	4182368	927431	3926355	628737	2232550	628737	1.34

Supplementary Table 15. List of covariates with top 10 highest absolute value of propensity score model coefficient.

**Covariate**	**Coefficient**		**Covariate**	**Coefficient**
**CPRD_Aurum - ChAdOx1 1st dose - BNT162b2 1st dose**			**CPRD_Aurum - ChAdOx1 2nd dose - BNT162b2 2nd dose**	
index month: 12	-6.75		observation distinct concept count during day -180 through -4 concept_count relative to index	-7.05
observation distinct concept count during day -180 through -4 concept_count relative to index	-4.73		index month: 1	-6.61
visit_occurrence concept count during day -180 through -4 concept_count relative to index: Office Visit	4.51		index month: 2	-2.21
index month: 1	-4.46		observation during day -180 through -4 days relative to index: Housebound	1.95
measurement distinct concept count during day -180 through -4 concept_count relative to index	-4.08		procedure_occurrence during day -180 through -4 days relative to index: Administration of vaccine	-1.79
visit_occurrence concept count during day -180 through -4 concept_count relative to index	-4.07		index month: 12	-1.77
index year: 2020	-3.83		observation during day -180 through -4 days relative to index: Temporarily housebound	1.61
index month: 2	-3.33		measurement during day -180 through -4 days relative to index: Well person screening	1.57
procedure_occurrence during day -180 through -4 days relative to index: Administration of vaccine	-2.47		observation during day -180 through -4 days relative to index: Informed consent given for treatment	-1.56
drug_era distinct concept count during day -180 through -4 concept_count relative to index	-2.29		observation during day -180 through -4 days relative to index: Appointment cancelled by service	-1.44
**France_LPD - ChAdOx1 1st dose - BNT162b2 1st dose**			**France_LPD - ChAdOx1 1st dose - mRNA-1273 1st dose**	
index month: 1	-6.42		index month: 8	-6.64
CHADS2VASc	-6.00		index month: 9	-5.75
index month: 8	-4.69		index month: 1	-5.22
visit_occurrence concept count during day -180 through -4 concept_count relative to index	4.00		index month: 7	-4.10

age group: 20 - 29	-3.36		index month: 6	-3.69
index month: 9	-3.31		CHADS2VASc	-3.43
age group: 10 - 19	-3.08		age group: 20 - 29	-3.33
age group: 30 - 39	-3.04		age group: 10 - 19	-2.96
measurement during day -180 through -4 days relative to index: Potassium [Moles/time] in 24 hour Urine	-2.83		age group: 30 - 39	-2.92
age group: 40 - 49	-2.78		age group: 40 - 49	-2.88
Germany_DA - ChAdOx1 1st dose - BNT162b2 1st dose			Germany_DA - Janssen COVID-19- BNT162b2 1st dose	
index month: 8	-3.76		index month: 6	5.92
index month: 7	-2.64		index month: 8	4.96
index month: 3	2.28		measurement distinct concept count during day -180 through -4 concept_count relative to index	4.77
age group: 10 - 19	-1.77		index month: 5	4.66
index month: 9	-1.68		index month: 7	4.52
gender = MALE	1.44		index month: 9	4.06
index month: 6	-1.42		Charlson index - Romano adaptation	2.25
drug_era distinct concept count during day -180 through -4 concept_count relative to index	-1.24		drug_era distinct concept count during day -180 through -4 concept_count relative to index	2.02
index month: 4	-1.22		gender = FEMALE	-1.75
age group: 20 - 29	-1.05		age group: 10 - 19	-1.28
**Germany_DA - ChAdOx1 2nd dose - BNT162b2 2nd dose**			**SIDIAP - Ad26.COV2.S- BNT162b2 1st dose**	
index month: 4	-4.10		CHADS2VASc	-10.04
index month: 5	-2.20		index month: 1	-6.91
age group: 10 - 19	-2.15		index month: 3	-6.24
drug_era distinct concept count during day -180 through -4 concept_count relative to index	-1.67		index month: 2	-6.00
age group: 30 - 39	-1.44		index month: 12	-4.02
drug_era group during day -180 through -4 days relative to index: ascorbic acid	1.39		visit_occurrence concept count during day -180 through -4 concept_count relative to index: Telehealth	-3.95
age group: 20 - 29	-1.37		age group: 60 - 69	3.75
condition_era group during day -9999 through -4 days relative to index: Attention deficit hyperactivity disorder	-1.25		age group: 70 - 79	2.75
age group: 40 - 49	-1.24		visit_occurrence concept count during day -180 through -4 concept_count relative to index: Outpatient Visit	2.56
visit_occurrence concept count during day -180 through -4 concept_count relative to index: Office Visit	1.14		condition_era group during day -9999 through -4 days relative to index: Transient cerebral ischemia	1.95
**US_Open_Claims - Ad26.COV2.S - BNT162b2 1st dose**			**US_Open_Claims - Ad26.COV2.S - mRNA-1273 1st dose**	
index month: 1	-9.36		index month: 1	-8.60
index month: 2	-7.67		index month: 2	-7.62
visit_occurrence concept count during day -180 through -4 concept_count relative to index	5.51		visit_occurrence concept count during day -180 through -4 concept_count relative to index	3.91

visit_occurrence concept count during day -180 through -4 concept_count relative to index: Off Campus-Outpatient Hospital	-5.05		index month: 12	-3.84
index month: 12	-4.54		drug_era distinct concept count during day -180 through -4 concept_count relative to index	3.80
drug_era distinct concept count during day -180 through -4 concept_count relative to index	3.79		index year: 2020	-2.50
visit_occurrence concept count during day -180 through -4 concept_count relative to index: Rural Health Clinic	3.05		index month: 6	1.51
index year: 2020	-2.91		Charlson index - Romano adaptation	1.28
index month: 9	-1.95		observation distinct concept count during day -180 through -4 concept_count relative to index	1.21
Charlson index - Romano adaptation	1.69		index month: 9	0.94

Figures

Figure 1. Passed systematic error diagnostic: <20% negative control outcomes associated with exposure in the uncalibrated analyses (CPRD Aurum, 1st dose ChAdOx1 vs BNT162b2)

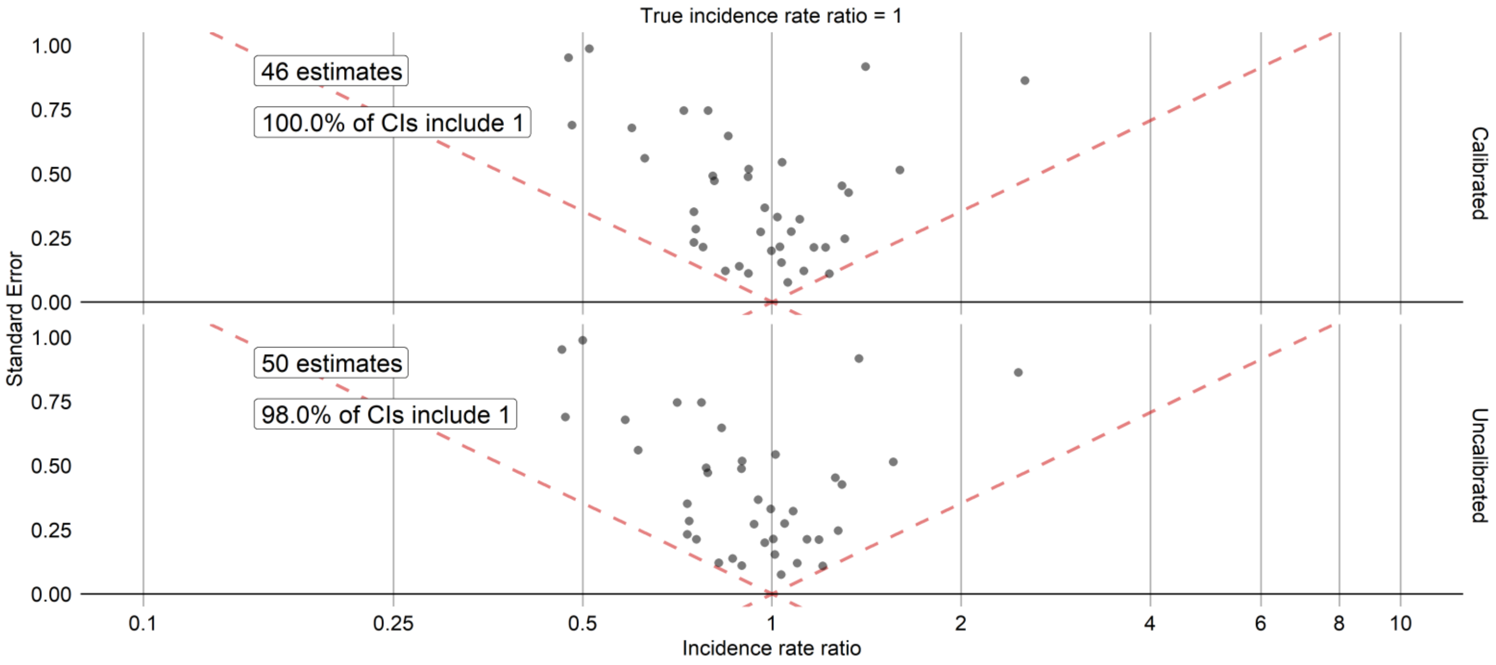

Estimates below the diagonal dashed lines are statistically significant (alpha = 0.05) different from the true effect size (expected IRR=1). A well-calibrated estimator should have the true effect size within the 95 percent confidence interval 95 percent of times.

Figure 2. Systematic error in the comparison between Janssen and mRNA vaccines in US Open Claims

Figure 2a. Systematic error in the analysis of Janssen vs 1-dose BNT162b2

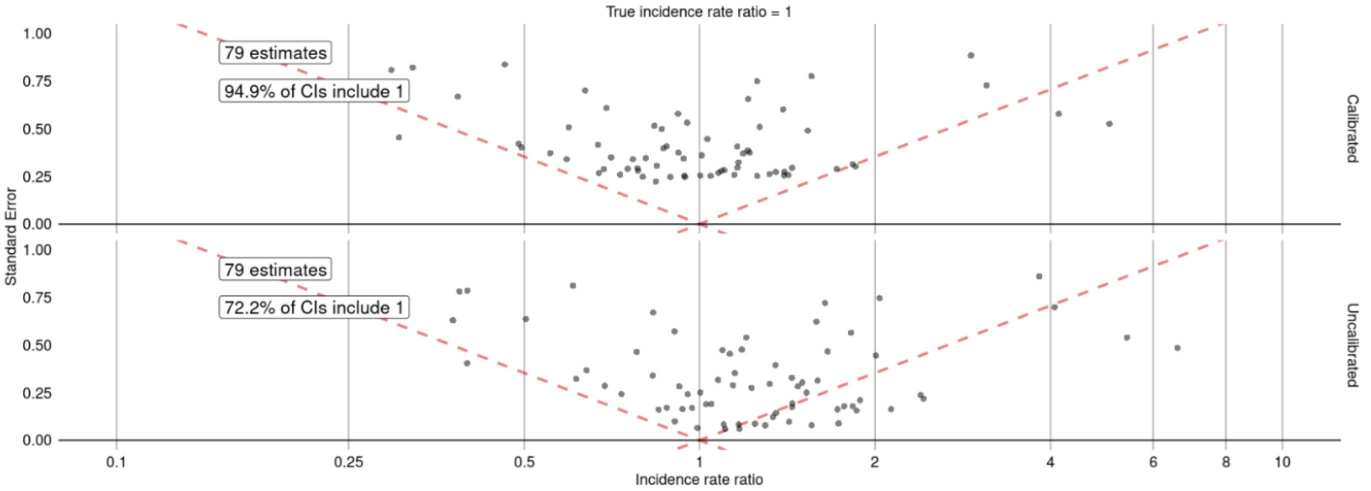

Figure 2b. Systematic error in the analysis of Janssen vs 1-dose mRNA-1273

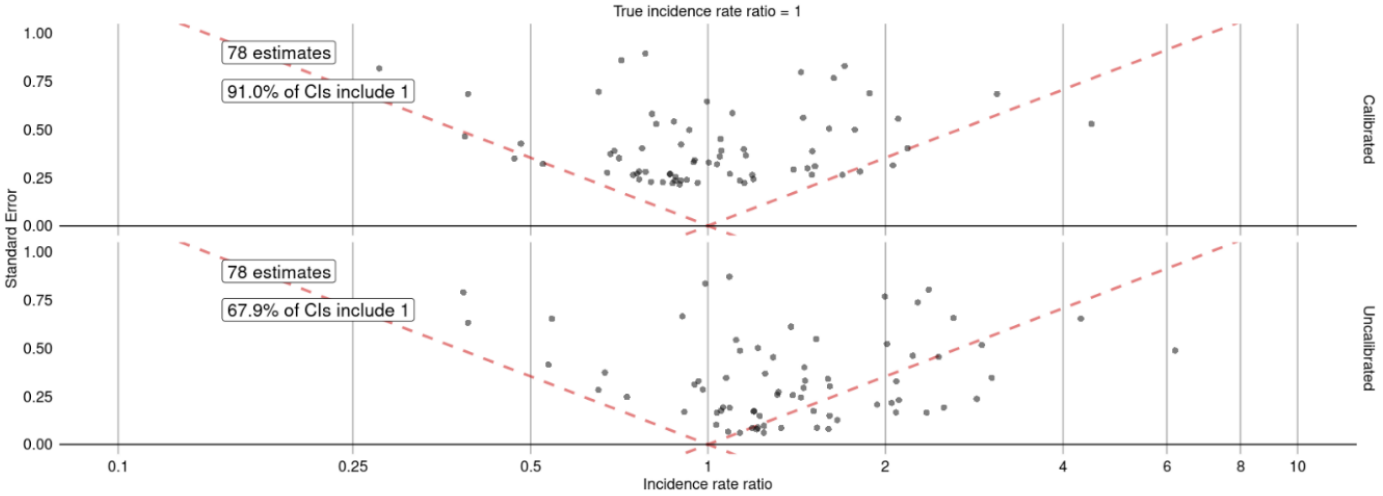

Figure 3. Systematic error in meta-analysis

Figure 3a. Systematic error in the analysis of first-dose ChAdOx1 vs 1-dose BNT162b2.

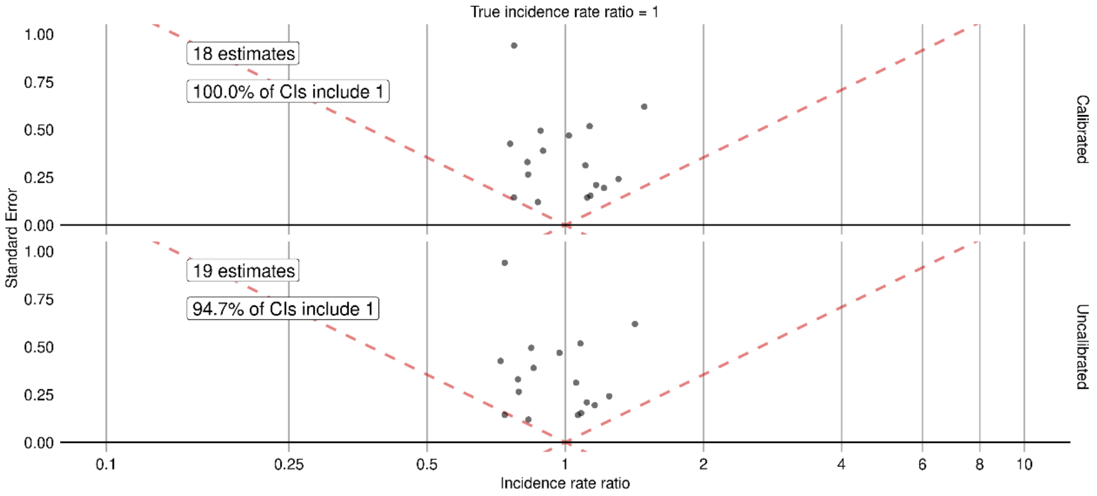

Figure 3b. Systematic error in the analysis of second-dose ChAdOx1 vs 1-dose BNT162b2.

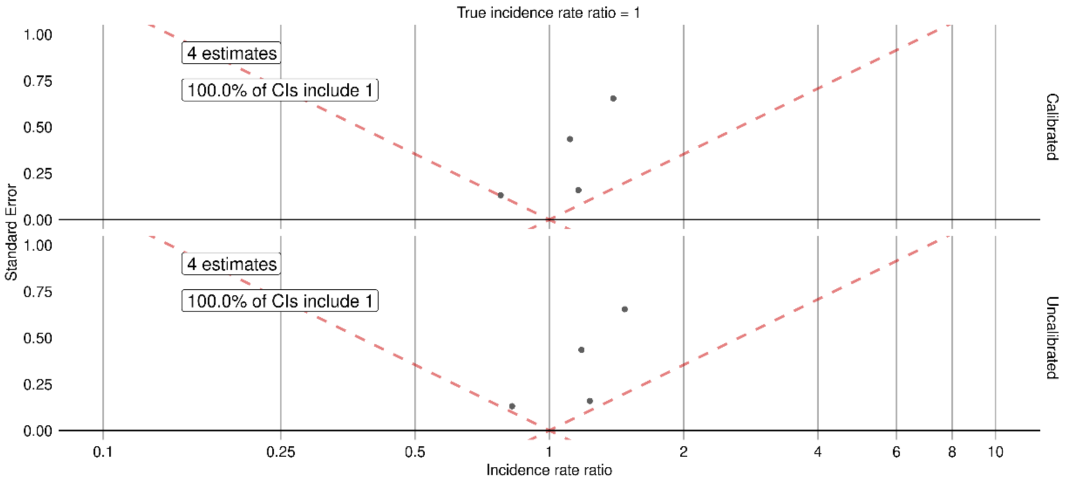

Figure 3c. Systematic error in the analysis of Ad26.COV2.S vs 1-dose BNT162

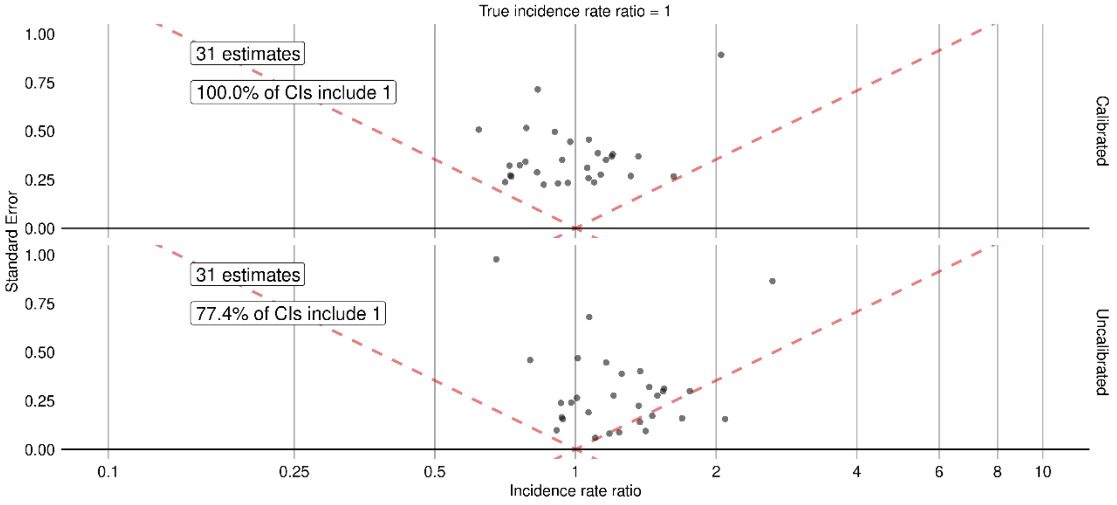

Supplement: Supplementary file 1 — Web appendix 1: Supplementary materials A [file lix071594.ww1.pdf]
