# Supplementary material for: Comparative risk of thrombosis with thrombocytopenia syndrome or thromboembolic events associated with different covid-19 vaccines: international network cohort study from five European countries and the US
Source: BMJ. 2022 Oct 26;379:e071594. doi: 10.1136/bmj-2022-071594 (PMC9597610; doi:10.1136/bmj-2022-071594)
Supplement: Supplementary file 2 — Web appendix 2: Supplementary materials B [file lix071594.ww2.pdf]

Supplementary Materials B

Concept set for persons vaccinated against SARS-CoV-2

Concept ID	Name	Vocabulary
59267100003	SARS-CoV-2 (COVID-19) vaccine, mRNA-BNT162b2 0.1 MG/ML Injectable Suspension	NDC
59267100002	SARS-CoV-2 (COVID-19) vaccine, mRNA-BNT162b2 0.1 MG/ML Injectable Suspension	NDC
592671000	bnt162b2 .23mg/1.8mL INTRAMUSCULAR INJECTION, SUSPENSION	NDC
80777027310	SARS-CoV-2 (COVID-19) vaccine, mRNA-1273 0.2 MG/ML Injectable Suspension	NDC
2470234	SARS-CoV-2 (COVID-19) vaccine, mRNA-1273 0.2 MG/ML Injectable Suspension	RxNorm
2470233	SARS-CoV-2 (COVID-19) vaccine, mRNA-1273 0.2 MG/ML	RxNorm
2470232	SARS-CoV-2 (COVID-19) vaccine, mRNA-1273	RxNorm
2468235	SARS-CoV-2 (COVID-19) vaccine, mRNA-BNT162b2 0.1 MG/ML Injectable Suspension	RxNorm
2468234	SARS-CoV-2 (COVID-19) vaccine, mRNA spike protein Injectable Suspension	RxNorm
2468233	SARS-CoV-2 (COVID-19) vaccine, mRNA spike protein Injectable Product	RxNorm
2468232	SARS-CoV-2 (COVID-19) vaccine, mRNA-BNT162b2 0.1 MG/ML	RxNorm
2468231	SARS-CoV-2 (COVID-19) vaccine, mRNA spike protein	RxNorm
2468230	SARS-CoV-2 (COVID-19) vaccine, mRNA-BNT162b2	RxNorm
80777027399	SARS-CoV-2 (COVID-19) vaccine, mRNA-1273 0.2 MG/ML Injectable Suspension	NDC
807770273	cx-024414 .2mg/mL INTRAMUSCULAR INJECTION, SUSPENSION	NDC
39214411000001100	Generic COVID-19 mRNA Vaccine BNT162b2 30micrograms/0.3ml dose concentrate for suspension for injection multidose vials (Pfizer-BioNTech) 1170 dose	dm+d
39326611000001100	Generic COVID-19 mRNA (nucleoside modified) Vaccine Moderna 0.1mg/0.5mL dose dispersion for injection multidose vials 100 dose	dm+d
39326811000001100	Generic COVID-19 mRNA (nucleoside modified) Vaccine Moderna 0.1mg/0.5mL dose dispersion for injection multidose vials	dm+d
39214511000001100	COVID-19 mRNA Vaccine BNT162b2 30micrograms/0.3ml dose concentrate for suspension for injection multidose vials (Pfizer-BioNTech) (Pfizer-BioNTech) 1170 dose 195 x 6 dose vials	dm+d
39327011000001100	COVID-19 mRNA (nucleoside modified) Vaccine Moderna 0.1mg/0.5mL dose dispersion for injection multidose vials (Moderna, Inc) 100 dose 10 x 10 dose vials	dm+d
39326911000001100	COVID-19 mRNA (nucleoside modified) Vaccine Moderna 0.1mg/0.5mL dose dispersion for injection multidose vials	dm+d

39115611000001100	COVID-19 mRNA Vaccine BNT162b2 30micrograms/0.3ml dose concentrate for suspension for injection multidose vials (Pfizer-BioNTech)	dm+d
39115311000001100	Generic COVID-19 mRNA Vaccine BNT162b2 30micrograms/0.3ml dose concentrate for suspension for injection multidose vials (Pfizer-BioNTech) 6 dose	dm+d
39115711000001100	COVID-19 mRNA Vaccine BNT162b2 30micrograms/0.3ml dose concentrate for suspension for injection multidose vials (Pfizer-BioNTech) (Pfizer-BioNTech) 6 dose	dm+d
39116111000001100	Generic COVID-19 mRNA Vaccine BNT162b2 30micrograms/0.3ml dose concentrate for suspension for injection multidose vials (Pfizer-BioNTech)	dm+d
59676058005	Janssen COVID-19 vaccine, DNA, spike protein, adenovirus type 26 (Ad26) vector, preservative free, 5x1010 viral particles/0.5mL dosage, for intramuscular use	NDC
310122210	AZD1222 Astrazeneca COVID-19 vaccine, DNA, spike protein, chimpanzee adenovirus Oxford 1 (ChAdOx1) vector, preservative free, 5x1010 viral particles/0.5mL dosage, for intramuscular use	NDC
310122215	azd1222 50000000000[VP]/.5mL INTRAMUSCULAR INJECTION, SUSPENSION	NDC
59267100001	SARS-CoV-2 (COVID-19) vaccine, mRNA-BNT162b2 0.1 MG/ML Injectable Suspension	NDC
91303	Janssen Covid-19 Vaccine	CPT4
91302	AstraZeneca Covid-19 Vaccine	CPT4
91301	Moderna Covid-19 Vaccine	CPT4
91300	Pfizer-Biontech Covid-19 Vaccine	CPT4
0022A	AstraZeneca Covid-19 Vaccine Administration - Second Dose	CPT4
0021A	AstraZeneca Covid-19 Vaccine Administration - First Dose	CPT4
0031A	Janssen Covid-19 Vaccine Administration	CPT4
0012A	Moderna Covid-19 Vaccine Administration - Second Dose	CPT4
0011A	Moderna Covid-19 Vaccine Administration - First Dose	CPT4
0002A	Pfizer-Biontech Covid-19 Vaccine Administration - Second Dose	CPT4
0001A	Pfizer-Biontech Covid-19 Vaccine Administration - First Dose	CPT4
208	SARS-COV-2 (COVID-19) vaccine, mRNA, spike protein, LNP, preservative free, 30 mcg/0.3mL dose	CVX
207	SARS-COV-2 (COVID-19) vaccine, mRNA, spike protein, LNP, preservative free, 100 mcg/0.5mL dose	CVX
210	SARS-COV-2 (COVID-19) vaccine, vector non-replicating, recombinant spike protein-ChAdOx1, preservative free, 0.5 mL	CVX
212	SARS-COV-2 (COVID-19) vaccine, vector non-replicating, recombinant spike protein-Ad26, preservative free, 0.5 mL	CVX

List of included concepts for study outcomes

Cerebral venous sinus thrombosis (CVST)

Concept ID	Concept name	Vocabulary	Is excluded?	Include descendants?
4102202	Cerebral venous sinus thrombosis	SNOMED	FALSE	FALSE
4048786	Cerebral venous thrombosis of sigmoid sinus	SNOMED	FALSE	FALSE
4043735	Cerebral venous thrombosis of straight sinus	SNOMED	FALSE	FALSE
4111713	Non-pyogenic venous sinus thrombosis	SNOMED	FALSE	FALSE
314667	Nonpyogenic thrombosis of intracranial venous sinus	SNOMED	FALSE	FALSE
4116206	Septic thrombophlebitis of cavernous sinus	SNOMED	FALSE	FALSE
4121335	Septic thrombophlebitis of lateral sinus	SNOMED	FALSE	FALSE
4119136	Septic thrombophlebitis of sagittal sinus	SNOMED	FALSE	FALSE
4041680	Septic thrombophlebitis of sigmoid sinus	SNOMED	FALSE	FALSE
4100225	Thrombophlebitis lateral venous sinus	SNOMED	FALSE	FALSE
4217471	Thrombophlebitis of basilar sinus	SNOMED	FALSE	FALSE
4104695	Thrombophlebitis of cavernous sinus	SNOMED	FALSE	FALSE
4167985	Thrombophlebitis of inferior sagittal sinus	SNOMED	FALSE	FALSE
764714	Thrombophlebitis of sigmoid sinus	SNOMED	FALSE	FALSE
4100224	Thrombophlebitis of superior longitudinal venous sinus	SNOMED	FALSE	FALSE
4098706	Thrombophlebitis of superior sagittal sinus	SNOMED	FALSE	FALSE
4277833	Thrombophlebitis of torcular Herophili	SNOMED	FALSE	FALSE
764710	Thrombophlebitis of transverse sinus	SNOMED	FALSE	FALSE
4228209	Thrombosis of basilar sinus	SNOMED	FALSE	FALSE
4234264	Thrombosis of cavernous venous sinus	SNOMED	FALSE	FALSE
4048890	Thrombosis of inferior sagittal sinus	SNOMED	FALSE	FALSE
4057329	Thrombosis of lateral venous sinus	SNOMED	FALSE	FALSE
4102203	Thrombosis of superior longitudinal sinus	SNOMED	FALSE	FALSE
4290940	Thrombosis of superior sagittal sinus	SNOMED	FALSE	FALSE
4079905	Thrombosis of torcular Herophili	SNOMED	FALSE	FALSE
4105338	Thrombosis transverse sinus	SNOMED	FALSE	FALSE

Deep vein thrombosis

Concept ID	Concept name	Vocabulary	Is excluded?	Include descendants?
762047	Acute bilateral thrombosis of subclavian veins	SNOMED	FALSE	FALSE
762148	Acute deep vein thrombosis of bilateral iliac veins	SNOMED	FALSE	FALSE
761444	Acute deep vein thrombosis of bilateral lower limbs following coronary artery bypass graft	SNOMED	FALSE	FALSE
35616028	Acute deep vein thrombosis of left iliac vein	SNOMED	FALSE	FALSE
35615035	Acute deep vein thrombosis of left lower limb following procedure	SNOMED	FALSE	FALSE

761416	Acute deep vein thrombosis of left upper limb following coronary artery bypass graft	SNOMED	FALSE	FALSE
35615031	Acute deep vein thrombosis of left upper limb following procedure	SNOMED	FALSE	FALSE
43531681	Acute deep vein thrombosis of lower limb	SNOMED	FALSE	FALSE
35616027	Acute deep vein thrombosis of right iliac vein	SNOMED	FALSE	FALSE
35615034	Acute deep vein thrombosis of right lower limb following procedure	SNOMED	FALSE	FALSE
761415	Acute deep vein thrombosis of right upper limb following coronary artery bypass graft	SNOMED	FALSE	FALSE
35615030	Acute deep vein thrombosis of right upper limb following procedure	SNOMED	FALSE	FALSE
44782746	Acute deep venous thrombosis	SNOMED	FALSE	FALSE
44782751	Acute deep venous thrombosis of axillary vein	SNOMED	FALSE	FALSE
762008	Acute deep venous thrombosis of bilateral axillary veins	SNOMED	FALSE	FALSE
760875	Acute deep venous thrombosis of bilateral calves	SNOMED	FALSE	FALSE
765155	Acute deep venous thrombosis of bilateral ileofemoral veins	SNOMED	FALSE	FALSE
762017	Acute deep venous thrombosis of bilateral internal jugular veins	SNOMED	FALSE	FALSE
762417	Acute deep venous thrombosis of bilateral legs	SNOMED	FALSE	FALSE
762020	Acute deep venous thrombosis of bilateral popliteal veins	SNOMED	FALSE	FALSE
765546	Acute deep venous thrombosis of bilateral tibial veins	SNOMED	FALSE	FALSE
762004	Acute deep venous thrombosis of both upper extremities	SNOMED	FALSE	FALSE
44782742	Acute deep venous thrombosis of calf	SNOMED	FALSE	FALSE
44782747	Acute deep venous thrombosis of femoral vein	SNOMED	FALSE	FALSE
762015	Acute deep venous thrombosis of ileofemoral vein of left leg	SNOMED	FALSE	FALSE
765541	Acute deep venous thrombosis of ileofemoral vein of right lower extremity	SNOMED	FALSE	FALSE
44782748	Acute deep venous thrombosis of iliofemoral vein	SNOMED	FALSE	FALSE
44782752	Acute deep venous thrombosis of internal jugular vein	SNOMED	FALSE	FALSE
762009	Acute deep venous thrombosis of left axillary vein	SNOMED	FALSE	FALSE
760876	Acute deep venous thrombosis of left calf	SNOMED	FALSE	FALSE
765540	Acute deep venous thrombosis of left femoral vein	SNOMED	FALSE	FALSE
765922	Acute deep venous thrombosis of left internal jugular vein	SNOMED	FALSE	FALSE
762418	Acute deep venous thrombosis of left lower extremity	SNOMED	FALSE	FALSE

765537	Acute deep venous thrombosis of left upper extremity	SNOMED	FALSE	FALSE
44782767	Acute deep venous thrombosis of lower extremity as complication of procedure	SNOMED	FALSE	FALSE
46270071	Acute deep venous thrombosis of lower limb due to coronary artery bypass grafting	SNOMED	FALSE	FALSE
762022	Acute deep venous thrombosis of politeal vein of right leg	SNOMED	FALSE	FALSE
44782743	Acute deep venous thrombosis of popliteal vein	SNOMED	FALSE	FALSE
762021	Acute deep venous thrombosis of popliteal vein of left leg	SNOMED	FALSE	FALSE
762010	Acute deep venous thrombosis of right axillary vein	SNOMED	FALSE	FALSE
760877	Acute deep venous thrombosis of right calf	SNOMED	FALSE	FALSE
762013	Acute deep venous thrombosis of right femoral vein	SNOMED	FALSE	FALSE
762018	Acute deep venous thrombosis of right internal jugular vein	SNOMED	FALSE	FALSE
762419	Acute deep venous thrombosis of right lower extremity	SNOMED	FALSE	FALSE
762005	Acute deep venous thrombosis of right upper extremity	SNOMED	FALSE	FALSE
44782745	Acute deep venous thrombosis of thigh	SNOMED	FALSE	FALSE
44782744	Acute deep venous thrombosis of tibial vein	SNOMED	FALSE	FALSE
762026	Acute deep venous thrombosis of tibial vein of left leg	SNOMED	FALSE	FALSE
765156	Acute deep venous thrombosis of tibial vein of right leg	SNOMED	FALSE	FALSE
44782421	Acute deep venous thrombosis of upper extremity	SNOMED	FALSE	FALSE
764016	Acute deep venous thrombosis of upper extremity after coronary artery bypass graft	SNOMED	FALSE	FALSE
44782766	Acute deep venous thrombosis of upper extremity as complication of procedure	SNOMED	FALSE	FALSE
762048	Acute thrombosis of left subclavian vein	SNOMED	FALSE	FALSE
45757410	Acute thrombosis of mesenteric vein	SNOMED	FALSE	FALSE
762049	Acute thrombosis of right subclavian vein	SNOMED	FALSE	FALSE
36712892	Acute thrombosis of splenic vein	SNOMED	FALSE	FALSE
44782762	Acute thrombosis of subclavian vein	SNOMED	FALSE	FALSE
37109253	Bilateral acute deep vein thrombosis of femoral veins	SNOMED	FALSE	FALSE
40478951	Bilateral deep vein thrombosis of lower extremities	SNOMED	FALSE	FALSE
4046884	Deep vein thrombosis of leg related to air travel	SNOMED	FALSE	FALSE
4133004	Deep venous thrombosis	SNOMED	FALSE	FALSE
4181315	Deep venous thrombosis associated with coronary artery bypass graft	SNOMED	FALSE	FALSE
45773536	Deep venous thrombosis of femoropopliteal vein	SNOMED	FALSE	FALSE

763942	Deep venous thrombosis of left lower extremity	SNOMED	FALSE	FALSE
761980	Deep venous thrombosis of left upper extremity	SNOMED	FALSE	FALSE
443537	Deep venous thrombosis of lower extremity	SNOMED	FALSE	FALSE
4133975	Deep venous thrombosis of pelvic vein	SNOMED	FALSE	FALSE
40480555	Deep venous thrombosis of peroneal vein	SNOMED	FALSE	FALSE
4322565	Deep venous thrombosis of profunda femoris vein	SNOMED	FALSE	FALSE
763941	Deep venous thrombosis of right lower extremity	SNOMED	FALSE	FALSE
761928	Deep venous thrombosis of right upper extremity	SNOMED	FALSE	FALSE
4207899	Deep venous thrombosis of tibial vein	SNOMED	FALSE	FALSE
4028057	Deep venous thrombosis of upper extremity	SNOMED	FALSE	FALSE
193512	Embolism and thrombosis of the renal vein	SNOMED	FALSE	FALSE
435565	Embolism and thrombosis of the vena cava	SNOMED	FALSE	FALSE
4119760	Iliofemoral deep vein thrombosis	SNOMED	FALSE	FALSE
4124856	Inferior mesenteric vein thrombosis	SNOMED	FALSE	FALSE
4281689	Phlegmasia alba dolens	SNOMED	FALSE	FALSE
4284538	Phlegmasia cerulea dolens	SNOMED	FALSE	FALSE
4309333	Postoperative deep vein thrombosis	SNOMED	FALSE	FALSE
46285905	Provoked deep vein thrombosis	SNOMED	FALSE	FALSE
4033521	Splenic vein thrombosis	SNOMED	FALSE	FALSE
4055089	Superior mesenteric vein thrombosis	SNOMED	FALSE	FALSE
42538533	Thrombosis of iliac vein	SNOMED	FALSE	FALSE
44811347	Thrombosis of internal jugular vein	SNOMED	FALSE	FALSE
765049	Thrombosis of left peroneal vein	SNOMED	FALSE	FALSE
4317289	Thrombosis of mesenteric vein	SNOMED	FALSE	FALSE
4203836	Thrombosis of subclavian vein	SNOMED	FALSE	FALSE
4175649	Thrombosis of the popliteal vein	SNOMED	FALSE	FALSE
4153353	Traumatic thrombosis of axillary vein	SNOMED	FALSE	FALSE
46285904	Unprovoked deep vein thrombosis	SNOMED	FALSE	FALSE
4221821	Thrombophlebitis of deep veins of lower extremity	SNOMED	FALSE	FALSE
46271900	Recurrent deep vein thrombosis	SNOMED	FALSE	FALSE
4189004	Deep vein thrombosis of leg related to intravenous drug use	SNOMED	FALSE	FALSE

Splanchnic Vein Thrombosis

Concept ID	Concept name	Vocabulary	Is excluded?	Include descendants?
4033521	Splenic vein thrombosis	SNOMED	FALSE	FALSE
196715	Budd-Chiari syndrome	SNOMED	FALSE	FALSE
199837	Portal vein thrombosis	SNOMED	FALSE	FALSE
4317289	Thrombosis of mesenteric vein	SNOMED	FALSE	FALSE
4092406	Portal thrombophlebitis	SNOMED	FALSE	FALSE
36712892	Acute thrombosis of splenic vein	SNOMED	FALSE	FALSE
4173167	Mesenteric embolus	SNOMED	FALSE	FALSE
4144032	Mesenteric thrombus and/or embolus	SNOMED	FALSE	FALSE
45757410	Acute thrombosis of mesenteric vein	SNOMED	FALSE	FALSE

45757409	Chronic thrombosis of mesenteric vein	SNOMED	FALSE	FALSE
4318407	Thrombophlebitis of mesenteric vein	SNOMED	FALSE	FALSE
4124856	Inferior mesenteric vein thrombosis	SNOMED	FALSE	FALSE
4055089	Superior mesenteric vein thrombosis	SNOMED	FALSE	FALSE

Pulmonary embolism

Concept ID	Concept name	Vocabulary	Is excluded?	Include descendants?
4120091	Acute massive pulmonary embolism	SNOMED	FALSE	FALSE
45768439	Acute pulmonary embolism	SNOMED	FALSE	FALSE
45768888	Acute pulmonary thromboembolism	SNOMED	FALSE	FALSE
4309039	Hemorrhagic pulmonary infarction	SNOMED	FALSE	FALSE
762808	Infarction of lung due to embolus	SNOMED	FALSE	FALSE
40480461	Infarction of lung due to iatrogenic pulmonary embolism	SNOMED	FALSE	FALSE
4108681	Postoperative pulmonary embolus	SNOMED	FALSE	FALSE
4091708	Pulmonary air embolism	SNOMED	FALSE	FALSE
440417	Pulmonary embolism	SNOMED	FALSE	FALSE
37109911	Pulmonary embolism due to and following acute myocardial infarction	SNOMED	FALSE	FALSE
37016922	Pulmonary embolism on long-term anticoagulation therapy	SNOMED	FALSE	FALSE
43530605	Pulmonary embolism with pulmonary infarction	SNOMED	FALSE	FALSE
4119608	Pulmonary fat embolism	SNOMED	FALSE	FALSE
254662	Pulmonary infarction	SNOMED	FALSE	FALSE
4253796	Pulmonary microemboli	SNOMED	FALSE	FALSE
45766471	Pulmonary oil microembolism	SNOMED	FALSE	FALSE
4121618	Pulmonary thromboembolism	SNOMED	FALSE	FALSE
4119610	Pulmonary tumor embolism	SNOMED	FALSE	FALSE
4119607	Subacute massive pulmonary embolism	SNOMED	FALSE	FALSE
4119609	Subacute pulmonary fat embolism	SNOMED	FALSE	FALSE
4236271	Recurrent pulmonary embolism	SNOMED	FALSE	FALSE

Thrombocytopenia

- Platelet measurement

Concept ID	Concept name	Vocabulary	Is excluded?	Include descendants?
3007461	Platelets [#/volume] in Blood	LOINC	FALSE	TRUE
3031586	Platelets [#/volume] in Blood by Estimate	LOINC	FALSE	TRUE
3024929	Platelets [#/volume] in Blood by Automated count	LOINC	FALSE	TRUE
3039827	Platelets [#/volume] in Body fluid by Automated count	LOINC	FALSE	TRUE
3024386	Platelet mean volume [Entitic volume] in Blood by Rees-Ecker	LOINC	FALSE	TRUE
4267147	Platelet count	SNOMED	FALSE	TRUE
37393863	Platelet count	SNOMED	FALSE	TRUE

- Thrombocytopenia diagnosis

Concept ID	Concept name	Vocabulary	Is excluded?	Include descendants?
37397537	Beta thalassemia X-linked thrombocytopenia syndrome	SNOMED	FALSE	FALSE
432870	Thrombocytopenic disorder	SNOMED	FALSE	FALSE
46272950	Thrombocytopathy, asplenia and miosis	SNOMED	FALSE	FALSE
44782445	Thrombocytopenia due to alcohol	SNOMED	FALSE	FALSE
42536958	Pancytopenia caused by medication	SNOMED	FALSE	FALSE
40321716	Secondary thrombocytopenia	SNOMED	FALSE	FALSE
37312165	Atypical hemolytic uremic syndrome	SNOMED	FALSE	FALSE
37209558	Pancytopenia caused by immunosuppressant	SNOMED	FALSE	FALSE
37204551	Hereditary isolated aplastic anemia	SNOMED	FALSE	FALSE
37204548	Hereditary thrombocytopenia with normal platelets	SNOMED	FALSE	FALSE
37204520	Bleeding diathesis due to thromboxane synthesis deficiency	SNOMED	FALSE	FALSE
37204478	Pancytopenia due to IKZF1 mutations	SNOMED	FALSE	FALSE
37117164	Revesz syndrome	SNOMED	FALSE	FALSE
37116398	Thyrocerebrorenal syndrome	SNOMED	FALSE	FALSE
37110394	Isolated thrombocytopenia	SNOMED	FALSE	FALSE
37019055	Aplastic anemia co-occurrent with human immunodeficiency virus infection	SNOMED	FALSE	FALSE
37018663	Thrombocytopenia co-occurrent and due to alcoholism	SNOMED	FALSE	FALSE
37017607	Antibody mediated acquired pure red cell aplasia caused by erythropoiesis stimulating agent	SNOMED	FALSE	FALSE
37017165	GATA binding protein 1 related thrombocytopenia with dyserythropoiesis	SNOMED	FALSE	FALSE
37016797	MYH9 related disease	SNOMED	FALSE	FALSE
37016151	Aplastic anemia caused by antineoplastic agent	SNOMED	FALSE	FALSE
36717326	DK phocomelia syndrome	SNOMED	FALSE	FALSE
36716406	Severe fever with thrombocytopenia syndrome virus	SNOMED	FALSE	FALSE
36716047	Radioulnar synostosis with amegakaryocytic thrombocytopenia syndrome	SNOMED	FALSE	FALSE
36715586	Refractory thrombocytopenia	SNOMED	FALSE	FALSE
36715053	Autosomal dominant macrothrombocytopenia	SNOMED	FALSE	FALSE
36713970	WT limb blood syndrome	SNOMED	FALSE	FALSE
36713443	MYH9 macrothrombocytopenia syndrome	SNOMED	FALSE	FALSE
36713112	Pancytopenia due to antineoplastic chemotherapy	SNOMED	FALSE	FALSE
36674972	Macrothrombocytopenia with mitral valve insufficiency	SNOMED	FALSE	FALSE
36674474	Pancytopenia with developmental delay syndrome	SNOMED	FALSE	FALSE
35625536	Ataxia pancytopenia syndrome	SNOMED	FALSE	FALSE
35623407	Adult pure red cell aplasia	SNOMED	FALSE	FALSE
4345236	Parvoviral aplastic crisis	SNOMED	FALSE	FALSE
4338386	Thrombocytopenia due to non-immune destruction	SNOMED	FALSE	FALSE
4316372	HELLP syndrome	SNOMED	FALSE	FALSE

4314802	Kasabach-Merritt syndrome	SNOMED	FALSE	FALSE
4311682	Radial aplasia-thrombocytopenia syndrome	SNOMED	FALSE	FALSE
4305588	Doan-Wright syndrome	SNOMED	FALSE	FALSE
4301602	Thrombotic thrombocytopenic purpura	SNOMED	FALSE	FALSE
4301128	Thrombocytopenia due to diminished platelet production	SNOMED	FALSE	FALSE
4300464	Wiskott-Aldrich autosomal dominant variant syndrome	SNOMED	FALSE	FALSE
4299560	Thrombocytopenic purpura due to defective platelet production	SNOMED	FALSE	FALSE
4298690	Immunologic aplastic anemia	SNOMED	FALSE	FALSE
4292531	Thrombocytopenic purpura due to platelet consumption	SNOMED	FALSE	FALSE
4292425	Sex-linked thrombocytopenia	SNOMED	FALSE	FALSE
4272928	Thrombocytopenia due to hypersplenism	SNOMED	FALSE	FALSE
4264464	Mediterranean macrothrombocytopenia	SNOMED	FALSE	FALSE
4258261	Drug induced thrombotic thrombocytopenic purpura	SNOMED	FALSE	FALSE
4247776	Posttransfusion purpura	SNOMED	FALSE	FALSE
4239484	Acquired pancytopenia	SNOMED	FALSE	FALSE
4235220	Hereditary thrombocytopenic disorder	SNOMED	FALSE	FALSE
4234973	Chronic acquired pure red cell aplasia	SNOMED	FALSE	FALSE
4233407	Megakaryocytic aplasia	SNOMED	FALSE	FALSE
4230266	Autoimmune thrombotic thrombocytopenic purpura	SNOMED	FALSE	FALSE
4226905	Thrombocytopenia associated with AIDS	SNOMED	FALSE	FALSE
4225810	Aplastic anemia associated with AIDS	SNOMED	FALSE	FALSE
4219476	Thrombocytopenia due to defective platelet production	SNOMED	FALSE	FALSE
4218171	Uremic thrombocytopenia	SNOMED	FALSE	FALSE
4214947	Thrombocytopenic purpura associated with metabolic disorder	SNOMED	FALSE	FALSE
4211348	Aplastic anemia associated with pancreatitis	SNOMED	FALSE	FALSE
4204900	Acquired thrombotic thrombocytopenic purpura	SNOMED	FALSE	FALSE
4197574	Dilutional thrombocytopenia	SNOMED	FALSE	FALSE
4188208	Estren-Dameshek anemia	SNOMED	FALSE	FALSE
4186108	Aplastic anemia associated with metabolic alteration	SNOMED	FALSE	FALSE
4185078	Bernard Soulier syndrome	SNOMED	FALSE	FALSE
4184758	Acquired aplastic anemia	SNOMED	FALSE	FALSE
4184200	Secondary aplastic anemia	SNOMED	FALSE	FALSE
4177177	Cellular immunologic aplastic anemia	SNOMED	FALSE	FALSE
4173278	Thrombocytopenia due to blood loss	SNOMED	FALSE	FALSE
4172008	Cyclic thrombocytopenia	SNOMED	FALSE	FALSE
4166754	Perinatal thrombocytopenia	SNOMED	FALSE	FALSE
4159966	Upshaw-Schulman syndrome	SNOMED	FALSE	FALSE
4159749	Idiopathic maternal thrombocytopenia	SNOMED	FALSE	FALSE
4159736	Radiation thrombocytopenia	SNOMED	FALSE	FALSE
4156233	Thrombocytopenia due to sequestration	SNOMED	FALSE	FALSE
4148471	Fanconi’s anemia	SNOMED	FALSE	FALSE
4147049	Thrombocytopenia due to extracorporeal circulation	SNOMED	FALSE	FALSE

4146088	Aplastic anemia due to drugs	SNOMED	FALSE	FALSE
4146086	Constitutional aplastic anemia with malformation	SNOMED	FALSE	FALSE
4145458	Thrombocytopenia due to hypothermia	SNOMED	FALSE	FALSE
4140545	Post infectious thrombocytopenic purpura	SNOMED	FALSE	FALSE
4139555	Thrombocytopenia due to massive blood transfusion	SNOMED	FALSE	FALSE
4137430	Idiopathic thrombocytopenic purpura	SNOMED	FALSE	FALSE
4133984	Alloimmune thrombocytopenia	SNOMED	FALSE	FALSE
4133983	Secondary autoimmune thrombocytopenia	SNOMED	FALSE	FALSE
4133981	Benign gestational thrombocytopenia	SNOMED	FALSE	FALSE
4125496	Pure red cell aplasia, acquired	SNOMED	FALSE	FALSE
4125494	Pancytopenia with pancreatitis	SNOMED	FALSE	FALSE
4123076	Montreal platelet syndrome	SNOMED	FALSE	FALSE
4123075	May-Hegglin anomaly	SNOMED	FALSE	FALSE
4123074	Megakaryocytic thrombocytopenia	SNOMED	FALSE	FALSE
4121265	Mediterranean thrombocytopenia	SNOMED	FALSE	FALSE
4121264	Epstein syndrome	SNOMED	FALSE	FALSE
4120620	Amegakaryocytic thrombocytopenia	SNOMED	FALSE	FALSE
4119134	Thrombocytopenic purpura	SNOMED	FALSE	FALSE
4103532	Immune thrombocytopenia	SNOMED	FALSE	FALSE
4102469	Acute idiopathic thrombocytopenic purpura	SNOMED	FALSE	FALSE
4101603	Thrombocytopenia due to extracorporeal circulation of blood	SNOMED	FALSE	FALSE
4101583	Aplastic anemia due to infection	SNOMED	FALSE	FALSE
4101582	Aplastic anemia due to chronic disease	SNOMED	FALSE	FALSE
4100998	Aplastic anemia due to toxic cause	SNOMED	FALSE	FALSE
4098148	Thrombocytopenia due to drugs	SNOMED	FALSE	FALSE
4098145	Idiopathic aplastic anemia	SNOMED	FALSE	FALSE
4098028	Transient acquired pure red cell aplasia	SNOMED	FALSE	FALSE
4098027	Aplastic anemia due to radiation	SNOMED	FALSE	FALSE
4082738	Autoimmune pancytopenia	SNOMED	FALSE	FALSE
4077348	Pancytopenia-dysmelia	SNOMED	FALSE	FALSE
4031699	Humoral immunologic aplastic anemia	SNOMED	FALSE	FALSE
4028065	Autoimmune thrombocytopenia	SNOMED	FALSE	FALSE
4027374	Alloimmune platelet transfusion refractoriness	SNOMED	FALSE	FALSE
4009307	Heparin-induced thrombocytopenia with thrombosis	SNOMED	FALSE	FALSE
4000065	Drug-induced immune thrombocytopenia	SNOMED	FALSE	FALSE
441264	Primary thrombocytopenia	SNOMED	FALSE	FALSE
440982	Wiskott-Aldrich syndrome	SNOMED	FALSE	FALSE
440372	Acquired thrombocytopenia	SNOMED	FALSE	FALSE
436956	Evans syndrome	SNOMED	FALSE	FALSE
433749	Heparin-induced thrombocytopenia	SNOMED	FALSE	FALSE
432881	Pancytopenia	SNOMED	FALSE	FALSE
318397	Chronic idiopathic thrombocytopenic purpura	SNOMED	FALSE	FALSE
140681	Constitutional aplastic anemia	SNOMED	FALSE	FALSE
138723	Acquired red cell aplasia	SNOMED	FALSE	FALSE
137829	Aplastic anemia	SNOMED	FALSE	FALSE

- Thrombocytopenic purpura

Concept ID	Concept name	Vocabulary	Is excluded?	Include descendants?
4119134	Thrombocytopenic purpura	SNOMED	FALSE	FALSE
4301602	Thrombotic thrombocytopenic purpura	SNOMED	FALSE	FALSE
4299560	Thrombocytopenic purpura due to defective platelet production	SNOMED	FALSE	FALSE
4292531	Thrombocytopenic purpura due to platelet consumption	SNOMED	FALSE	FALSE
4258261	Drug induced thrombotic thrombocytopenic purpura	SNOMED	FALSE	FALSE
4247776	Posttransfusion purpura	SNOMED	FALSE	FALSE
4230266	Autoimmune thrombotic thrombocytopenic purpura	SNOMED	FALSE	FALSE
4214947	Thrombocytopenic purpura associated with metabolic disorder	SNOMED	FALSE	FALSE
4204900	Acquired thrombotic thrombocytopenic purpura	SNOMED	FALSE	FALSE
4159966	Upshaw-Schulman syndrome	SNOMED	FALSE	FALSE
4140545	Post infectious thrombocytopenic purpura	SNOMED	FALSE	FALSE
4137430	Idiopathic thrombocytopenic purpura	SNOMED	FALSE	FALSE
4102469	Acute idiopathic thrombocytopenic purpura	SNOMED	FALSE	FALSE
318397	Chronic idiopathic thrombocytopenic purpura	SNOMED	FALSE	FALSE
313800	Thrombotic microangiopathy	SNOMED	FALSE	FALSE

- Immune thrombocytopenia

Concept ID	Concept name	Vocabulary	Is excluded?	Include descendants?
4103532	Immune thrombocytopenia	SNOMED	FALSE	FALSE
4137430	Idiopathic thrombocytopenic purpura	SNOMED	FALSE	FALSE
4133984	Alloimmune thrombocytopenia	SNOMED	FALSE	FALSE
4133983	Secondary autoimmune thrombocytopenia	SNOMED	FALSE	FALSE
4102469	Acute idiopathic thrombocytopenic purpura	SNOMED	FALSE	FALSE
4028065	Autoimmune thrombocytopenia	SNOMED	FALSE	FALSE
4027374	Alloimmune platelet transfusion refractoriness	SNOMED	FALSE	FALSE
4009307	Heparin-induced thrombocytopenia with thrombosis	SNOMED	FALSE	FALSE
4000065	Drug-induced immune thrombocytopenia	SNOMED	FALSE	FALSE
436956	Evans syndrome	SNOMED	FALSE	FALSE
433749	Heparin-induced thrombocytopenia	SNOMED	FALSE	FALSE
318397	Chronic idiopathic thrombocytopenic purpura	SNOMED	FALSE	FALSE

Ischemic stroke

Concept ID	Concept name	Vocabulary	Is excluded?	Include descendants?
4045735	Anterior cerebral circulation infarction	SNOMED	FALSE	FALSE
4031045	Anterior choroidal artery syndrome	SNOMED	FALSE	FALSE
761110	Bilateral cerebral infarction due to precererbral arterial occlusion	SNOMED	FALSE	FALSE

4110189	Cerebral infarct due to thrombosis of precerebral arteries	SNOMED	FALSE	FALSE
443454	Cerebral infarction	SNOMED	FALSE	FALSE
762951	Cerebral infarction due to anterior cerebral artery occlusion	SNOMED	FALSE	FALSE
765515	Cerebral infarction due to basilar artery stenosis	SNOMED	FALSE	FALSE
43530683	Cerebral infarction due to carotid artery occlusion	SNOMED	FALSE	FALSE
762933	Cerebral infarction due to cerebral artery occlusion	SNOMED	FALSE	FALSE
762937	Cerebral infarction due to cerebral venous thrombosis	SNOMED	FALSE	FALSE
4111714	Cerebral infarction due to cerebral venous thrombosis, non-pyogenic	SNOMED	FALSE	FALSE
4108356	Cerebral infarction due to embolism of cerebral arteries	SNOMED	FALSE	FALSE
45772786	Cerebral infarction due to embolism of middle cerebral artery	SNOMED	FALSE	FALSE
4110190	Cerebral infarction due to embolism of precerebral arteries	SNOMED	FALSE	FALSE
762935	Cerebral infarction due to internal carotid artery occlusion	SNOMED	FALSE	FALSE
763015	Cerebral infarction due to middle cerebral artery occlusion	SNOMED	FALSE	FALSE
46273649	Cerebral infarction due to occlusion of basilar artery	SNOMED	FALSE	FALSE
35610084	Cerebral infarction due to occlusion of cerebral artery	SNOMED	FALSE	FALSE
46270031	Cerebral infarction due to occlusion of precerebral artery	SNOMED	FALSE	FALSE
762934	Cerebral infarction due to posterior cerebral artery occlusion	SNOMED	FALSE	FALSE
43531607	Cerebral infarction due to stenosis of carotid artery	SNOMED	FALSE	FALSE
35610085	Cerebral infarction due to stenosis of cerebral artery	SNOMED	FALSE	FALSE
46270381	Cerebral infarction due to stenosis of precerebral artery	SNOMED	FALSE	FALSE
4110192	Cerebral infarction due to thrombosis of cerebral arteries	SNOMED	FALSE	FALSE
45767658	Cerebral infarction due to thrombosis of middle cerebral artery	SNOMED	FALSE	FALSE
44782773	Cerebral infarction due to vertebral artery occlusion	SNOMED	FALSE	FALSE
46270380	Cerebral infarction due to vertebral artery stenosis	SNOMED	FALSE	FALSE
37110678	Cerebral ischemic stroke due to occlusion of extracranial large artery	SNOMED	FALSE	FALSE
37110679	Cerebral ischemic stroke due to stenosis of extracranial large artery	SNOMED	FALSE	FALSE
4043731	Infarction - precerebral	SNOMED	FALSE	FALSE
4131383	Infarction of basal ganglia	SNOMED	FALSE	FALSE
4046237	Infarction of optic radiation	SNOMED	FALSE	FALSE
4119140	Infarction of visual cortex	SNOMED	FALSE	FALSE
4141405	Left sided cerebral infarction	SNOMED	FALSE	FALSE

37116473	Multifocal cerebral infarction due to and following procedure on cardiovascular system	SNOMED	FALSE	FALSE
4077086	Occipital cerebral infarction	SNOMED	FALSE	FALSE
4046359	Partial anterior cerebral circulation infarction	SNOMED	FALSE	FALSE
4319146	Pituitary infarction	SNOMED	FALSE	FALSE
4146185	Right sided cerebral infarction	SNOMED	FALSE	FALSE
36717605	Silent cerebral infarct	SNOMED	FALSE	FALSE
4142739	Thalamic infarction	SNOMED	FALSE	FALSE
4046358	Total anterior cerebral circulation infarction	SNOMED	FALSE	FALSE
372924	Cerebral artery occlusion	SNOMED	FALSE	FALSE

Myocardial infarction

Concept ID	Concept name	Vocabulary	Is excluded?	Include descendants?
4119457	Acute Q wave infarction - anterolateral	SNOMED	FALSE	FALSE
4119943	Acute Q wave infarction - anteroseptal	SNOMED	FALSE	FALSE
4121464	Acute Q wave infarction - inferior	SNOMED	FALSE	FALSE
4121465	Acute Q wave infarction - inferolateral	SNOMED	FALSE	FALSE
4124684	Acute Q wave infarction - lateral	SNOMED	FALSE	FALSE
4119948	Acute Q wave infarction - widespread	SNOMED	FALSE	FALSE
4126801	Acute Q wave myocardial infarction	SNOMED	FALSE	FALSE
4296653	Acute ST segment elevation myocardial infarction	SNOMED	FALSE	FALSE
46270162	Acute ST segment elevation myocardial infarction due to left coronary artery occlusion	SNOMED	FALSE	FALSE
761737	Acute ST segment elevation myocardial infarction due to occlusion of circumflex coronary artery	SNOMED	FALSE	FALSE
46270163	Acute ST segment elevation myocardial infarction due to right coronary artery occlusion	SNOMED	FALSE	FALSE
43020460	Acute ST segment elevation myocardial infarction involving left anterior descending coronary artery	SNOMED	FALSE	FALSE
45766076	Acute ST segment elevation myocardial infarction of anterior wall involving right ventricle	SNOMED	FALSE	FALSE
761736	Acute ST segment elevation myocardial infarction of anteroapical wall	SNOMED	FALSE	FALSE
46270159	Acute ST segment elevation myocardial infarction of anterolateral wall	SNOMED	FALSE	FALSE
46270160	Acute ST segment elevation myocardial infarction of anteroseptal wall	SNOMED	FALSE	FALSE
45766116	Acute ST segment elevation myocardial infarction of inferior wall	SNOMED	FALSE	FALSE
45766151	Acute ST segment elevation myocardial infarction of inferior wall involving right ventricle	SNOMED	FALSE	FALSE
35611570	Acute ST segment elevation myocardial infarction of inferolateral wall	SNOMED	FALSE	FALSE
35611571	Acute ST segment elevation myocardial infarction of inferoposterior wall	SNOMED	FALSE	FALSE
46274044	Acute ST segment elevation myocardial infarction of lateral wall	SNOMED	FALSE	FALSE

46270161	Acute ST segment elevation myocardial infarction of posterior wall	SNOMED	FALSE	FALSE
46273495	Acute ST segment elevation myocardial infarction of posterobasal wall	SNOMED	FALSE	FALSE
46270158	Acute ST segment elevation myocardial infarction of posterolateral wall	SNOMED	FALSE	FALSE
46270164	Acute ST segment elevation myocardial infarction of septum	SNOMED	FALSE	FALSE
45766075	Acute anterior ST segment elevation myocardial infarction	SNOMED	FALSE	FALSE
4178129	Acute anteroapical myocardial infarction	SNOMED	FALSE	FALSE
4267568	Acute anteroseptal myocardial infarction	SNOMED	FALSE	FALSE
312327	Acute myocardial infarction	SNOMED	FALSE	FALSE
44782769	Acute myocardial infarction due to left coronary artery occlusion	SNOMED	FALSE	FALSE
44782712	Acute myocardial infarction due to right coronary artery occlusion	SNOMED	FALSE	FALSE
45766115	Acute myocardial infarction during procedure	SNOMED	FALSE	FALSE
434376	Acute myocardial infarction of anterior wall	SNOMED	FALSE	FALSE
45766150	Acute myocardial infarction of anterior wall involving right ventricle	SNOMED	FALSE	FALSE
438438	Acute myocardial infarction of anterolateral wall	SNOMED	FALSE	FALSE
4243372	Acute myocardial infarction of apical-lateral wall	SNOMED	FALSE	FALSE
4108669	Acute myocardial infarction of atrium	SNOMED	FALSE	FALSE
4151046	Acute myocardial infarction of basal-lateral wall	SNOMED	FALSE	FALSE
4275436	Acute myocardial infarction of high lateral wall	SNOMED	FALSE	FALSE
438170	Acute myocardial infarction of inferior wall	SNOMED	FALSE	FALSE
45771322	Acute myocardial infarction of inferior wall involving right ventricle	SNOMED	FALSE	FALSE
438447	Acute myocardial infarction of inferolateral wall	SNOMED	FALSE	FALSE
441579	Acute myocardial infarction of inferoposterior wall	SNOMED	FALSE	FALSE
436706	Acute myocardial infarction of lateral wall	SNOMED	FALSE	FALSE
4324413	Acute myocardial infarction of posterobasal wall	SNOMED	FALSE	FALSE
4051874	Acute myocardial infarction of posterolateral wall	SNOMED	FALSE	FALSE
4303359	Acute myocardial infarction of septum	SNOMED	FALSE	FALSE
4147223	Acute myocardial infarction with rupture of ventricle	SNOMED	FALSE	FALSE
4145721	Acute non-Q wave infarction	SNOMED	FALSE	FALSE
4119944	Acute non-Q wave infarction - anterolateral	SNOMED	FALSE	FALSE
4119456	Acute non-Q wave infarction - anteroseptal	SNOMED	FALSE	FALSE
4119945	Acute non-Q wave infarction - inferior	SNOMED	FALSE	FALSE

4119946	Acute non-Q wave infarction - inferolateral	SNOMED	FALSE	FALSE
4121466	Acute non-Q wave infarction - lateral	SNOMED	FALSE	FALSE
4124685	Acute non-Q wave infarction - widespread	SNOMED	FALSE	FALSE
4270024	Acute non-ST segment elevation myocardial infarction	SNOMED	FALSE	FALSE
35610091	Acute nontransmural myocardial infarction	SNOMED	FALSE	FALSE
319039	Acute posterior myocardial infarction	SNOMED	FALSE	FALSE
444406	Acute subendocardial infarction	SNOMED	FALSE	FALSE
35610093	Acute transmural myocardial infarction	SNOMED	FALSE	FALSE
4119947	Acute widespread myocardial infarction	SNOMED	FALSE	FALSE
37109912	Arrhythmia due to and following acute myocardial infarction	SNOMED	FALSE	FALSE
438172	Atrial septal defect due to and following acute myocardial infarction	SNOMED	FALSE	FALSE
4124687	Cardiac rupture due to and following acute myocardial infarction	SNOMED	FALSE	FALSE
4215259	First myocardial infarction	SNOMED	FALSE	FALSE
4108678	Hemopericardium due to and following acute myocardial infarction	SNOMED	FALSE	FALSE
4173632	Microinfarct of heart	SNOMED	FALSE	FALSE
45771327	Mitral valve regurgitation due to acute myocardial infarction with papillary muscle and chordal rupture	SNOMED	FALSE	FALSE
45766214	Mitral valve regurgitation due to acute myocardial infarction without papillary muscle and chordal rupture	SNOMED	FALSE	FALSE
45766212	Mitral valve regurgitation due to and following acute myocardial infarction	SNOMED	FALSE	FALSE
4323202	Mixed myocardial ischemia and infarction	SNOMED	FALSE	FALSE
4329847	Myocardial infarction	SNOMED	FALSE	FALSE
37309626	Myocardial infarction due to demand ischemia	SNOMED	FALSE	FALSE
4170094	Myocardial infarction in recovery phase	SNOMED	FALSE	FALSE
4200113	Non-Q wave myocardial infarction	SNOMED	FALSE	FALSE
4030582	Postoperative myocardial infarction	SNOMED	FALSE	FALSE
35610087	Postoperative nontransmural myocardial infarction	SNOMED	FALSE	FALSE
4206867	Postoperative subendocardial myocardial infarction	SNOMED	FALSE	FALSE
35610089	Postoperative transmural myocardial infarction	SNOMED	FALSE	FALSE
4207921	Postoperative transmural myocardial infarction of anterior wall	SNOMED	FALSE	FALSE
4209541	Postoperative transmural myocardial infarction of inferior wall	SNOMED	FALSE	FALSE
37109911	Pulmonary embolism due to and following acute myocardial infarction	SNOMED	FALSE	FALSE
4108679	Rupture of cardiac wall without hemopericardium as current complication following acute myocardial infarction	SNOMED	FALSE	FALSE
4108219	Rupture of chordae tendinae due to and following acute myocardial infarction	SNOMED	FALSE	FALSE
4124686	Silent myocardial infarction	SNOMED	FALSE	FALSE

765132	Subendocardial myocardial infarction	SNOMED	FALSE	FALSE
45766114	Subsequent ST segment elevation myocardial infarction	SNOMED	FALSE	FALSE
45766113	Subsequent ST segment elevation myocardial infarction of anterior wall	SNOMED	FALSE	FALSE
45773170	Subsequent ST segment elevation myocardial infarction of inferior wall	SNOMED	FALSE	FALSE
4108217	Subsequent myocardial infarction	SNOMED	FALSE	FALSE
4108677	Subsequent myocardial infarction of anterior wall	SNOMED	FALSE	FALSE
4108218	Subsequent myocardial infarction of inferior wall	SNOMED	FALSE	FALSE
45766241	Subsequent non-ST segment elevation myocardial infarction	SNOMED	FALSE	FALSE
4108680	Thrombosis of atrium, auricular appendage, and ventricle due to and following acute myocardial infarction	SNOMED	FALSE	FALSE
439693	True posterior myocardial infarction	SNOMED	FALSE	FALSE
37109910	Ventricular aneurysm due to and following acute myocardial infarction	SNOMED	FALSE	FALSE

Other arterial thromboembolism

- Intestinal infarction

Concept ID	Concept name	Vocabulary	Is excluded?	Include descendants?
4195665	Gastrointestinal tract vascular insufficiency	SNOMED	FALSE	FALSE
4148299	Ischemic colitis	SNOMED	FALSE	FALSE
4173167	Mesenteric embolus	SNOMED	FALSE	FALSE
4317289	Thrombosis of mesenteric vein	SNOMED	FALSE	FALSE
4319280	Acute bowel infarction	SNOMED	FALSE	FALSE
4144032	Mesenteric thrombus and/or embolus	SNOMED	FALSE	FALSE
45757410	Acute thrombosis of mesenteric vein	SNOMED	FALSE	FALSE
45757409	Chronic thrombosis of mesenteric vein	SNOMED	FALSE	FALSE
44811741	Acute ischaemia of large intestine	SNOMED	FALSE	FALSE
44811740	Acute ischaemia of small intestine	SNOMED	FALSE	FALSE
37117790	Insufficiency of mesenteric artery	SNOMED	FALSE	FALSE
37016198	Epiploic appendagitis	SNOMED	FALSE	FALSE
35622081	Nongangrenous ischemic colitis	SNOMED	FALSE	FALSE
35622080	Gangrenous ischemic colitis	SNOMED	FALSE	FALSE
4345926	Abdominal angina	SNOMED	FALSE	FALSE
4342767	Transient ischemic colitis	SNOMED	FALSE	FALSE
4341648	Hemorrhagic infarction of intestine	SNOMED	FALSE	FALSE
4341646	Occlusive mesenteric ischemia	SNOMED	FALSE	FALSE
4340939	Non-occlusive mesenteric ischemia	SNOMED	FALSE	FALSE
4340378	Transmural infarction of intestine	SNOMED	FALSE	FALSE
4340375	Focal segmental ischemia of small intestine	SNOMED	FALSE	FALSE
4318537	Large bowel gangrene	SNOMED	FALSE	FALSE
4318407	Thrombophlebitis of mesenteric vein	SNOMED	FALSE	FALSE
4240850	Acute ischemic enterocolitis	SNOMED	FALSE	FALSE
4239942	Embolic mesenteric infarction	SNOMED	FALSE	FALSE
4237654	Ischemic enterocolitis	SNOMED	FALSE	FALSE
4215949	Nonocclusive intestinal infarction	SNOMED	FALSE	FALSE
4214720	Thrombotic mesenteric infarction	SNOMED	FALSE	FALSE
4192856	Acute ischemic colitis	SNOMED	FALSE	FALSE

4188336	Chronic ischemic enterocolitis	SNOMED	FALSE	FALSE
4174014	Inferior mesenteric artery embolus	SNOMED	FALSE	FALSE
4149013	Mesenteric infarction	SNOMED	FALSE	FALSE
4148257	Chronic gastrointestinal tract vascular insufficiency	SNOMED	FALSE	FALSE
4148256	Acute GIT vascular insufficiency	SNOMED	FALSE	FALSE
4124856	Inferior mesenteric vein thrombosis	SNOMED	FALSE	FALSE
4055089	Superior mesenteric vein thrombosis	SNOMED	FALSE	FALSE
4055025	Superior mesenteric artery embolus	SNOMED	FALSE	FALSE
4045408	Ischemic stricture of intestine	SNOMED	FALSE	FALSE
201894	Acute vascular insufficiency of intestine	SNOMED	FALSE	FALSE
192673	Vascular insufficiency of intestine	SNOMED	FALSE	FALSE
